# Highly Specific Miniaturized Fluorescent Monoacylglycerol Lipase Probes Enable Translational Research

**DOI:** 10.1021/jacs.4c15223

**Published:** 2025-03-10

**Authors:** Axel Hentsch, Mónica Guberman, Silke Radetzki, Sofia Kaushik, Mirjam Huizenga, Yingfang He, Jörg Contzen, Bernd Kuhn, Jörg Benz, Maria Schippers, Jerome Paul, Lea Leibrock, Ludovic Collin, Matthias Wittwer, Andreas Topp, Fionn O’Hara, Dominik Heer, Remo Hochstrasser, Julie Blaising, Jens P. von Kries, Linjing Mu, Mario van der Stelt, Philipp Mergenthaler, Noa Lipstein, Uwe Grether, Marc Nazaré

**Affiliations:** † Leibniz Forschungsinstitut für Molekulare Pharmakologie, Campus Berlin-Buch, 13125 Berlin, Germany; ‡ Division of Drug Discovery and Safety, Leiden Academic Centre for Drug Research, Leiden University, 2333 CC Leiden, The Netherlands; § Charité—Universitätsmedizin Berlin, Center for Stroke Research, 10117 Berlin, Germany; ∥ Charité—Universitätsmedizin Berlin, Dept. of Neurology with Experimental Neurology, 10117 Berlin, Germany; ⊥ University of Oxford, Radcliffe Department of Medicine, OX3 9DU Oxford, United Kingdom; # ETH Zürich, Institute of Pharmaceutical Sciences, Vladimir-Prelog-Weg 4, 8093 Zürich, Switzerland; ∇ Roche Pharma Research & Early Development, 4070 Basel, Switzerland

## Abstract

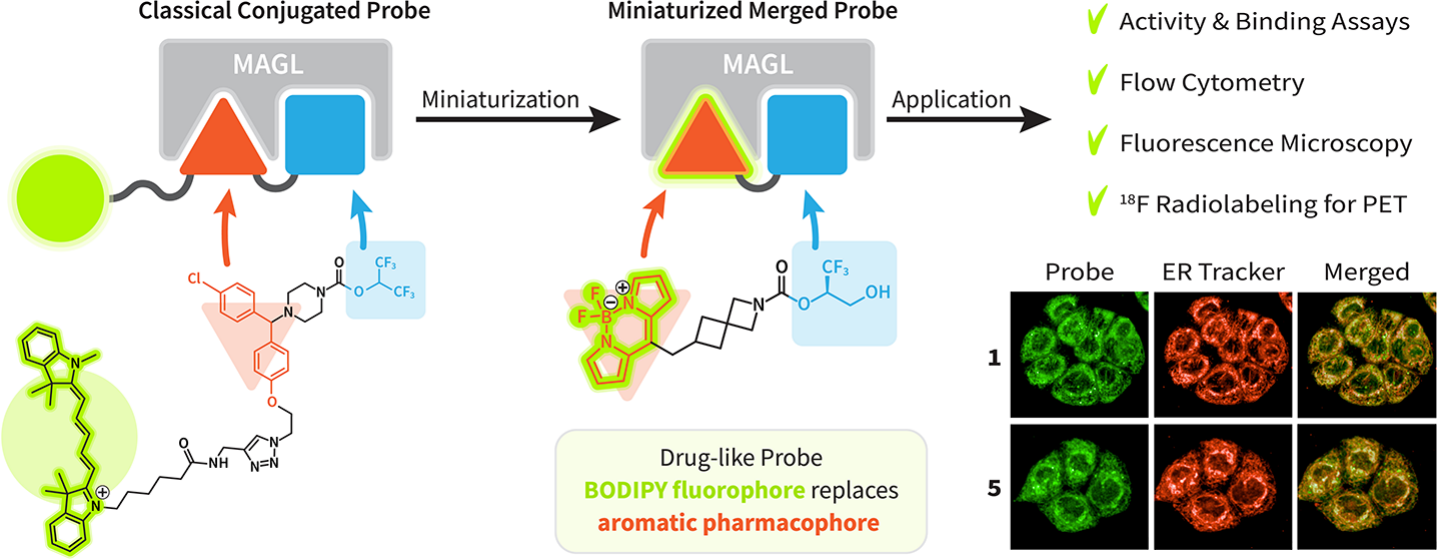

Monoacylglycerol lipase (MAGL) is the pivotal catabolic enzyme responsible for signal termination in the endocannabinoid system. Inhibition of MAGL offers unique advantages over the direct activation of cannabinoid receptors in treating cancer, metabolic disorders, and inflammatory diseases. Although specific fluorescent molecular imaging probes are commonly used for the real-time analysis of the localization and distribution of drug targets in cells, they are almost invariably composed of a linker connecting the pharmacophore with a large fluorophore. In this study, we have developed miniaturized fluorescent probes targeting MAGL by incorporating a highly fluorescent boron-dipyrromethene (BODIPY) moiety into the inhibitor structure that interacts with the MAGL active site. These miniaturized fluorescent probes exhibit favorable drug-like properties such as high solubility and permeability, picomolar potency for MAGL across various species, and high cell selectivity and specificity. A range of translational investigations were conducted, including cell-free fluorescence polarization assays, fluorescence-activated cell sorting analysis, and confocal fluorescence microscopy of live cancer cells, live primary neurons, and human-induced pluripotent stem cell-derived brain organoids. Furthermore, the application of red-shifted analogs or ^18^F positron emission labeling illustrated the significant versatility and adaptability of the fluorescent ligands in various experimental contexts.

## Introduction

Monoacylglycerol lipase (MAGL) is the key metabolic serine hydrolase in the endocannabinoid system (eCS) that significantly influences the retrograde cannabinoid signaling pathways.^1^ MAGL is essential for intracellular signal termination as it hydrolyzes endogenous 2-arachidonoylglycerol (2-AG) to arachidonic acid (AA). This process results in the deactivation of cannabinoid receptors and stimulates the biosynthesis of inflammatory and pain-mediating secondary messengers of the eicosanoid signaling system via AA release.^1−3^ Therefore, MAGL serves as a central node in various physiological and pathophysiological processes by affecting nociception, learning, mood, appetite regulation, addiction, immune responses, and lipid metabolism.^4−7^ Inhibition of MAGL results in the elevation of 2-AG levels, thus mimicking the cannabinoid effects but potentially limiting the adverse effects of cannabinoid ligands.^8−10^ High expression levels of MAGL are found in the brain, predominantly in the hippocampus and cortex,^1^ where it is responsible for 85% of 2-AG hydrolysis activity.^11^ MAGL is ubiquitously expressed in the periphery, however, its influence on 2-AG and AA levels varies considerably.^12^ Therefore, MAGL is a key therapeutic target for various inflammatory diseases,^5,13,14^ cancer proliferation,^4,15^ neuropathic pain,^16,17^ and metabolic disorders.^6,7^

**Scheme 1:**
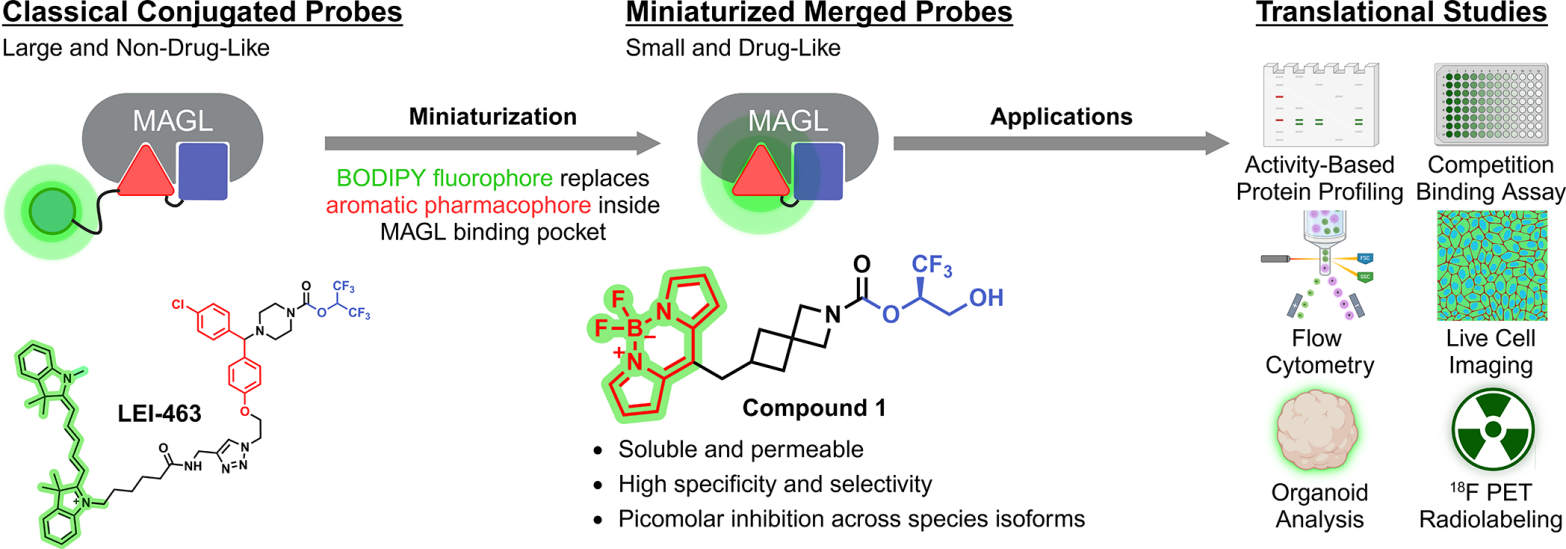
Miniaturization Approach by the Partial Replacement of the Monoacylglycerol Lipase (MAGL) Pharmacophore with the Boron-Dipyrromethene (BODIPY) Fluorophore Comparison of the miniaturized fluorescent probe **1** with the highly lipophilic, classically constructed fluorescent probe **LEI-463**. The miniaturized probes exhibit highly drug-like properties. Their versatility is showcased in various experimental translational settings of clinical relevance, such as activity-based profiling, live cell imaging, flow cytometry and radiolabeling.

Despite the recognized physiological relevance and therapeutic potential of MAGL, its cellular localization, distribution, and trafficking within the endocannabinoid signaling system remain unclear.^3,18^ A persisting lack of information on its expression dynamics, lifetime, subcellular and tissue distribution, and drug–target engagement hinders effective drug development and clinical translation.^6,7^ Distinct compartmental localization in proximity to cannabinoid receptor 1 and membrane association, were suggested to be responsible for the efficient processing of endogenous 2-AG,^18^ but remain elusive. This is partly due to the lack of appropriate imaging tools for the real-time characterization of MAGL in living systems at a subcellular resolution. Small-molecule imaging probes are powerful, real-time tools for the visualization and quantification of physiological processes that can greatly advance drug development efforts.^19,20^ In contrast to antibodies, they can stain cells without fixation and permeabilization techniques and label endogenous rather than overexpressed proteins, thereby facilitating basic or translational investigations under physiological conditions.^19,20^ Additionally, interspecies differences generally pose no obstacles to their application. Currently, the visualization of MAGL is mainly restricted to immunohistochemical approaches,^21,22^ green fluorescent protein-fusion proteins,^23^ positron emission tomography (PET) tracers,^22,24−26^ and only two fluorescently labeled irreversible probes, JW912^27^ and LEI-463^28^ (Scheme 1), of which only the latter is selective for MAGL. Additionally, fluorogenic lipid substrates have been used in high-throughput screens to detect MAGL hydrolysis activity.^29−31^

Currently, the field is lacking reversible fluorescent MAGL probes that possess time-independent affinity and labeling capabilities. These attributes are crucial for the real-time and accurate characterization of MAGL’s functional dynamics at endogenous expression levels within its native cellular environment. To fully comprehend MAGL’s functions, it is necessary to study the enzyme in its native environment within cells, tissues, and organisms in real time. This approach is devoid of genetic editing and ensures a more accurate understanding of physiological functions. Small molecule-based probes can be used in various pharmacological models, ranging from isolated proteins to whole organisms, in the early stages of target validation and clinical trials. However, their use has many limitations owing to the inherent drawbacks of their construction principle, involving a small-molecule ligand, linker, and fluorophore (Scheme 1).

Classical small-molecule fluorescent probes frequently incorporate a fluorescent dye moiety considerably larger than the target recognition element. This disparity often compromises the advantageous properties associated with the parent drug-derived ligand.^32^ Such a probe design typically results in large, lipophilic, and nondrug-like structures. Although the linker is usually necessary to avoid unfavorable interactions with the targeted protein, it causes detrimental changes in size and polarity, preventing efficient cell permeation and increasing nonspecific interactions with other cellular components.^33^ Miniaturized fluorophores have been introduced to minimize nonproductive interactions and other unfavorable features resulting from fluorophore labeling.^34^ Here, we report the conception and systematic investigation of miniaturized probes by the rational incorporation of a fluorophore unit into the ligand structure for productive contribution to protein–ligand interactions and overall binding affinity.^35−37^ To overcome the issues arising from classical fluorophore labeling strategies, we designed reversible and irreversible, inherently fluorescent, miniaturized MAGL probes exhibiting drug-like properties. The probe optimization was achieved by merging a bright, photostable, and uncharged boron-dipyrromethene (BODIPY) fluorophore^38^ with the drug structure (see Scheme 1). The chemotypes explored herein may facilitate the development of a drug-like probe type for the elucidation of the eCS and imaging in general.

## Results and Discussion

### Design and Synthesis of Miniaturized Fluorescent MAGL Probes

The design strategy for our probes encompassed the structural integration of a pharmacophore moiety with a fluorescent reporter unit, as illustrated in Scheme 1. This approach yielded probes of significantly reduced size, adhering to all established criteria of drug-likeness.^39,40^ These criteria included a low molecular weight (MW), a balanced number of hydrogen bond donors and acceptors (HBD, HBA), low lipophilicity (clogP), a minimal number of rotatable bonds (nRotB), and a small topological polar surface area (tPSA) (see Table 1 and SI, Table S2). These are essential characteristics to facilitate membrane permeation,^41^ reduce nonspecific protein binding, and improve solubility and overall bioavailability.^39,40^ These physicochemical properties are particularly important for addressing intracellular enzymes, such as MAGL.

**Table 1:** In Vitro Half-Maximal Inhibitory Concentrations (IC_50_) Values and Physicochemical Properties of the Most Promising Fluorescent Probes

	IC_50_ [nM]					physicochemical properties					
Cmpd.	hMAGL^a^	mMAGL^a^	rMAGL^a^	cMAGL^a^	nanoBRET^b^	MW [g/mol]	HBA/HBD	clogP^c^	tPSA^c^ [Å^2^]	nRotB	Sol.^d^ [μg/mL]
**1**	0.28	0.21	0.36	0.87	276	457	3/1	2.06	63	7	19
**1a**	0.47	0.31	0.44	0.40	n.a.	522	3/2	2.61	79	8	<1.0
**2**	0.39	0.33	0.20	0.24	720	495	2/0	3.45	43	6	<3.0
**2a**	0.98	0.79	0.68	0.63	n.a.	560	2/1	3.99	59	7	<1.0
**3**	0.83	0.96	3.77	3.86	557	483	3/1	1.24	75	2	139
**4**	0.94	1.33	2.40	1.14	196	483	3/1	0.61	75	2	112
**4a**	0.14	0.18	0.47	0.20	n.a.	548	3/2	1.08	91	3	<0.30
**5**	0.58	1.03	0.98	0.50	85	531	3/0	1.99	68	4	46
**5a**	0.73	0.90	2.23	0.77	n.a.	596	3/1	3.40	70	5	<0.10

a Native mass spectrometry (MS) substrate assay of purified human (h), mouse (m), rat (r), and cynomolgus monkey (c) MAGL enzymes in vitro; note that IC_50_ values of covalent inhibitors (**1**, **1a**, **2**, and **2a**) are in general time-dependent.

b Cellular nanoluciferase (Nluc)-based bioluminescence resonance energy transfer (nanoBRET) assay.^58^ IC_50_ value determination was performed in triplicate. (See SI, Table S1 for the standard error of the mean [SEM]).

c Computational descriptors according to SwissADME.^59^

d Experimental kinetic solubility in 50 mM phosphate buffer of pH 6.5.^60^ n.a. = not applicable. The pyrrole-substituted BODIPY derivatives **2a**, **4a** and **5a** could not be assessed via the nanoBRET assay due to fluorophore interference as it formed a BRET pair with nanoluciferase,^58^ thereby giving false-negative results.

**Scheme 2:**
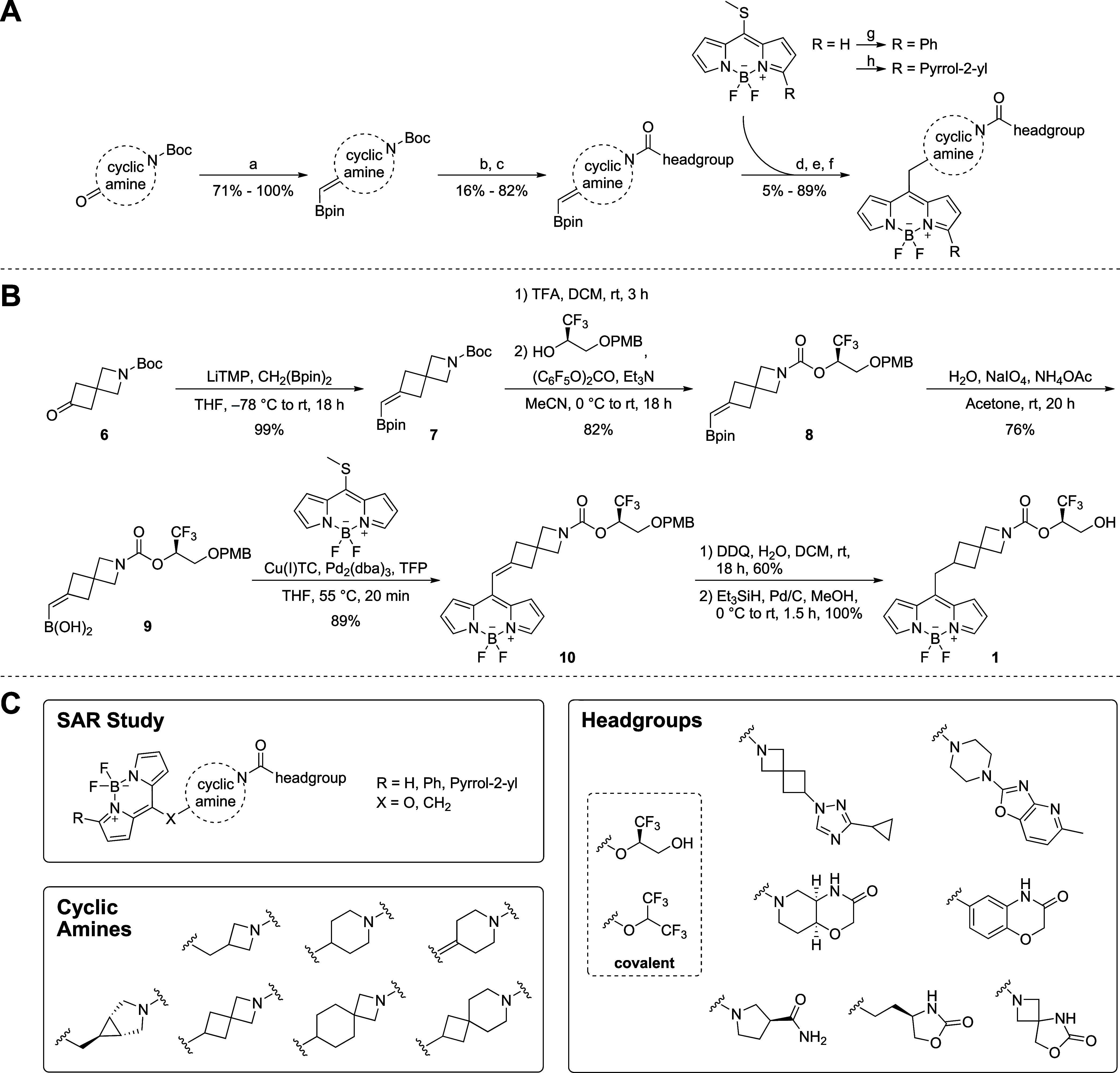
Synthetic Access to BODIPY MAGL Probes (A) General synthetic approach for 8-methylene substituted fluorescent BODIPY MAGL probes involves two key connection steps. The first step is the formation of an amide or urea (non-covalent inhibitor) or carbamate (covalent inhibitor) to connect the headgroup to the cyclic amine and the second step is a Liebeskind–Srogl cross-coupling (LSCC) reaction to attach the BODIPY moiety. Reaction conditions: (a) LiTMP, CH_2_(Bpin)_2_, THF, –78 °C to rt, 18 h. (b) TFA/DCM 1:4, rt, 3 h. (c) Various coupling reactions using activated carbamates (see SI for further information). (d) H_2_O, NH_4_OAc, NaIO_4_, acetone, rt, 16–24 h. (e) Cu(I)TC, Pd_2_(dba)_3_, TFP, THF, 55 °C, 10–90 min. (f) Et_3_SiH, Pd/C, MeOH, 0 °C to rt, 10–120 min. (g) PhNH_2_, *t*-BuONO, MeCN, 40 °C, 16 h. (h) Neat pyrrole, MW, 150 °C, 2–3 h. (B) Synthesis of irreversible, covalent compound **1**. Reaction conditions and yields are indicated in the synthetic sequence. (C) Overview of the examined SAR (for complete structures and SAR data see SI, Figure S1).

**Scheme 3:**
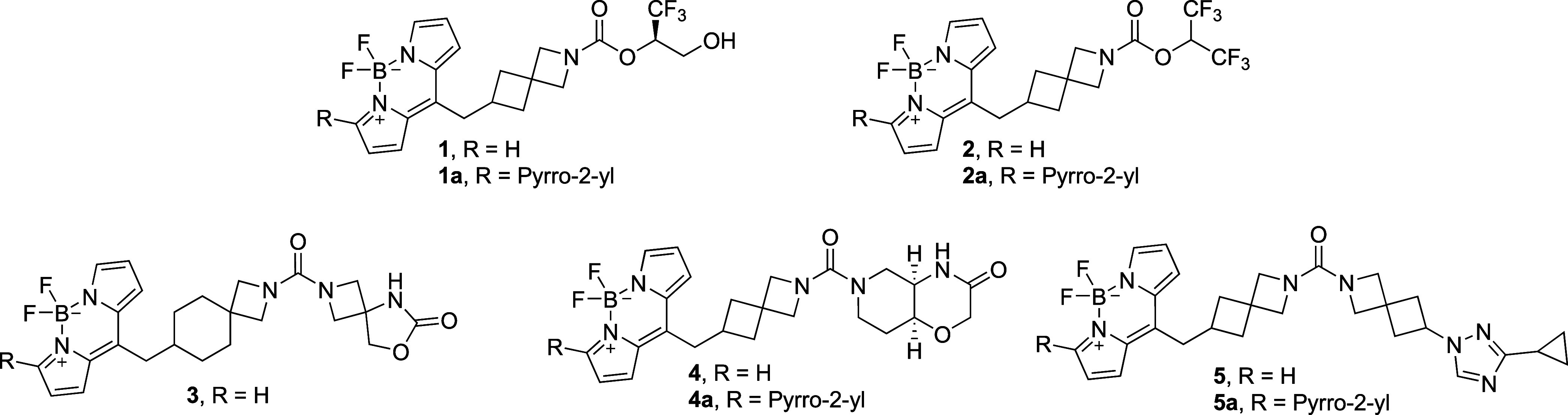
Overview of the Most Active Miniaturized Fluorescent Probes

Previously published^42−46^ structure–activity relationship (SAR) studies of MAGL inhibitors and structural data provide insights into the capacity of the flexible lipophilic binding pocket to harbor various apolar structural motifs. We conducted ligand superimposition and docking studies, which revealed that replacing the aromatic pharmacophore structures in MAGL inhibitors with a fluorescent BODIPY moiety could retain the key interactions toward MAGL. This led to the construction of structures with BODIPY connected to the cyclic amine moieties as a versatile linchpin for the development of reversible and irreversible MAGL probes (Schemes 2 and 3). We combined this with several privileged noncovalent amphiphilic headgroups^43,47−49^ that bind to the glycerol-binding subpocket. Additionally, for covalent MAGL inhibitors, we used reactive carbamate structures with fluorinated alcohols as leaving groups that targeted catalytic Ser122.^27,50^ This approach is a modification of the reverse design principle that uses high-quality drug ligands or components thereof in the development of chemical tool compounds.^51^

In order to achieve the desired BODIPY fluorescent probes, we established a comprehensive modular strategy for the synthesis of 8-alkylene connected constructs. This strategy employs a Liebeskind–Srogl cross-coupling (LSCC)^52^ between vinyl boronic acids and 8-thiomethyl-BODIPY building blocks as the central step (Scheme 2). The synthesis commenced with the construction of vinyl boronic acids obtained via Boron-Wittig olefination to form the corresponding vinyl boronates.^53,54^ They were connected to their respective headgroups after *N*-Boc-deprotection via urea, amide, or carbamate formation. This was followed by the deprotection (if applicable) and reduction of the vinylic double bond under mild conditions. Interestingly, the presence of the conjugated alkene moiety caused a negligible quantum yield of the BODIPY unit while exhibiting high MAGL inhibitory potencies (SI, Table S1: **S11**, **S16**, **S21**).^52^ Hydrogenation of the double bond using triethylsilane and Pd/C in methanol was crucial for liberating the bright fluorescent properties of the BODIPY unit. Compounds **1** and **1a** required an additional PMB-deprotection step (Scheme 2B), which was achieved using DDQ in DCM/H_2_O. Direct C–H arylation at the 3-position of the 8-thiomethyl BODIPY building block was achieved using in situ-generated benzene diazonium. Functionalization at the 3-position with a pyrrol-2-yl moiety led to a strong bathochromic shift of the fluorophore. The desired building block was obtained in one step by microwave-assisted oxidative nucleophilic substitution with neat pyrrole.^55^

Restraining conformational flexibility to the active conformation by spirocyclic moieties was found to be an effective strategy to enhance affinity (see SI, Table S1, **S1** vs **S5**–**S6**)^56,57^ and led to the discovery of the [3.3]-spirocyclic BODIPY motif in **1**. The modular construction design allowed for successive variations of the headgroup by several other structural distinct moieties (Scheme 2C).

We found that the replacement of the headgroup interacting with the amphiphilic subpocket of the active site of MAGL occurs in a modular and independent manner (Scheme 2C). This phenomenon is attributed to the stabilization of the binding conformation by the central carbonyl forming a bond with the oxyanion hole of the serine hydrolase.^45^ The most potent and promising compounds (Scheme 3) underwent comprehensive characterization, encompassing biochemical activity, selectivity, cellular potency, drug-likeness, and physicochemical properties (Table 1), as well as photophysical properties (SI Table S4). All compounds depicted in Scheme 3 exhibited subnanomolar inhibition of human MAGL (hMAGL) in a RapidFire mass spectrometric (MS) assay measuring the hydrolysis of the native 2-AG substrate.

Interestingly, substitution of the BODIPY unit at the 3-position by phenyl (SI, Table S1: **S22**) or pyrrol-2-yl (**1a**, **2a**, **4a**, **5a**) moieties was well-tolerated, with at least on-par inhibitory potency.

Large species-dependent activity differences of MAGL inhibitors are often hampering the translation of preclinical data to clinical development. For instance, the widely used MAGL tool compound, JZL184, showed severely reduced potency on rat MAGL, complicating investigations in rodents.^12,61^ Therefore, we tested our probes’ inhibitory activity against human, mouse, rat, and cynomolgus monkey MAGL orthologs to examine their applicability in translational research using the RapidFire MS native substrate assay monitoring the direct conversion of the endogenous substrate 2-AG.^26^ As illustrated in Table 1, all probes exhibited consistently high potency against MAGL across all tested species. Only **3**, **4**, and **5a** showed minor (less than 4-fold) losses against rat or cynomolgus monkey orthologs. To assess cellular target engagement we performed a nanoluciferase (Nluc)-based bioluminescence resonance energy transfer (nanoBRET) assay using live HEK293 cells.^63^ Here, **5** showed the best cellular potency, with an IC_50_ of 85 nM, while **1**–**4** showed activity in the 200–700 nM range, indicating a high affinity for MAGL and sufficient permeability. The observed variations in cellular target occupancy (i.e., between **2** and **5**) could potentially originate from the complex interplay of additional factors, such as cellular permeability, (serum)protein binding, and membrane accumulation of the probe. Consistent with these results, all inhibitors meet Lipinski’s rule of five^39^ and the “Veber rules”^40^ and showed favorable tPSA and solubility (Table 1). In addition, the choice of the headgroup and substitution of the BODIPY fluorophore significantly influenced these physicochemical properties. For reference, the only published selective MAGL fluorescent labeling probe, **LEI-463**, significantly exceeded these descriptor boundaries (MW = 1087 g/mol, clogP = 9.28, HBA/HBD = 6/1, tPSA = 108 Å^2^, and nRotB = 22). The probes’ physicochemical descriptors, computed lipophilicities, and solubilities are similar to highly optimized MAGL inhibitor drug candidates (SI, Figure S2 and Table S2).

We confirmed both, the reversibility of the urea-containing compounds **4** and **5** through a surface plasmon resonance (SPR) experiment (SI, Figure S13), as well as the covalency of probe **1** through mass spectrometry analysis of labeled MAGL protein (SI, Figure S14). The residence time of **4** and **5** on hMAGL was determined as 37 and 44 min, respectively.

### Co-Crystal Structure and Binding Mode of the Miniaturized Fluorescent Probe **5**

**Figure 1:**
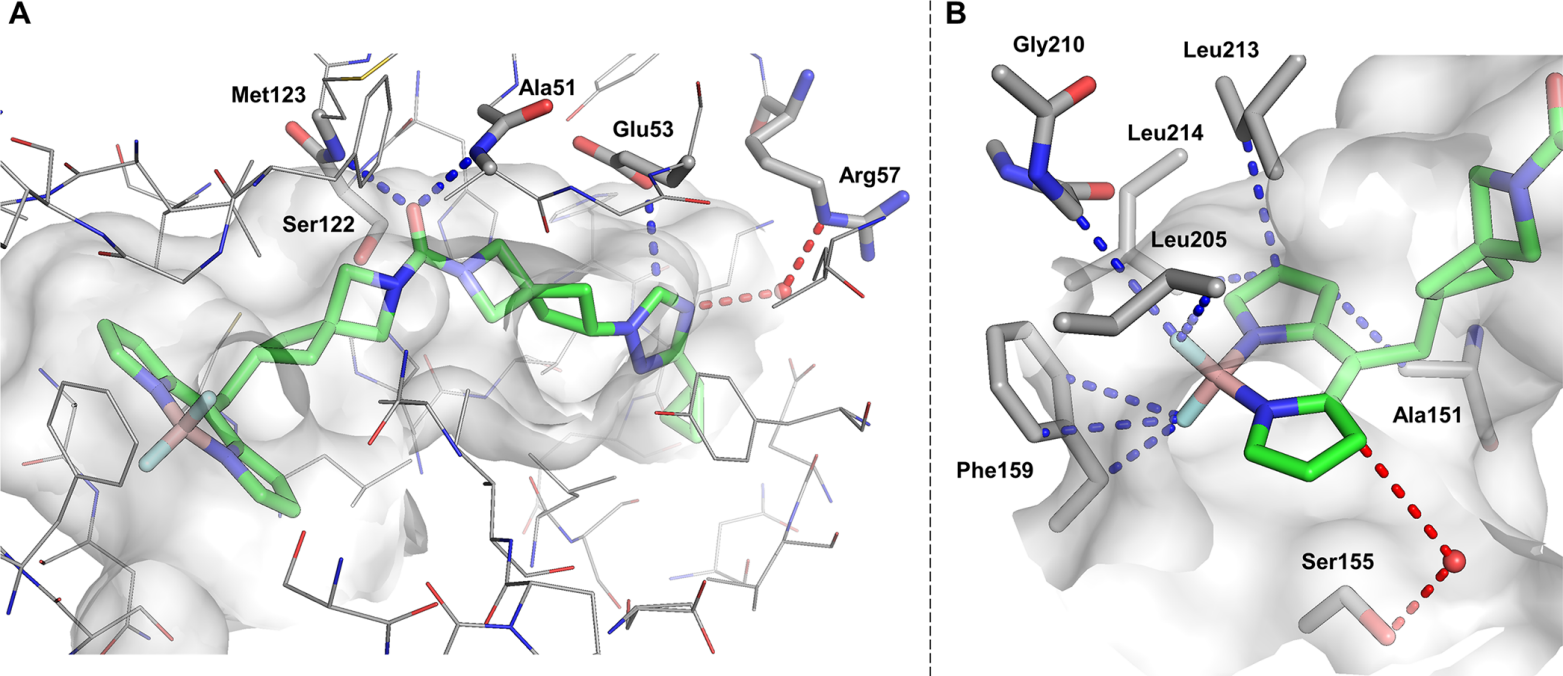
Co-crystal structure of miniaturized fluorescent probe **5** in complex with hMAGL (PDB: 8RVF, resolution = 1.43 Å). (A) Binding mode of compound **5** in hMAGL with water-mediated and direct protein–ligand hydrogen bond interactions indicated by dashed lines (blue for direct binding, red for water-mediated interactions). Key residues for hydrogen bonding, including the oxyanion hole (Ala51, Met123) and catalytic Ser122, are highlighted in stick representation. Additional water molecules have been removed for clarity of view. (B) Close-up view of the boron-dipyrromethene (BODIPY) environment buried in the hMAGL-binding site with indicated key interactions. Short nonbonded contacts (*d* ≤ 4.0 Å), either in direct contact with MAGL residues (blue) or mediated by water (red), are shown by dashed lines.

The co-crystal structure of compound **5** with hMAGL (PDB: 8RVF; Figure 1 and SI, Table S3) revealed the binding mode of the miniaturized fluorescent inhibitors. As hypothesized, the binding mode of **5** in the orthosteric binding pocket was very similar to known nonfluorescent MAGL inhibitors, i.e., compound **13**(26) or SAR629^62^ (PDB: 7PRM, 3JWE; SI, Figures S5 and S6) recapitulating key interactions. The urea carbonyl moiety of **5** bound to the oxyanion hole (Ala51, Met123), with an amphiphilic headgroup forming directed polar interactions (Glu53 and Arg57). The co-crystal structures provided clear evidence that the BODIPY moiety was accommodated within the lipophilic pocket, thereby significantly enhancing the binding affinity through several beneficial lipophilic interactions with the residues of Leu205, Gly210, Leu213, and Leu214 (Figure 1A). Furthermore, an important interaction was observed between the fluorine atom of BODIPY and Phe159, as illustrated in Figure 1B. The latter was remarkable, as one could suspect repulsion between the highly electronegative fluorine and the π-electron system. However, this did not appear to have a significant detrimental effect. Further analysis revealed that the 3- and 4-positions of the BODIPY moiety pointed toward the entrance of the binding pocket, enabling further fluorophore-modulating substitution.

### Photophysical Properties

Red-shifting probe fluorescence to longer wavelengths is highly desirable in cellular- and tissue-level experiments to minimize interference from biological autofluorescence and tissue absorption.^63^ Therefore, we intended to induce a bathochromic shift by 3-substitution of the BODIPY fluorophore without significantly disrupting the protein–ligand interaction pattern.^38,64^ In particular, the pyrrol-2-yl-substituted BODIPY should be able to induce a strong bathochromic shift and may also further enable super-resolution microscopy.^65^ Therefore, this substitution was investigated in combination with the most promising amphiphilic headgroups (**1a**, **2a**, **4a**, **5a**). The obtained 3-pyrrolyl-substituted BODIPYs had almost similar affinity for MAGL but showed a significant spectral red-shift compared to their unsubstituted BODIPY congeners **1**, **2**, **4** and **5**. We observed an emission redshift of 86 nm (594 vs 502 nm) and an increased Stokes shift (24 nm vs 13 nm) for all 3-pyrrolyl-substituted BODIPY derivatives (SI, Table S4 and Figure S15). Although the substituted structures did not achieve the high brightness and quantum yield of symmetric BODIPYs (Φ = 2.0–5.7 vs 56.6–87.8%), we observed adequate imaging capabilities for cellular imaging applications in live cells (SI, Figures S7 and S8). Both, the symmetric BODIPY analogs (**1**, **2**, **3**, **4**, **5**) and the 3-pyrrolyl-substituted probes (**1a**, **2a**, **4a**, **5a**) could be used with readily available filter sets, such as fluorescein-isothiocyanate (FITC) and Cyanine3 (Cy3), respectively.

We further investigated whether solvent characteristics and other solute components would detrimentally influence the fluorescence characteristics of probe **1**. We did not observe significant changes in fluorescence intensity at different pH, by varying polarity or viscosity of the solvent, nor by the presence of common ions and substrates (SI, Figure S16), which is in agreement with reported BODIPY literature.^38^ In addition, probe **1** showed high photostability (SI, Figure S17).

### Determination of Probe Specificity and Selectivity by Activity-Based Protein Profiling (ABPP)

Specificity and selectivity are paramount criteria for any molecular probe,^66−68^ especially given the abundance of a multitude of structurally and functionally similar serine hydrolases present in cells, such as ABHD6, ABHD12, FAAH, and DAGL, which are closely related congeners. We investigated this potential interference by studying the inhibition of more than 60 related hydrolases in a multiplex ABPP assay in the mouse brain proteome (MBP) using brain homogenates, which, in particular, cover all relevant eCS hydrolases.^69^

**Figure 2:**
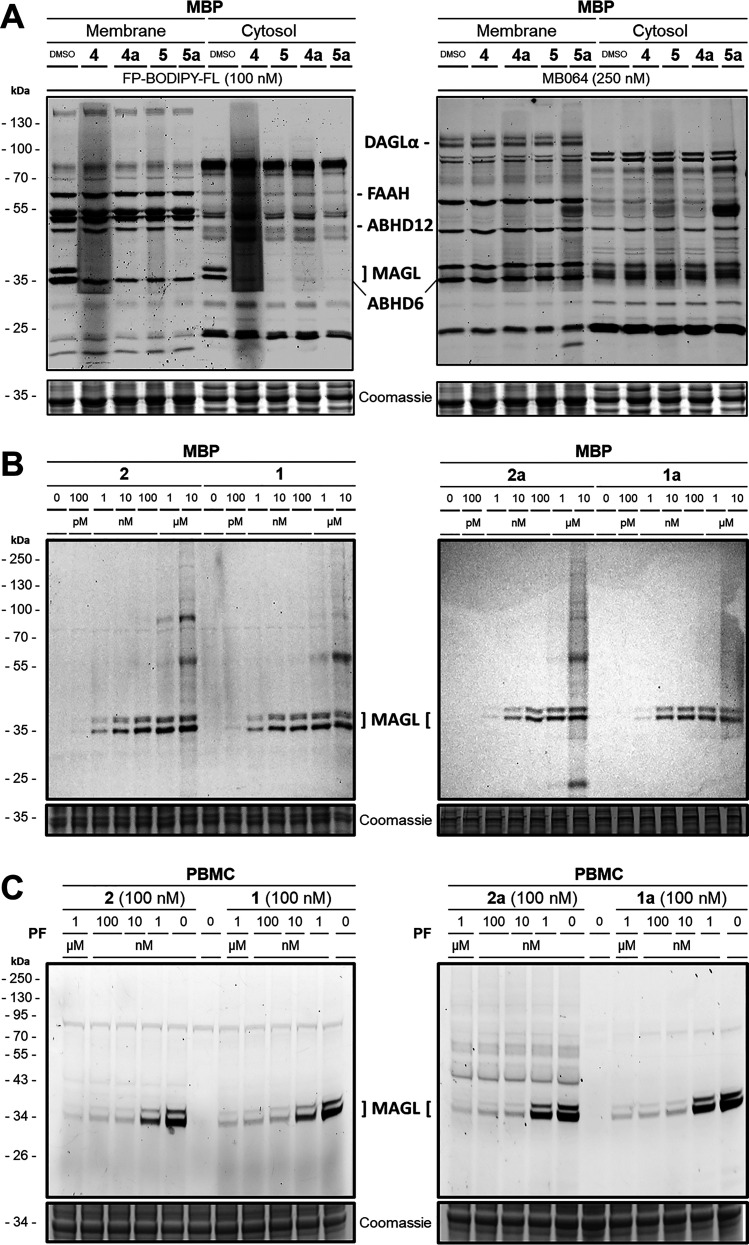
(A) Multiplex activity-based protein profiling (ABPP) assay was used to label various serine hydrolases in the membrane and cytosolic fraction of the mouse brain proteome (MBP). Two MAGL bands at approximately 35 kDa were selectively blocked by 1 μM of the respective reversible probes **4**, **4a**, **5**, **5a**. Both panels depict the same representative SDS-PAGE at the respective fluorescent channel settings. (B) Dose-dependent labeling of the two MAGL splicing variants in MBP with the four irreversible probes **2**, **1**, **2a**, **1a**. (C) Irreversible probes as MAGL-selective ABPP probes for target-occupancy assessment in patient-derived human peripheral blood monocytes (PBMCs) at 100 nM. The MAGL fluorescence signal was dose-dependent to MAGL inhibitor **PF**. Coomassie protein staining as a loading control.

In this assay, two broadband serine hydrolase ABPP probes, FP-BODIPY-FL and MB064,^69^ were utilized to stain the respective enzymes within the membrane and the cytosolic fractions of the mouse brain homogenate, thereby indicating potential inhibitory activity. All tested reversible probes (**4**, **4a**, **5**, **5a**) demonstrated remarkable selectivity, exclusively blocking the two MAGL isoforms present in MBP, with no other potential off-target detectable even at 1 μM concentration (Figure 2A).

Consistent with these findings, we observed for the covalent probes **1**, **1a**, **2**, and **2a** only selective irreversible labeling of the two MAGL isoforms and no labeling of other proteins present in the mouse brain homogenates over a broad range of concentrations (Figure 2B). Minor off-target labeling occurred at very high concentrations of 1 to 10 μM, which was less pronounced for the more sophisticated headgroup in **1** and in the pyrrolyl-substituted analogs **2a** and **1a**. Selective, irreversible fluorescent labeling of the active site Ser122 in such a complex biological sample rendered **1**, **1a**, **2**, and **2a** highly MAGL-selective ABPP probes.

We then investigated whether these probes are able to assess the target occupancy of a MAGL-selective inhibitor PF-06795071 (**PF**) in a clinical ex vivo setting with native, human patient-derived peripheral blood monocytes (PBMCs) (Figure 2C), as PBMCs are an important biomarker system for peripheral inflammation and metabolic diseases.^70^ When applying escalating concentrations of **PF**, we could clearly observe a dose-dependent decrease of the fluorescent signal intensity corresponding to a 40% loss of MAGL activity at a concentration of 1 nM and a 93% loss of MAGL activity after coincubation with 10 nM **PF** in PBMCs.

When screening the probes **1**, **2**, **2a**, **4a**, and **5** against a customized panel of 50 representative unrelated off-target receptors and enzymes, all probes exhibited a very clean selectivity profile at a concentration of 10 μM (>10,000-fold IC_50_; SI, Table S5). This confirms their low propensity for nonspecific binding, even in potentially more complex cellular settings.

Overall, probe **5** emerged as the most preeminent among the evaluated reversible fluorescent MAGL probes. It exhibited picomolar potency across all species orthologs, high cellular activity and selectivity against serine hydrolases, specificity against unrelated off-targets, drug-like characteristics, and good aqueous solubility.

Additionally, the red-shifted analog **5a** retained these favorable features. Correspondingly, the best covalent miniaturized MAGL fluorescent probe was **1** with substituted version **1a**. It outperforms **2** and **2a** in terms of potency, solubility, and selectivity.

### Development of Selective Competition MAGL Binding Assays

**Figure 3:**
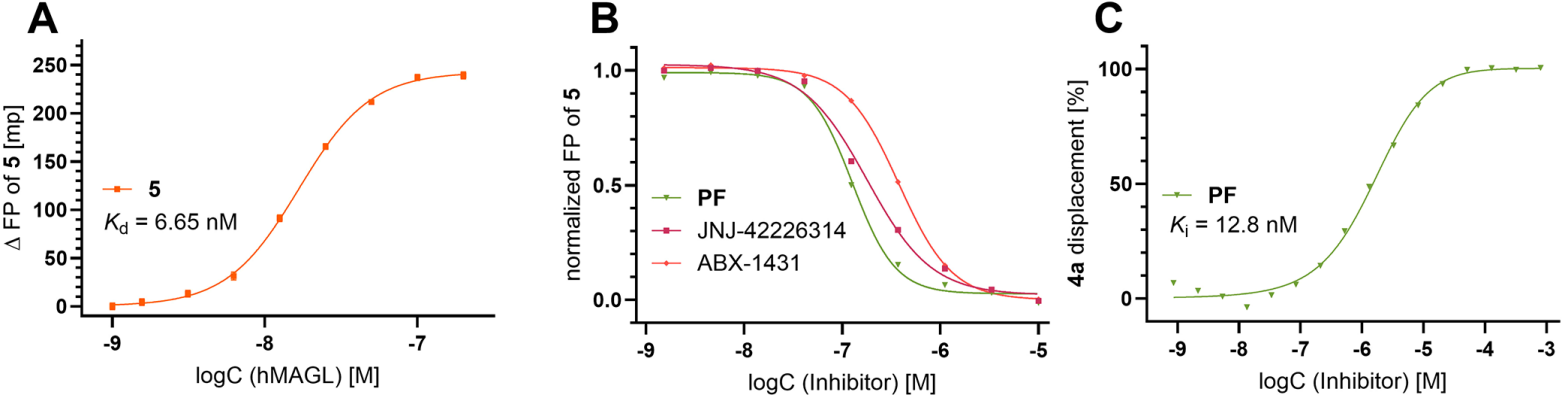
Cell-free fluorescence polarization (FP) and cellular nanoBRET competition assays using the reversible noncovalent fluorescent probes **5** and **4a** as tracers. (A) Fluorescence polarization dose–response titration of reversible probe **5** (20 nM) with purified hMAGL. (B) Three MAGL-specific inhibitors were assessed in a competitive FP assay with **5** (20 nM) and hMAGL (50 nM). (C) Competition nanoBRET assay in live HEK293 cells expressing MAGL-Nluc fusion protein and reversible probe **4a** (100 nM). All experiments were performed at least in triplicate. Mean values are shown.

In contrast to the only currently available MAGL-selective fluorescent covalent probe LEI-463,^28^ probes **3**, **4**, **4a**, **5**, and **5a** are stable, nonreactive and reversible MAGL ligands. Albeit not observed in our investigations, covalent probes may always carry the risk of unspecific reactivity or excessive metabolism.^71^ Additionally, the use of reversible probes instead of irreversible ones allows for various competition binding assays,^72^ and their availability is beneficial for diverse experimental settings.^73−75^ Therefore, we set out to investigate the ability of the reversible BODIPY fluorescent probe **5** to act as a tracer in a fluorescence polarization (FP) binding assay for MAGL. Such a FP-binding assay would be a highly useful addition to the already described functional enzymatic assays and would not require radiolabeled reporters, which makes handling much more convenient. The miniaturized probes have ideal characteristics for FP:^76^ low MW, providing a large difference between bound and unbound polarization, a bright BODIPY fluorophore with an appropriate fluorescence lifetime, and a firm fixation of the fluorophore without rotational freedom within the binding pocket, avoiding depolarization by the so-called “propeller effect”.^76−79^ This resulted in a strong anisotropic effect, with ΔFP_max_ > 350 mp, close to the theoretical maximum.^77^ The *K*_d_ constant for probe **5** was determined at 6.65 nM (Figure 3A), allowing measurement of *K*_i_ values of nonlabeled MAGL inhibitors in a competition assay setting according to the method of Nikolovska-Coleska et al.^80^ (Figure 3B). We investigated three widely used MAGL drug candidates, among them two irreversible inhibitors. For these, it should be noted that all assays are time-dependent, and IC_50_ and *K*_i_ values are not directly comparable between assays: **PF**(50) (*K*_i_ = 12.8 ± 3.9 nM) and ABX-1431^81^ (*K*_i_ = 44.3 ± 2.2 nM), and the most prominent reversible MAGL inhibitor JNJ-42226314^82^ (*K*_i_ = 18.5 ± 3.3 nM), which was in excellent agreement with the reported *K*_*i*_ value of 20 ± 3 nM determined by a fluorogenic assay.^85^ Therefore, this FP-binding assay accurately determines the *K*_i_ values of reversible MAGL inhibitors in a simple standard assay layout (384-well plate, z′ = 0.90) suitable for high-throughput screening (See SI, S48 for experimental details).

In addition to the cell-free FP assay, we exploited the reversible noncovalent binding mechanism of the fluorescent probes in a nanoBRET assay. The pyrrole-substituted BODIPY probe **4a** was used in this assay as a tracer in live HEK293 cells, as the spectral properties of this BODIPY fluorophore are optimally pairing with luciferase as a donor–acceptor pair. Here, **4a** was used without the need for further changes to the standard test conditions, which allowed the determination of the target engagement of MAGL inhibitors in cells. Thereby cellular IC_50_ values of MAGL inhibitors could be assessed. For instance, the cellular IC_50_ of the MAGL inhibitor **PF** was determined to be 1.60 μM (Figure 3C).

### Flow Cytometry and Confocal Imaging in Live Cell Systems Natively Expressing MAGL

**Figure 4:**
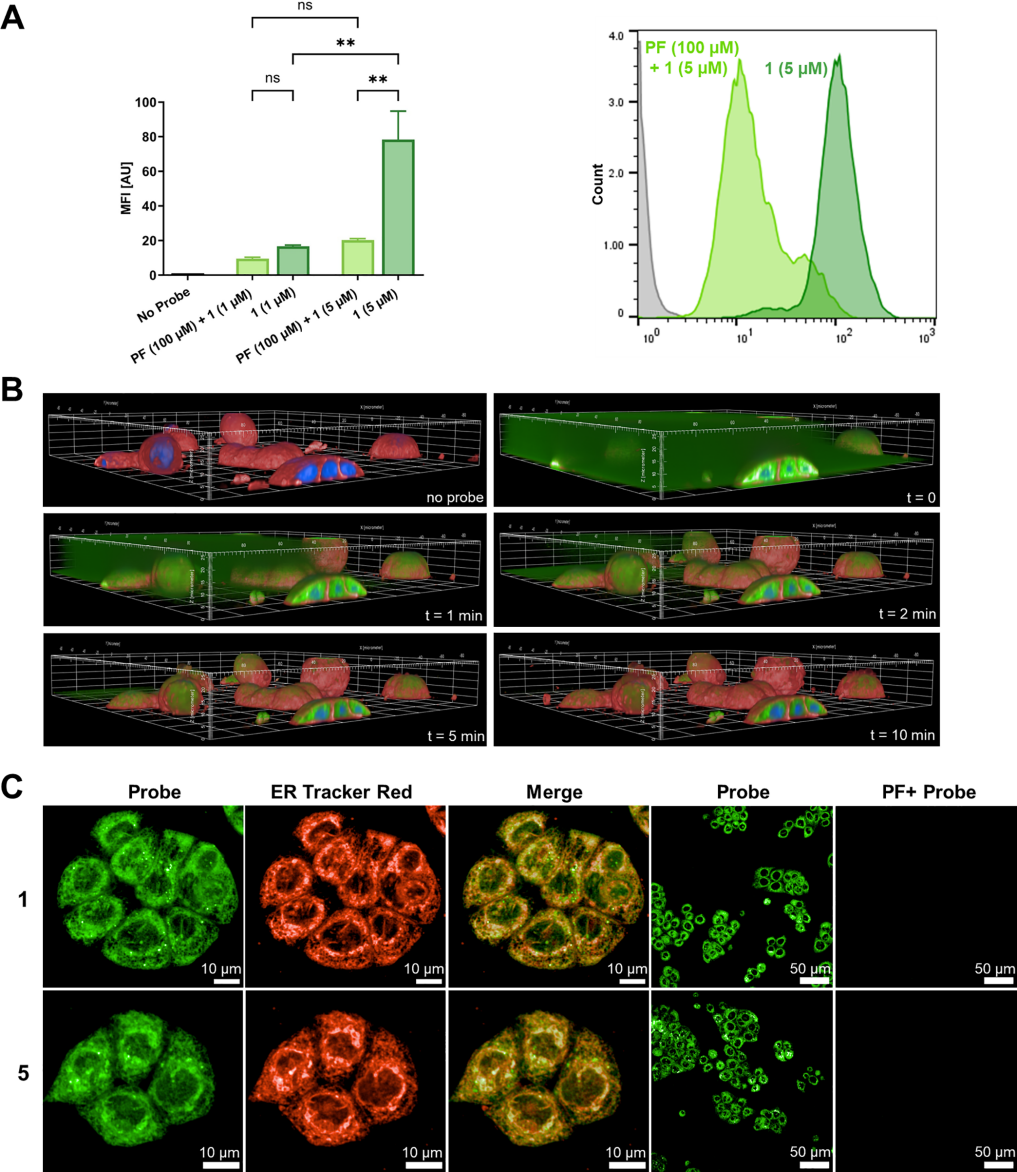
(A) Mean fluorescence intensity (MFI) in the flow cytometry analysis of HT-29 cells with probe **1**. Data was confirmed in two independent experiments, triplicate determination per condition. A one-way ANOVA test was performed. ** indicates *p* < 0.005. Histogram of HT-29 cells: negative control (gray), incubation with **1** (5 μM) for 60 min with preincubation of MAGL inhibitor, **PF** (100 μM; bright green), without blocking (dark green). (B) Uptake process of probe **1** in live HT-29 cells without washing. Three-dimensional confocal microscopic image composite with staining of nuclei (Hoechst; blue), cell membrane (CellMask-A647; Invitrogen; red), and probe **1** (green). Images were acquired using a 63× water immersion objective. Each of the z-stacks consists of 50 confocal planes at a plane distance of 0.5 μm. One field of view is illustrated (200 μm × 200 μm × 25 μm). (C) Confocal live cell microscopy of HT-29 cells revealed the colocalization of MAGL probes **1** and 5 (150 nM, FITC channel) and endoplasmic reticulum ER-tracker (ER-Tracker Red; Invitrogen, Cy3 channel). Preincubation with **PF** (10 μM) blocks the fluorescence signal.

Flow cytometry is a technique widely used in molecular biology, pathology, and physiological disciplines to analyze cell populations. It relies on the specific fluorescent labeling of target protein structures or processes. This can be challenging for intracellular proteins that are not expressed on surfaces. Markers may either not reach the protein target or accumulate nonspecifically in the cell. To investigate whether natively expressed MAGL could be detected by our probe, we incubated human colorectal adenocarcinoma HT-29 cells with various concentrations of covalent probe **1** and analyzed the staining via flow cytometry (Figure 4A). This analysis using probe **1** allowed a clear detection of MAGL-positive HT-29 cancer cells at an elevated concentration of 5 μM. This fluorescent signal was significantly blocked by preincubation with the irreversible MAGL-selective inhibitor **PF**, confirming the specificity of probe **1** in flow cytometry using live cells. The elevated probe concentrations compared to the following imaging applications were necessary to obtain an optimal MAGL-specific signal. This is likely due to unspecific cellular accumulation in this particular flow cytometry setting, as we observed no significantly reduced signal at 1 μM concentration. However, to the best of our knowledge, no specific detection of MAGL by cell flow cytometry for live, native MAGL-positive cells using small-molecule probes has been reported so far.

Next, we performed confocal imaging experiments to study MAGL localization in HT-29 cells (Figure 4B,C) and in the human non-small-cell lung cancer cell line A549 (SI, Figure S10). Both, the reversible probe **5** and the irreversible probe **1**, were equally well suited for this confocal imaging study. We observed bright and stable MAGL staining in both cell lines already at 150 nM. At this low concentration, no washing steps were required for efficient MAGL visualization. We were able to monitor the uptake of the probe and the staining process occurring within intracellular compartments, within a time frame of seconds to minutes (Figure 4B and SI, Figure S8). This observation demonstrates the permeability of the probes utilized in this study. The probe’s MAGL-specificity was confirmed by comparing cellular fluorescence intensities between cells with and without preincubation of MAGL-selective inhibitor **PF** (Figure 4C and SI, Figure S9). We could detect significant localization of MAGL in the endoplasmatic reticulum (ER) in HT-29 cells by costaining experiments using **1** or **5** and ER-Tracker red (Invitrogen) (Figure 4C; Pearson correlation coefficient *r* = 0.98 and 0.87, respectively). Simultaneously, lysosomal or mitochondrial trackers did not indicate MAGL localization in these compartments (SI, Figure S10). This is in accordance with the results of previous studies.^23,27^ Interestingly, besides ER staining, we also observed dense MAGL localization in small, spherical structures, which are likely to be lipid droplets (LD). LDs have been reported to play a crucial role in cancer metabolism, and MAGL is one of its canonical enzymes.^83,84^

**Figure 5:**
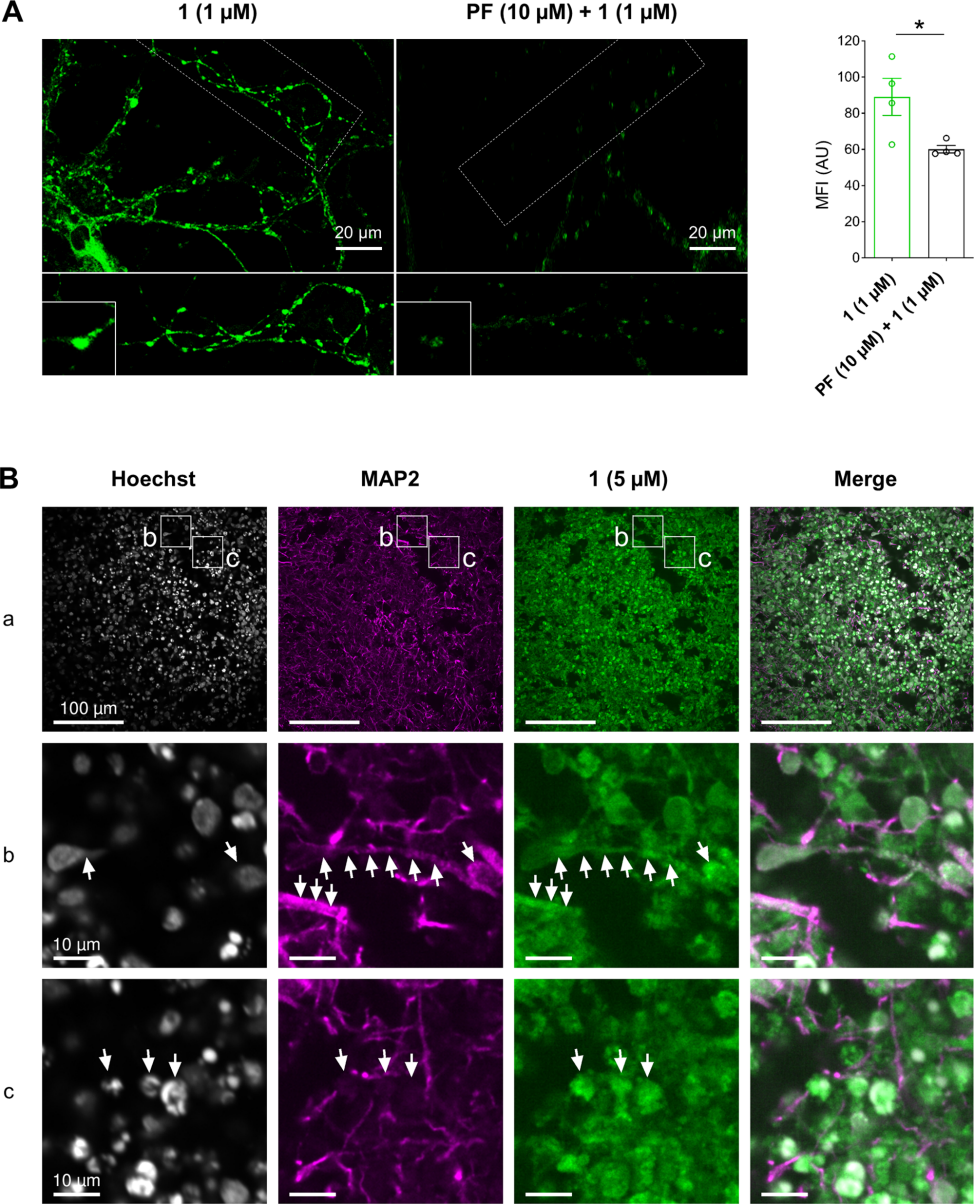
Confocal imaging of primary neuronal cultures and human induced pluripotent stem cell (hiPSC)-derived brain organoids using covalent probe **1**. (A) Left: confocal images of the live dissociated primary hippocampal neuron culture at day-in vitro 14–21. Cells were treated with **1** (1 μM) at 37 °C for 15 min; negative controls were treated with 10 μM MAGL inhibitor **PF** for 2 h prior to MAGL labeling. Neuronal processes (dashed line) and potential synapses (insets) are highlighted; right: quantification of mean fluorescence intensity (MFI) of the signal of **1** in neurons with or without 10 μM **PF** pretreatment (open circles represent *n* = 4 images per condition, two-tailed Student *t* test). (B) Representative confocal images of human induced pluripotent stem cell (hiPSC)-derived brain organoids incubated with 5 μM probe **1** for 2 h after 83 d of organoid maturation, subsequent fixation, cryosectioning, and staining of nuclei (Hoechst) and neurons (MAP2). Subpanel row (a): overview with an indication of regions for subpanels rows (b, c). Arrows indicate several MAP2-positive neurons (subpanel b) and non-neuronal cells, MAP2-negative; subpanel (c) with signal for probe **1**.

Subsequently, we explored the capability of our probes to specifically label MAGL in more complex biological systems. We applied the covalent probe **1** to live primary mouse hippocampal neuron cultures (Figure 5A) and observed a stable and robust MAGL signal in neuronal processes. A significant loss of the mean fluorescence signal after preincubation with the **PF** inhibitor confirmed the high target specificity of probe **1** labeling. A strong fluorescent MAGL signal was also observed in primary astrocytes (SI, Figure S11). The irreversible probes, especially compound **1**, proved to be very robust and suitable for imaging applications in more complex tissue samples that required extensive washing steps.

After demonstrating the use of probe **1** in cultured primary cell lines, we further investigated the probes in human-induced pluripotent stem cell (hiPSC)-derived brain organoids. The probe efficiently permeated the three-dimensional structure of the organoid and detected MAGL in MAP2-positive neurons and MAP2-negative non-neuronal cells in the organoid (Figure 5B). The specificity of **1** was confirmed via preincubation with **PF**, essentially blocking the labeling of MAGL by probe **1** (SI, Figure S12).

### Accessing a Bimodal Fluorescent PET Probe

**Figure 6:**
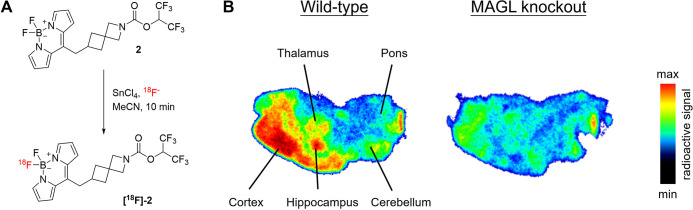
(A) Synthetic conversion of probe **2** into a bimodal fluorescent positron emission tomography (PET) probe **[**^**18**^**F]-2**. (B) In vitro autoradiography of wild-type and MAGL knockout mouse brain sections with **[**^**18**^**F]-2** (45 nM, 8 GBq/μmol).

Fluorescent probes are unparalleled in their ability to provide high spatiotemporal resolution for imaging biological processes. However, they are significantly constrained by the limited depth of tissue penetration of the fluorescence signal.^85^ Radiolabeled ligands are an alternative that can be used for noninvasive imaging modalities, such as positron emission tomography (PET). Direct radiolabeling of fluorescent probe **2** via Lewis acid-mediated isotope exchange with ^18^F at the BODIPY moiety allowed access to a bimodal fluorescent PET imaging probe **[**^**18**^**F]-2** (Figure 6A and SI, Figures S3 and S4).^86^ This probe was then used in an in vitro autoradiography study to image the tissue distribution of MAGL in brain slices of wild-type (WT) and MAGL knockout (KO) mice. Here, 10 μm thin brain slices were incubated for 30 min with a 45 nM solution of the bimodal probe with a molar activity of 8 GBq/μmol. We observed a marked heterogeneous distribution pattern with distinct MAGL-rich brain regions in wild-type mouse brain slices, whereas the radioactive signal was negligible in MAGL knockout mouse brain slices (Figure 6).^26^

**[**^**18**^**F]-2** showed particularly high radioactive accumulation in the hippocampus and cortex of WT mice, consistent with previous MAGL PET tracer studies.^1,24−26^ Moreover, the severely reduced signal intensity in MAGL knockout mouse brain further shows the high MAGL specificity and illustrates the translational versatility of this probe type.

## Conclusions

In this study, we developed a series of versatile, miniaturized MAGL fluorescent probes by merging a bright fluorophore reporter unit with a drug-derived ligand structure. Our approach yielded highly potent, specific, and drug-like probes suitable for various translational experiments. This allowed the specific detection of MAGL in cell or tissue lysate via SDS-PAGE in-gel fluorescence and in live cells via flow cytometry. These probes enabled the establishment of high-throughput FP MAGL-binding assays to stain and localize MAGL in live native cell lines, hippocampal neuron cultures, and hiPSC-derived brain organoids. Here, one fluorescent probe was converted into a bimodal fluorescent [^18^F]-PET probe that specifically and selectively labeled MAGL in brain tissues.

The modular design strategy facilitated the efficient construction of noncovalent and covalent drug-like MAGL probes. Despite the structural restrictions of BODIPY fluorophores, the probe properties were flexibly altered to further extend their applicability in complex biological settings. This miniaturization approach can be used to develop new fluorescent probes that may efficiently cross the blood–brain barrier and be used for visualization applications in the central nervous system in in vivo models. We believe that the miniaturized probe chemotype developed in this study has potential applicability to other protein targets, thereby facilitating the construction of efficient drug-like fluorescent probes for translational validation of protein targets.

## Supporting Information Available

Complete SAR structures; MAGL inhibitory data; experimental details; compound synthesis; analytical characterization; and fluorescence microscopic images (PDF)

## Abbreviations

2-AG: 2-arachidonylglycerol
AA: arachidonic acid
ABHD6/ABHD12: α/β hydrolase domain containing 6/12 serine hydrolases
BODIPY: boron dipyrromethene
*c* log *P*: computed partition coefficient (octanol/water)
DAGL: diacylglycerol lipase
eCS: endocannabinoid system
ER: endoplasmic reticulum
FAAH: fatty-acid amide hydrolase
FACS: fluorescence-activated cell sorting
FP: fluorescence polarization
GFP: green fluorescent protein
HBA/HBD: hydrogen bond acceptor/donor
IC_50_: half-maximal inhibitory concentration
KO: knockout
LiTMP: lithium tetramethylpiperidide
LSCC: Liebeskind–Srogl cross-coupling
MAGL: monoacylglycerol lipase
MAP2: microtubule-associated protein 2 (a neuronal marker)
MBP: mouse brain proteome
MFI: mean fluorescence intensity
mp: milli polarization
MS: mass spectroscopy
MW: molecular weight *or* microwave
PET: positron emission tomography
PF: PF-06795071 MAGL inhibitor
PMB: para-methoxybenzyl
nRotB: number of rotatable bonds
SAR: structure–activity relationship
SDS-PAGE: sodium dodecyl sulfate-polyacrylamide gel electrophoresis
TFP: tri(2-furyl)phosphine
tPSA: topological polar surface area
WT: wild-type
