# Supplementary material for: Highly Specific Miniaturized Fluorescent Monoacylglycerol Lipase Probes Enable Translational Research
Source: J Am Chem Soc. 2025 Mar 10;147(12):10188–202. doi: 10.1021/jacs.4c15223 (PMC11951083; doi:10.1021/jacs.4c15223)
Supplement: Supplementary file 1 — ja4c15223_si_001.pdf [file ja4c15223_si_001.pdf]

Supporting Information

Highly Specific Miniaturized Fluorescent Monoacylglycerol Lipase Probes Enable Translational Research

Axel Hentsch[#], Mónica Guberman[#], Silke Radetzki[#], Sofia Kaushik[#], Mirjam Huizenga[‖], Yingfang He[∇], Jörg Contzen[†‡], Bernd Kuhn[§], Jörg Benz[§], Maria Schippers[§], Jerome Paul[#], Lea Leibrock[§], Ludovic Collin[§], Matthias Wittwer[§], Andreas Topp[§], Fionn O'Hara[§], Dominik Heer[§], Remo Hochstrasser[§], Julie Blaising[§], Jens Peter von Kries[#], Linjing Mu[∇], Mario van der Stelt[‖], Philipp Mergenthaler [†‡⊥], Noa Lipstein[#], Uwe Grether[§], and Marc Nazaré*[#]

Leibniz Forschungsinstitut für Molekulare Pharmakologie, Campus Berlin-Buch, 13125 Berlin (Germany)

‖ Division of Drug Discovery and Safety, Leiden Academic Centre for Drug Research, Leiden University, 2333 CC, Leiden (The Netherlands)

† Charité - Universitätsmedizin Berlin, Center for Stroke Research 10117 Berlin (Germany)

‡ Charité - Universitätsmedizin Berlin, Dept. of Neurology with Experimental Neurology, 10117 Berlin (Germany)

⊥ University of Oxford, Radcliffe Department of Medicine, OX3 9DU Oxford (United Kingdom)

∇ ETH Zürich, Institute of Pharmaceutical Sciences, Vladimir-Prelog-Weg 4, 8093 Zürich (Switzerland)

§ Roche Pharma Research & Early Development, 4070 Basel (Switzerland)

Table of Contents

Structure-activity relationship (SAR): Structures and IC_{50} values of MAGL-BODIPY Fluorescent Probes S3

Experimental Details - Synthesis S7

General experimental procedures S8

Compound Synthesis and Characterization S11

HPLC Chromatograms S40

Experimental Details S42

Microscopic Images S54

Reversibility / Covalency Behavior S57

Photo-physical properties S59

CEREP Data S62

References S64

Structure-Activity Relationship (SAR): Structures and IC_{50} values of MAGL-BODIPY Fluorescent Probes

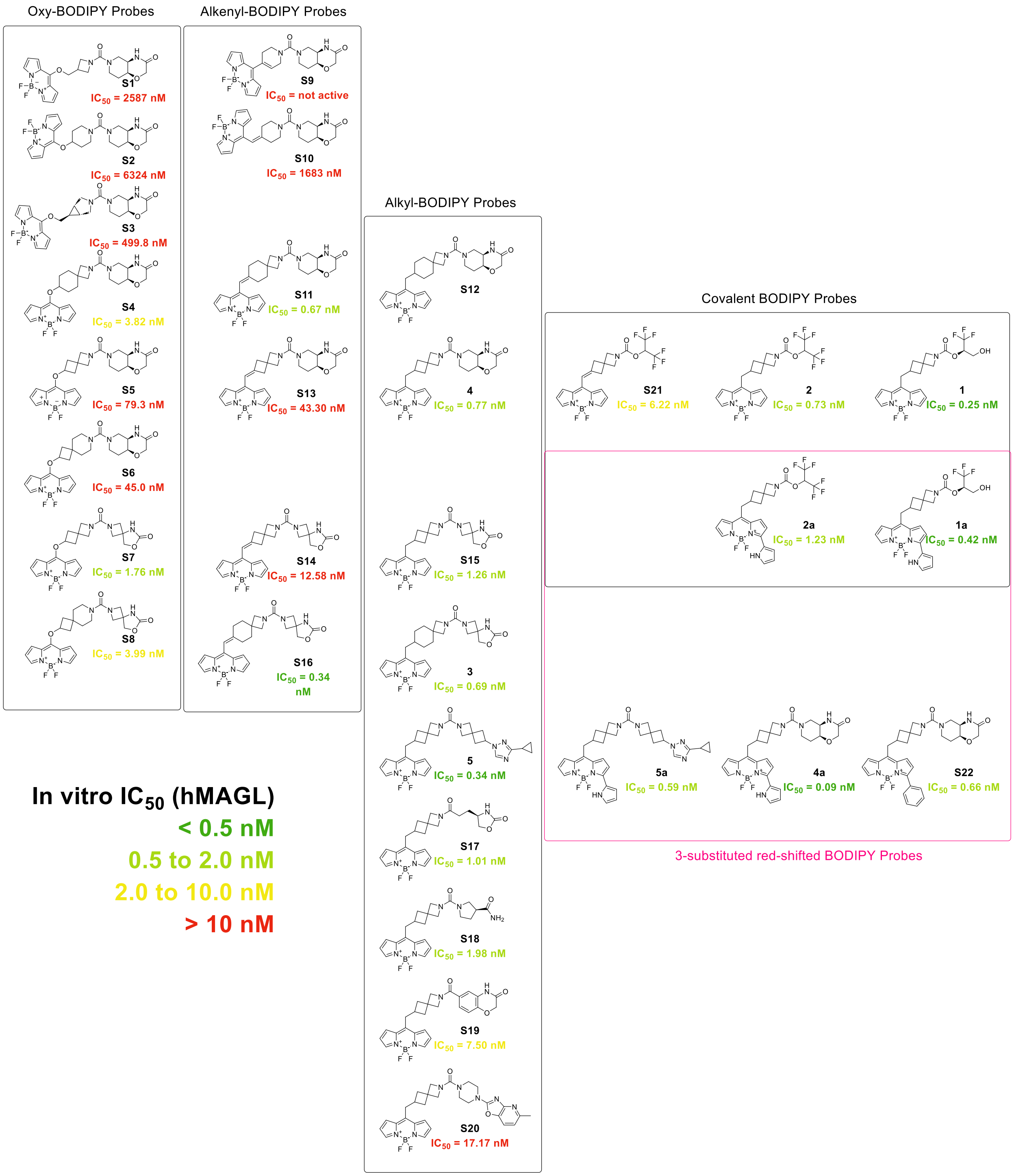

Figure S1. *Complete SAR of the synthesized structures with color-coded IC_{50} values against human MAGL.*

SI Table 1. *IC_{50} values of all assessed MAGL inhibitory probes. SEM is indicated if the assay was repeated three or more times. Data from native mass spectrometry (MS) substrate assay of purified human (h), mouse (m), rat (r), and cynomolgus monkey (c) MAGL enzymes in vitro; note that IC_{50} values of covalent inhibitors (**1**, **1a**, **2**, and **2a**) are in general time-dependent. Data of cellular nano-bioluminescence resonance energy transfer (nanoBRET) assay in live HEK293 cells.*

	**IC_{50} against purified MAGL ± SEM [nM]**				**NanoBRET IC_{50} ± SEM [nM]**
	Human	Mouse	Rat	Cynomolgus	HEK293
**1**	0.28 ± 0.04	0.21± 0.03	0.36 ± 0.05	0.87 ± 0.18	276 ± 43
**1a**	0.47 ± 0.11	0.31± 0.06	0.44 ± 0.02	0.40 ± 0.05	n.a.
**2**	0.39 ± 0.12	0.33 ± 0.07	0.20 ± 0.08	0.24 ± 0.07	720 ± 270
**2a**	0.98 ± 0.14	0.79 ± 0.14	0.68 ± 0.07	0.63 ± 0.09	n.a.
**3**	0.83 ± 0.14	0.96 ± 0.28	3.54 ± 0.37	3.86 ± 0.44	557
**4**	0.94 ± 0.12	1.33 ± 0.15	2.40 ± 0.61	1.14 ± 0.20	196 ± 40
**4a**	0.14 ± 0.02	0.18 ± 0.03	0.47 ± 0.03	0.20 ± 0.01	n.a.
**5**	0.58 ± 0.24	1.03 ± 0.44	0.98	0.50	85 ± 87
**5a**	0.73 ± 0.13	0.90 ± 0.12	2.23 ± 0.12	0.77 ± 0.06	n.a.
**S1**	2587.40	>10000	-	-	-
**S2**	6324.00	>10000	-	-	-
**S3**	499.85	1042.12	-	-	>10000
**S4**	3.82 ± 1.91	7.06 ± 4.73	21.57	8.37	4632 ± 52
**S5**	79.30	734.70	-	-	-
**S6**	45.02	170.40	-	-	>10000
**S7**	1.76	4.71	-	-	>10000
**S8**	3.99	13.60	-	-	>10000
**S9**	>10000	>10000	-	-	-
**S10**	1683.16	3396.25	-	-	>10000
**S11**	0.64	1.85	-	-	314
**S12**	2.35	12.43	10.92	2.73	564
**S13**	43.30	182.47	-	-	>10000
**S14**	12.58	12.41	-	-	>10000
**S15**	1.26	1.36	4.46	3.27	5544
**S16**	0.67	0.81	-	-	574
**S17**	1.01	0.84	-	-	300
**S18**	1.98	2.07	-	-	1944
**S19**	7.50	18.60	-	-	299
**S20**	17.17	108.30	-	-	>10000
**S21**	6.22	9.41	-	-	>10000
**S22**	0.66	0.37	-	-	56 ± 6

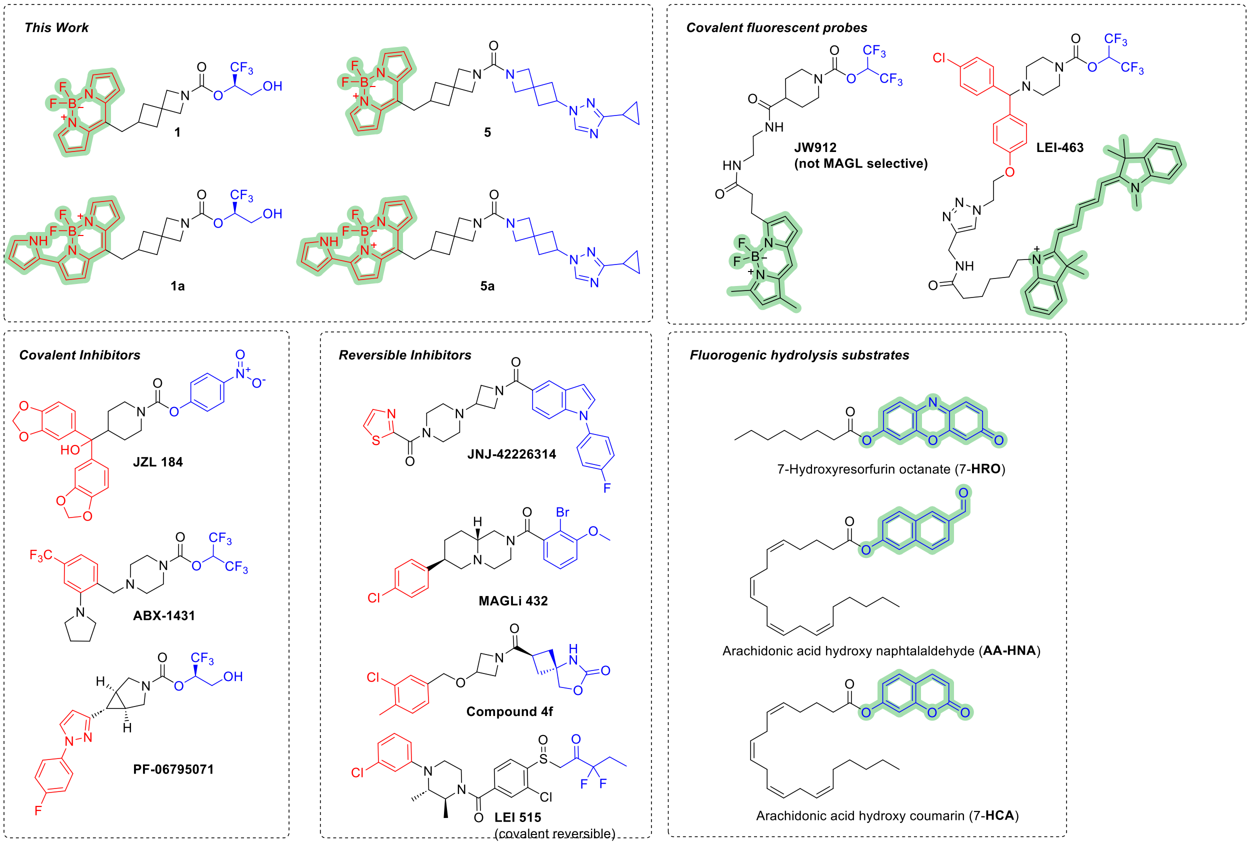

Figure S2. *Chemical structures of selected small molecule MAGL probes, classified by their mode of action. Headgroup (blue), aromatic pharmacophore (red), and fluorophore (green highlight) are indicated.*

Table S2. *IC_{50} values of the selected small molecular MAGL probes. Calculated physicochemical descriptors for an estimation of drug-like behavior. n.r. = not reported; n.a. = not applicable. MW, clogP, tPSA, nRotB, logS, and Lipinski Ro5 or Veber-rule fulfillment were calculated via SwissADME software. References as indicated.*

Compound	Reference	Mode of Action	hMAGL IC_{50} [nM]	MW [*g/mol*]	clogP	HBA/ HBD	tPSA [Å^2]	*nRotB*	logS	Ro5 conform	Veber conform
1	This work	covalent fluorescent probe	0.28	457	2.06	3/1	63	7	-4.2	Yes	Yes
1a	This work	covalent fluorescent probe	0.47	522	2.54	3/2	79	8	-4.9	Yes	Yes
5	This work	reversible fluorescent probe	0.58	531	1.99	3/0	68	4	-4.7	Yes	Yes
5a	This work	reversible fluorescent probe	0.73	596	3.40	3/1	70	5	-5.2	Yes	Yes
LEI-463	(1)	covalent fluorescent probe	ca. 10	1087	9.28	6/1	108	22	-12.2	No	No
JW-912	(2)	unselective fluorescent probe	n.r.	639	2.92	4/2	101	14	-5.4	Yes	No
7-HCA	(3)	unselective fluorogenic substrate	n.a.	449	2.92	4/0	57	16	-5.4	Yes	No
7-HRO	(4)	unselective fluorogenic substrate	n.a.	339	3.94	5/0	69	8	-4.5	Yes	Yes
AA-HNA	(5)	unselective fluorogenic substrate	n.a.	459	3.94	3/0	43	17	-4.5	Yes	Yes
PF-06795071	(6)	covalent inhibitor	3	399	2.9	5/1	68	7	-3.7	Yes	Yes
ABX-1431	(7)	covalent inhibitor	14	507	5	6/0	36	9	-5.8	Yes	Yes
MAGLi 432	(8)	reversible inhibitor	4.2	464	4.27	3/0	33	4	-5.8	Yes	Yes
JNJ-42226314	(9)	reversible inhibitor	1.13	490	3.21	5/0	90	6	-5.0	Yes	Yes
JZL-184	(10)	covalent inhibitor	8	520	3.04	9/1	132	7	-5.6	No	Yes
LEI-515	(11)	reversible covalent inhibitor	0.5	531	4.81	5/0	77	8	-6.4	Yes	Yes
Compound 4f	(12)	reversible inhibitor	6.2	365	2.12	4/1	68	5	-3.0	Yes	Yes

Experimental Details - Synthesis

Chemicals and anhydrous solvents were purchased from commercial suppliers (Sigma-Aldrich, BLDPharm, Abcr, Carbolution, VWR, TCI) and used as received unless otherwise specified.

(4aR,8aS)-3-oxooctahydro-2*H*-pyrido[4,3-b][1,4]oxazin-6-ium 2,3-bis((4-methylbenzoyl)oxy)succinate,[13]
7-oxa-2,5-diazaspiro[3.4]octan-6-one tosylate,[14]
6-(3-cyclopropyl-1*H*-1,2,4-triazol-1-yl)-2-azaspiro[3.3]heptan-2-ium triflate,[15]
(*R*)-3-(2-oxooxazolidin-4-yl)propanoic acid,[16]
3-oxo-3,4-dihydro-2*H*-benzo[b][1,4]oxazine-6-carboxylic acid,
and 5-methyl-2-(piperazin-1-yl)oxazolo[4,5-b]pyridine[17]
were kindly received from F. Hoffmann La-Roche AG's research and early development department.

TLC was carried out on aluminum-backed silica gel plates (silica gel 60 F 254, Merck), visualizing with UV light (λ = 254 nm). Checked for fluorescence at λ = 365 nm, if applicable.

LC-MS was performed with an Agilent 1260 series HPLC system employing a DAD detector (at 600, 500, 300, 254 and 220 nm) equipped with Agilent Technologies 6120 Quadrupole LC/MS in electrospray positive mode (ESI+). A Thermo Accuore RP-MS (30 × 2.1 mm, 2.6 µm) column was used with a flow rate 0.8 mL/min in combination with the following separation conditions: 0.1% formic acid in water (solvent A); 0.1% formic acid in ACN (solvent B); 5% B for 0.2 min, from 5 to 95% B in 0.9 min, 95% B for 1.4 min (stop point at 2.5 min). Data analysis was performed with ChemStation software.

Purification via flash chromatography was carried out using Biotage Isolera One apparatus or Combiflash NextGen 300+ apparatus equipped with ELSD detector using RediSep®Rf columns from Teledyne Isco. Preparative HPLC was performed on a Gilson PLC 2250 with a Macherey-Nagel VP 250/21 Nucleodur 100-7 C18Ec column (30 mL/min flow) or a Macherey-Nagel VP 250/10 Nucleodur 100-5 C18Ec column (5 mL/min flow).

HRMS analyses were carried out on Agilent Technologies 6530 Accurate Mass Q-ToF LC/MS linked to Agilent Technologies HPLC 1260 Infinity II.

NMR spectra were recorded at either Bruker AV 300 (295K, 300 MHz, 75 MHz) or Bruker AV 600 (300K, 600 MHz, 151 MHz) spectrometers in $CDCl_3$, CD_3CN, and CD_3OD as solvents. All ^{13}C NMR-spectra were recorded with ^{1}H-broad-band decoupling. All chemical shifts are reported in ppm (δ) relative to the respective solvent signal (δ = ^{1}H: 7.26, 1.94, 3.31 ppm and ^{13}C: 77.16, 1.32, 49.00 ppm, respectively). NMR data were analyzed with MestReNova 14.2.1 software.

Compound names were generated with ChemDraw 20.1 software and are not necessarily identical to IUPAC nomenclature.

Compound purity was ensured by ^{1}H-NMR, ^{13}C-NMR, and HPLC measurement and exceeded 95% purity for all tested compounds.

Safety Statement: No unexpected or unusually high safety hazards were encountered.

General experimental procedures

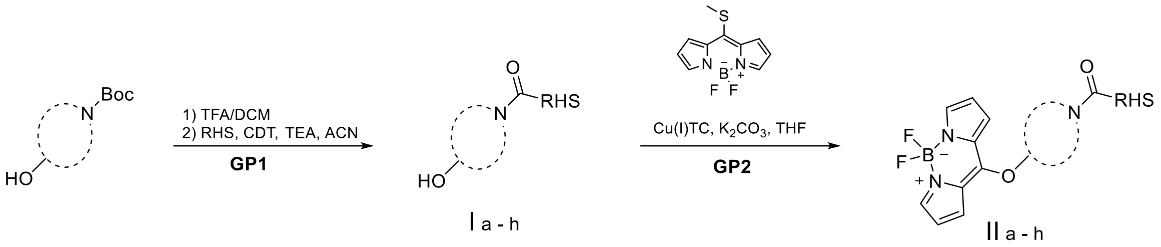

SI Scheme 1. *Reaction scheme for the synthesis of 8-oxy-BODIPY MAGL probes (II a-h).*

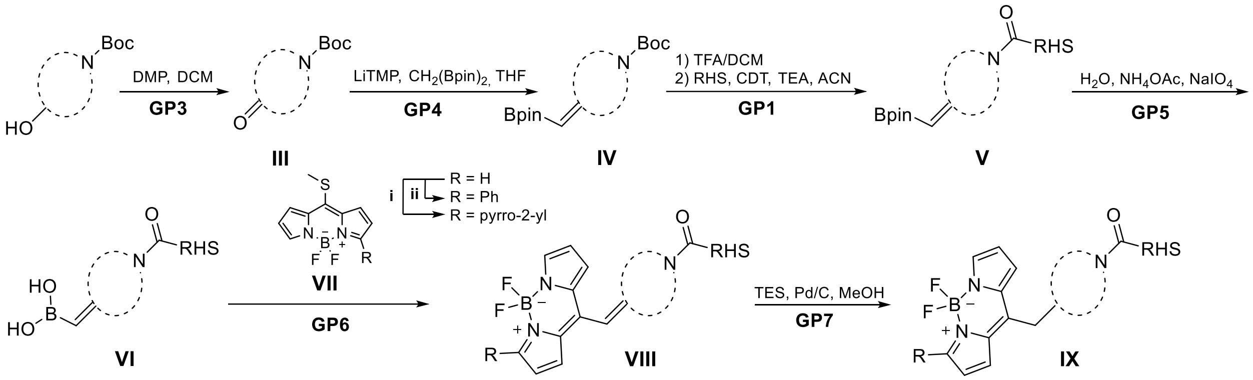

Scheme S2. *Reaction scheme for the synthesis of 8-alkylene-BODIPY MAGL probes (IXa-r).*

GP1: Urea Coupling

Method A (CDT-Coupling)

The *N*-Boc-protected left-hand-side secondary amine (1.0 equiv.) was deprotected by stirring in 20% TFA in DCM for 2-4 hours. The solution was co-evaporated with toluene twice to give the crude secondary amine TFA salt. The right-hand-side secondary amine was dissolved in a mixture of anhydrous ACN and TEA (7.0 equiv.), then CDT (1.0 equiv.) was added, and the solution was stirred at rt. for 2 hours. The deprotected amine-TFA salt was dissolved in a small quantity of ACN and added dropwise to the reaction mixture before heating to 50 °C for 1 to 3 hours until LC-MS indicates completion of the reaction. The mixture was diluted with EtOAc, and washed with a saturated $NaHCO_3$ solution and saturated NaCl solution. The organic layer was dried over Na_2SO_4, filtrated, and concentrated in vacuo. The product was purified by either silica gel chromatography or preparative RP-HPLC.

Method B (Triphosgene-Coupling)

The *N*-Boc-protected left-hand-side secondary amine (1.0 equiv.) was deprotected by stirring in 20% TFA in DCM for 4h. The solution was co-evaporated with toluene twice to give the crude secondary amine TFA salt. The residue was dissolved in 1 mL anhydrous DCM and added dropwise at 0 °C to a suspension of triphosgene (0.8 equiv.) and $NaHCO_3$ (4.0 equiv.) in DCM under a nitrogen atmosphere. The mixture was stirred overnight at ambient temperature. The mixture was filtrated through a PTFE syringe filter (45 µm) and directly added dropwise at 0 °C to a solution of the right-hand-side secondary amine salt (1.0 equiv.) and DIPEA (4.0 equiv.) in 1 mL DCM. The mixture was allowed to warm to r.t., and stirring was continued overnight. The mixture was then concentrated under

reduced pressure and separated between saturated $NaHCO_3$ solution and EtOAc. The aqueous layer was extracted two additional times with EtOAc. The combined organic layer was dried over $MgSO_4$, filtrated, and concentrated. The crude was purified either via silica gel chromatography or preparative RP-HPLC

GP2: Alcohol BODIPY conjugation

The respective alcohol (1.0 equiv.) and 8-(methylthio)-4,4,difluoro-4-bora-3a,4a-diaza-s-indacene (1.0 equiv.) were dissolved in anhydrous ACN. The mixture was purged with nitrogen for 5 minutes, then copper(I)thiophene-2-carboxylate (1.0 equiv.) was added. After an additional 5 min of purging, sodium carbonate (1.0 equiv.) was added. The mixture was lowered into a preheated oil bath at 55 °C for 18 h to 48 h until LC-MS indicated the consumption of the alcohol starting material. The mixture was cooled to rt. and carefully concentrated under reduced pressure. The residue is taken up in ACN:H_2O 1:1, filtrated through a PTFE filter (45 µm), and purified via RP-HPLC with a gradient elution of 15% to 85% of ACN in H_2O (+0.1 % TFA).

GP3: Dess-Martin-Oxidation

The secondary alcohol was dissolved in anhydrous DCM (0.1 M) and cooled to 0 °C. Dess-Martin periodinane (1.2 equiv.) was added, and the mixture was allowed to warm to ambient temperature and stirred for 2 h. The mixture was diluted with DCM, and a concentrated solution of sodium thiosulfate was added to quench excess DMP. The biphasic mixture was vigorously stirred for 30 min before the layers were separated, and the organic layer was extracted with saturated $NaHCO_3$ solution and saturated NaCl solution. The organic phase was dried over $MgSO_4$, filtrated, and concentrated under reduced pressure. The products were purified by silica gel flash chromatography with a gradient elution of 1% to 10% MeOH in DCM.

GP4: Boro-Wittig Reaction

An oven-dried flask was charged with 2,2,6,6-tetramethylpiperidine (2.0 equiv.) dissolved in THF (0.1 M) and cooled to -78 °C under a nitrogen atmosphere. n-Butyllithium solution (2.5M in hexanes, 2.0 equiv.) was added dropwise, and the reaction mixture was stirred at the same temperature for 30 min. Next, a solution of bis(4,4,5,5-tetramethyl-1,3,2-dioxaborolan-2-yl)methane (2.0 equiv.) in THF (0.4 M) was added dropwise. The reaction was allowed to stir for 5 minutes. Then the respective ketone (1.0 equiv.) in THF (0.1 M) was added dropwise over 5 min. The reaction was slowly allowed to warm to r.t. overnight. Upon completion, the reaction was opened to air, filtered through a silica plug, and flushed with diethyl ether. The mixture was concentrated under reduced pressure and adsorbed onto silica to be purified by silica gel chromatography with gradient elution from 0% to 20% EtOAc in cyclohexane (ELS detection recommended).

GP5: Bpin hydrolysis

The boronic acid pinacol ester (1.0 equiv.), sodium periodate (5.0 equiv.), and ammonium acetate (5.0 equiv.) were dissolved in acetone/water 2:1 (0.05 M boronate) and stirred for 18 to 24 h at ambient temperature until LC-MS indicated consumption of the starting material. Acetone was removed under reduced pressure, and 4 mL of ACN/H_2O 1:1 were added, and the mixture was filtrated. The filtrate was purified via RP-HPLC 5% to 75% ACN:H_2O (+ 0.1% TFA) to give the products as colorless solids.

GP6: Liebeskind-Srogl BODIPY C-C Crosscoupling

The respective boronic acid (1.0 equiv.) and 8-thiomethyl-BODIPY derivative (1.0 equiv.) were dissolved in anhydrous THF. The mixture was purged with nitrogen for 10 minutes. Then copper(I)thiophene-2-carboxylate (3.0 equiv.) was added, followed by tris(dibenzylideneacetone)-dipalladium(0) (7.5 mol%) and tri-(2-furyl)-phosphine (22.5 mol%). The mixture was placed into a preheated oil bath at 55 °C for 20 to 120 minutes until LC-MS indicated the consumption of the boronic acid. The mixture was cooled and then concentrated under reduced pressure. The residue is taken up in ACN:H_2O 1:1 (or 4:1 for more apolar analogs), filtrated through a PTFE filter (45 µm), and purified via RP-HPLC with a gradient elution of 15% to 85% of ACN in H_2O (+0.1 % TFA).

GP7: Hydrogenation of double bond

The alkenyl-BODIPY compound was dissolved in anhydrous methanol, and Pd/C (20 mol%) was added. Triethylsilane (20 equiv.) was added dropwise at 0 °C. The reaction was allowed to warm to r.t., and after completion (controlled by LC-MS, 30 to 90 min), a few drops of water were added, and Pd/C was removed by filtration through a PTFE filter (22 µm). The solution was concentrated under reduced pressure and purified by RP-HPLC to give the alkyl-BODIPY derivatives.

Compound Synthesis and Characterization

8-Oxy-BODIPY MAGL probes

(4aR,8aS)-6-(3-(Hydroxymethyl)azetidine-1-carbonyl)hexahydro-2*H*-pyrido[4,3-b][1,4]oxazin-3(4H)-one (Ia)

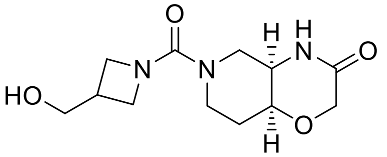

The title compound was synthesized according to GP1 A from tert-butyl 3-(hydroxymethyl)azetidine-1-carboxylate (15 mg, 0.08 mmol) and (4aR,8aS)-3-oxooctahydro-2*H*-pyrido[4,3-b][1,4]oxazin-6-ium 2,3-bis((4-methylbenzoyl)oxy)succinate (59.5 mg, 0.08 mmol) as a colorless amorphous solid (12 mg, 56%). LC-MS (ESI): m/z = 270.1 $[M+H]^+$

^{1}H NMR (300 MHz, CD_3CN) δ 6.68 (s, 1H), 4.10 (d, *J* = 4.3 Hz, 2H), 3.95 (dt, *J* = 10.3, 8.2 Hz, 3H), 3.81 – 3.62 (m, 3H), 3.59 (d, *J* = 6.3 Hz, 2H), 3.57 – 3.49 (m, 1H), 3.34 – 3.25 (m, 1H), 3.00 – 2.82 (m, 2H), 2.75 – 2.58 (m, 1H), 1.88 – 1.69 (m, 2H).

(4aR,8aS)-6-(3-(((5,5-Difluoro-5*H*-4λ⁴,5λ⁴-dipyrrolo[1,2-c:2',1'-f][1,3,2]diazaborinin-10-yl)oxy)methyl)azetidine-1-carbonyl)hexahydro-2*H*-pyrido[4,3-b][1,4]oxazin-3(4H)-one (S1 IIa)

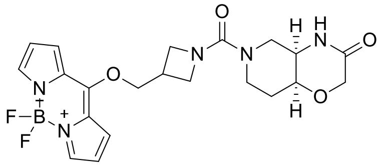

The title compound was synthesized according to GP2 from **Ia** (8.1 mg, 30 µmol) as an amorphous orange solid (2.4 mg, 17%). LC-MS (ESI): m/z = 440.1 $[M-F]^+$

^{1}H NMR (600 MHz, CD_3CN) δ 7.72 (s, 2H), 7.51 (d, J = 4.3 Hz, 2H), 6.62 – 6.58 (m, 2H), 4.88 (d, J = 4.9 Hz, 2H), 4.21 – 4.05 (m, 4H), 4.02 (dd, J = 8.6, 5.5 Hz, 1H), 3.98 (dd, J = 8.6, 5.5 Hz, 1H), 3.95 (q, J = 3.2 Hz, 1H), 3.82 (ddt, J = 13.1, 2.9, 1.6 Hz, 1H), 3.57 (ddt, J = 13.4, 4.6, 2.2 Hz, 1H), 3.31 (dtd, J = 11.6, 4.7, 2.6 Hz, 1H), 3.15 (tdd, J = 8.4, 6.8, 4.2 Hz, 1H), 3.00 – 2.92 (m, 2H), 1.88 – 1.74 (m, 3H).

^{13}C NMR (151 MHz, CD_3CN) δ 167.69, 162.07, 138.86, 126.19, 125.73, 116.43, 75.81, 69.28, 67.15, 52.77, 52.74, 49.22, 45.95, 39.34, 29.18, 28.86.

(4aR,8aS)-6-(4-Hydroxypiperidine-1-carbonyl)hexahydro-2*H*-pyrido[4,3-b][1,4]oxazin-3(4H)-one (Ib)

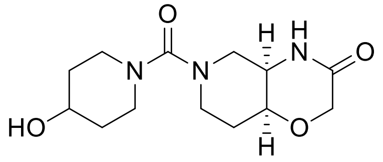

The title compound was synthesized according to GP1 A from tert-butyl 4-hydroxypiperidine-1-carboxylate (15 mg, 0.08 mmol) and (4aR,8aS)-3-oxooctahydro-2*H*-pyrido[4,3-b][1,4]oxazin-6-ium 2,3-bis((4-methylbenzoyl)oxy)succinate (59.5 mg, 0.08 mmol) as a colorless amorphous solid (12 mg, 56%).

LC-MS (ESI): m/z = 284.1 $[M+H]^+$

^{1}H NMR (300 MHz, MeOD) δ 4.49 – 4.38 (m, 2H), 4.37 – 4.16 (m, 6H), 4.09 (q, *J* = 3.1 Hz, 2H), 3.68 – 3.55 (m, 2H), 3.31 (dt, *J* = 3.3, 1.6 Hz, 1H), 3.29 – 2.92 (m, 2H), 2.16 – 1.99 (m, 4H).

(4aR,8aS)-6-(4-((5,5-Difluoro-5*H*-4λ^4,5λ^4-dipyrrolo[1,2-c:2',1'-f][1,3,2]diazaborinin-10-yl)oxy)piperidine-1-carbonyl)hexahydro-2*H*-pyrido[4,3-5 b][1,4]oxazin-3(4H)-one (S2 IIb)

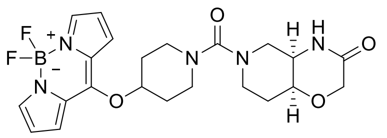

The title compound was synthesized according to GP2 from **Ib** (22.2 mg, 75 µmol) as an amorphous orange solid (11.8 mg, 33%). LC-MS (ESI): m/z = 454.1 [M-F]$^+$

HRMS (ESI) m/z [M+H]$^+$ calcd. for

^{1}H NMR (300 MHz, CD_3CN) δ 7.70 (s, 2H), 7.50 – 7.42 (m, 2H), 6.62 (s, 1H), 6.63 – 6.55 (m, 2H), 5.53 (tt, *J* = 6.9, 3.5 Hz, 1H), 4.11 (d, *J* = 4.3 Hz, 2H), 3.96 (d, *J* = 3.1 Hz, 1H), 3.60 (dd, *J* = 12.8, 4.9 Hz, 1H), 3.45 (ddd, *J* = 15.3, 13.2, 7.3 Hz, 4H), 3.34 – 3.22 (m, 2H), 3.06 – 2.92 (m, 2H), 2.20 – 2.08 (m, 2H), 2.02 (ddt, *J* = 9.9, 6.1, 3.4 Hz, 2H), 1.85 (dt, *J* = 5.9, 3.4 Hz, 2H).

^{13}C NMR (75 MHz, CD_3CN) δ 167.77, 163.27, 138.88, 125.51, 116.57, 80.61, 69.35, 49.11, 47.91, 43.19, 43.12, 41.54, 30.27, 29.02.

(4aR,8aS)-6-((1R,5S,6R)-6-(Hydroxymethyl)-3-azabicyclo[3.1.0]hexane-3-carbonyl)hexahydro-2*H*-pyrido[4,3-b][1,4]oxazin-3(4H)-one (Ic)

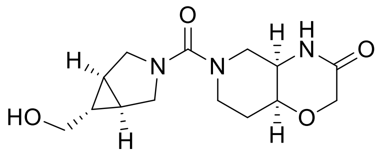

The title compound was synthesized according to GP1 B from tert-butyl (1R,5S,6r)-6-(hydroxymethyl)-3-azabicyclo[3.1.0]hexane-3-carboxylate (priorly synthesized from (1R,5S,6r)-3-(tert-butoxycarbonyl)-3-azabicyclo[3.1.0]hexane-6-carboxylic acid as described in the literature (WO2021030711)) (42.7 mg, 0.2 mmol) and (4aR,8aS)-3-oxooctahydro-2*H*-pyrido[4,3-b][1,4]oxazin-6-ium 2,3-bis((4-methylbenzoyl)oxy)succinate (149 mg, 0.2 mmol) as a colorless amorphous solid (10 mg, 17%). LC-MS (ESI): m/z = 296.2 [M+H]$^+$

^{1}H NMR (300 MHz, MeOD) δ 4.31 – 4.10 (m, 2H), 4.03 – 3.97 (m, 1H), 3.81 – 3.68 (m, 2H), 3.52 – 3.35 (m, 4H), 3.16 – 2.92 (m, 2H), 1.96 – 1.80 (m, 2H), 1.47 – 1.43 (m, 1H), 1.39 – 1.35 (m, 3H), 1.30 (s, 3H).

(4aR,8aS)-6-((1R,5S,6R)-6-(((5,5-Difluoro-5*H*-4λ^4,5λ^4-dipyrrolo[1,2-c:2',1'-f][1,3,2]diazaborinin-10-yl)oxy)methyl)-3-azabicyclo[3.1.0]hexane-3-carbonyl)hexahydro-2*H*-pyrido[4,3-b][1,4]oxazin-3(4H)-one (S3 IIc)

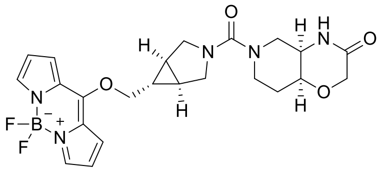

The title compound was synthesized according to GP2 from **Ic** (8.9 mg, 30 µmol) as an amorphous orange solid (2.1 mg, 14%). LC-MS (ESI): m/z = 466.2 [M-F]$^+$

HRMS calc. for $C_{23}H_{26}BF_2N_5O_4$ m/z = 484.2082 found 484.2108

^{1}H NMR (600 MHz, CD_3CN) δ 7.70 (s, 2H), 7.49 (d, *J* = 4.2 Hz, 2H), 6.58 (dd, *J* = 4.3, 2.0 Hz, 3H), 4.68 (d, *J* = 7.3 Hz, 2H), 4.16 – 4.05 (m, 2H), 3.96 (q, *J* = 3.1 Hz, 1H), 3.83 (d, *J* = 10.7 Hz, 1H), 3.75 (d, *J* = 10.7 Hz, 1H), 3.64 – 3.60 (m, 1H), 3.46 – 3.41 (m, 1H), 3.41 – 3.36 (m, 2H), 3.33 (dt, *J* = 10.7, 1.7 Hz, 1H), 2.94 (ddd, *J* = 13.3, 10.6, 5.8 Hz, 2H), 1.84 (dt, *J* = 4.5, 2.2 Hz, 2H), 1.74 – 1.69 (m, 2H), 1.05 (tt, *J* = 7.1, 3.1 Hz, 1H).

^{13}C NMR (151 MHz, CD_3CN) δ 168.67, 162.82, 139.54, 127.26, 126.72, 117.27, 78.26, 70.41, 68.12, 50.43, 50.23, 50.02, 48.50, 42.00, 30.06, 22.25, 22.14, 20.73.

(4aR,8aS)-6-(7-Hydroxy-2-azaspiro[3.5]nonane-2-carbonyl)hexahydro-2Hpyrido[4,3-b][1,4]oxazin-3(4H)-one (Id)

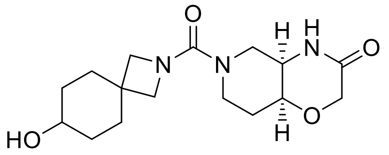

The title compound was synthesized according to GP1 A from tert-butyl 7-hydroxy-2-azaspiro[3.5]nonane-2-carboxylate (48.3 mg mg, 0.2 mmol) and (4aR,8aS)-3-oxooctahydro-2*H*-pyrido[4,3-b][1,4]oxazin-6-ium 2,3-bis((4-methylbenzoyl)oxy)succinate (148.8 mg, 0.2 mmol) as a colorless amorphous solid (37 mg, 57%). LC-MS (ESI): m/z = 324.1 $[M+H]^+$

^{1}H NMR (300 MHz, MeOD) δ 4.28 – 4.10 (m, 2H), 4.00 (d, *J* = 3.1 Hz, 1H), 3.89 – 3.81 (m, 1H), 3.81 – 3.71 (m, 2H), 3.68 (d, *J* = 3.7 Hz, 2H), 3.66 – 3.52 (m, 2H), 3.39 – 3.32 (m, 1H), 3.09 – 2.93 (m, 2H), 1.95 – 1.74 (m, 6H), 1.54 (td, *J* = 12.3, 3.2 Hz, 2H), 1.42 – 1.24 (m, 2H).

^{13}C NMR (75 MHz, MeOD) δ 171.17, 163.88, 70.51, 69.28, 68.22, 62.64, 50.44, 47.19, 40.61, 35.68, 34.34, 32.15, 30.43.

(4aR,8aS)-6-(7-((5,5-Difluoro-5*H*-4λ^4,5λ^4-dipyrrolo[1,2-c:2',1'-f][1,3,2]diazaborinin-10-yl)oxy)-2-azaspiro[3.5]nonane-2-carbonyl)hexahydro-2Hpyrido[4,3-b][1,4]oxazin-3(4H)-one (S4 IId)

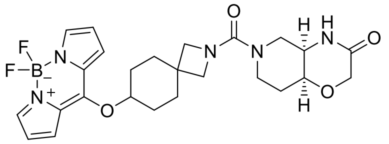

The title compound was synthesized according to GP2 from **Id** (24.3 mg, 75 µmol) as an amorphous orange solid (18.6 mg, 50%).

LC-MS (ESI): m/z = 494.3 $[M-F]^+$ HRMS (ESI) m/z $[M+H]^+$ calcd. for $C_{25}H_{30}BF_2N_5O_4$ 512.2395 found 512.2443

^{1}H NMR (300 MHz, CD_3CN) δ 7.69 (s, 2H), 7.42 (d, *J* = 4.2 Hz, 2H), 6.69 (s, 1H), 6.62 – 6.53 (m, 2H), 5.33 (q, *J* = 3.3 Hz, 1H), 4.22 – 4.03 (m, 2H), 3.96 (d, *J* = 3.1 Hz, 1H), 3.86 – 3.77 (m, 1H), 3.77 – 3.68 (m, 4H), 3.57 (ddd, *J* = 13.2, 4.7, 2.2 Hz, 1H), 3.37 – 3.27 (m, 1H), 2.95 (ddd, *J* = 13.2, 11.3, 7.4 Hz, 2H), 2.10 – 1.99 (m, 3H), 1.93 – 1.71 (m, 6H).

^{13}C NMR (75 MHz, CD_3CN) δ 169.00, 162.99, 161.82, 139.60, 127.53, 126.34, 117.42, 82.78, 70.22, 68.04, 62.40, 61.51, 50.16, 46.88, 40.27, 35.00, 32.63, 30.10, 28.43.

(4aR,8aS)-6-(6-Hydroxy-2-azaspiro[3.3]heptane-2-carbonyl)hexahydro-2*H*-pyrido[4,3-b][1,4]oxazin-3(4H)-one (Ie)

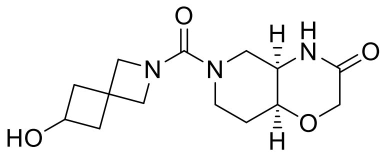

The title compound was synthesized according to GP1 A from tert-butyl 6-hydroxy-2-azaspiro[3.3]heptane-2-carboxylate (42.7 mg, 0.2 mmol) and (4aR,8aS)-3-oxooctahydro-2*H*-pyrido[4,3-b][1,4]oxazin-6-ium 2,3-bis((4-methylbenzoyl)oxy)succinate (148.8 mg, 0.2 mmol) as a colorless amorphous solid (13.5 mg, 23%). LC-MS (ESI): m/z = 296.1 $[M+H]^+$

S5 IIe (4aR,8aS)-6-(6-((5,5-Difluoro-5*H*-4λ^4,5λ^4-dipyrrolo[1,2-c:2',1'-f][1,3,2]diazaborinin-10-yl)oxy)-2-azaspiro[3.3]heptane-2-carbonyl)hexahydro-2*H*-pyrido[4,3-b][1,4]oxazin-3(4H)-one (S5 IIe)

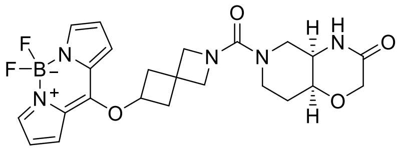

The title compound was synthesized according to GP2 from **Ie** (11.8 mg, 40 µmol) as an amorphous orange solid (6.1 mg, 31%). LC-MS (ESI): m/z = 466.2 [M-F]$^+$.

HRMS (ESI) m/z [M+Na]$^+$ calcd. for $C_{25}H_{30}BF_2N_5O_4Na$ 508.1942 found 508.1962

^{1}H NMR (600 MHz, CD_3CN) δ 7.70 (s, 2H), 7.43 (d, *J* = 4.2 Hz, 2H), 6.62 (s, 1H), 6.61 – 6.54 (m, 2H), 5.48 (tt, *J* = 6.6, 5.1 Hz, 1H), 4.16 – 4.05 (m, 2H), 4.05 – 3.98 (m, 4H), 3.95 (q, *J* = 3.1 Hz, 1H), 3.78 (ddt, *J* = 13.1, 5.1, 1.3 Hz, 1H), 3.52 (ddt, *J* = 13.5, 4.7, 2.1 Hz, 1H), 3.29 (dtd, *J* = 11.6, 4.7, 2.5 Hz, 1H), 2.98 – 2.87 (m, 4H), 2.62 – 2.53 (m, 2H), 1.86 – 1.81 (m, 1H), 1.76 (dddd, *J* = 14.4, 12.6, 5.2, 3.2 Hz, 1H).

^{13}C NMR (151 MHz, CD_3CN) δ 168.80, 162.80, 161.18, 139.90, 127.13, 126.47, 117.53, 76.53, 70.28, 68.13, 63.76, 63.55, 50.19, 46.86, 41.60, 41.58, 40.27, 32.53, 30.12.

If (4aR,8aS)-6-(2-Hydroxy-7-azaspiro[3.5]nonane-7-carbonyl)hexahydro-2*H*-pyrido[4,3-b][1,4]oxazin-3(4H)-one (If)

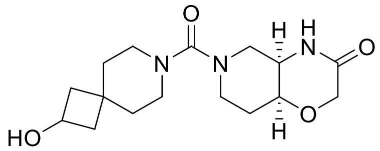

The title compound was synthesized according to GP1 B from tert-butyl 2-hydroxy-7-azaspiro[3.5]nonane-7-carboxylate (48.3 mg, 0.2 mmol) and (4aR,8aS)-3-oxooctahydro-2*H*-pyrido[4,3-b][1,4]oxazin-6-ium 2,3-bis((4-methylbenzoyl)oxy)succinate (148.8 mg, 0.2 mmol) as a colorless amorphous solid (31 mg, 48%). LC-MS (ESI): m/z = 324.2 [M+H]$^+$

^{1}H NMR (300 MHz, MeOD) δ 4.29 – 4.20 (m, 1H), 4.19 (d, *J* = 3.8 Hz, 2H), 4.00 (q, *J* = 3.2 Hz, 1H), 3.67 – 3.59 (m, 1H), 3.49 – 3.32 (m, 2H), 3.25 – 3.12 (m, 4H), 3.11 – 2.95 (m, 2H), 2.26 (ddd, *J* = 11.6, 5.9, 2.2 Hz, 2H), 1.90 (dt, *J* = 5.7, 3.3 Hz, 2H), 1.68 (ddt, *J* = 11.8, 7.5, 3.8 Hz, 2H), 1.57 (dt, *J* = 7.4, 3.4 Hz, 3H), 1.37 (dd, *J* = 6.5, 2.6 Hz, 1H).

^{13}C NMR (75 MHz, MeOD) δ 171.19, 165.51, 70.59, 68.19, 63.36, 50.38, 49.11, 45.33, 45.13, 43.18, 42.87, 40.61, 37.45, 31.36, 30.30.

(4aR,8aS)-6-(2-((5,5-Difluoro-5*H*-4λ^4,5λ^4-dipyrrolo[1,2-c:2',1'-f][1,3,2]diazaborinin-10-yl)oxy)-7-azaspiro[3.5]nonane-7-carbonyl)hexahydro-2*H*-pyrido[4,3-b][1,4]oxazin-3(4H)-one (S6 IIf)

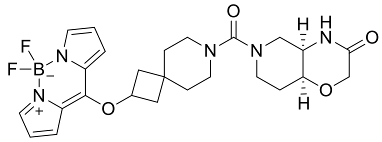

The title compound was synthesized according to GP2 from **If** (16.2 mg, 50 µmol) as an amorphous orange solid (5.7 mg, 22%).

LC-MS (ESI): m/z = 494.2 [M-F]$^+$ HRMS (ESI) m/z [M+H]$^+$ calc. for $C_{25}H_{30}BF_2N_5O_4$ 512.2395 found 512.2423

^{1}H NMR (600 MHz, CD_3CN) δ 7.71 (s, 2H), 7.46 (d, *J* = 4.2 Hz, 2H), 6.74 – 6.69 (m, 1H), 6.63 – 6.58 (m, 2H), 5.59 (tt, *J* = 7.0, 4.7 Hz, 1H), 4.20 – 4.08 (m, 2H), 3.97 (q, *J* = 3.1 Hz, 1H), 3.61 – 3.55 (m, 1H), 3.39 (dddd, *J* = 17.9, 7.4, 6.1, 3.2 Hz, 2H), 3.21 – 3.13 (m, 2H), 2.97 (ddd, *J* = 13.2, 10.8, 5.1 Hz, 2H), 2.65 – 2.60 (m, 2H), 2.24 – 2.20 (m, 2H), 1.87 – 1.83 (m, 2H), 1.71 (dd, *J* = 6.8, 4.5 Hz, 1H), 1.67 (td, *J* = 4.9, 1.6 Hz, 2H).

^{13}C NMR (151 MHz, CD_3CN) δ 169.12, 164.52, 161.31, 139.73, 127.18, 126.45, 117.46, 77.44, 70.34, 68.02, 50.14, 48.92, 44.78, 44.56, 42.58, 40.65, 40.63, 38.93, 37.48, 33.30, 29.97.

2-(6-Hydroxy-2-azaspiro[3.3]heptane-2-carbonyl)-7-oxa-2,5-diazaspiro[3.4]octan-6-one (Ig)

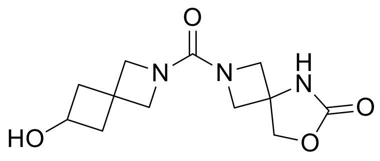

The title compound was synthesized according to GP1 A from tert-butyl 6-hydroxy-2-azaspiro[3.3]heptane-2-carboxylate (42.7 mg, 0.2 mmol) and 7-oxa-2,5-diazaspiro[3.4]octan-6-one tosylate (60.7 mg, 0.2 mmol) as a colorless amorphous solid by precipitation from EtOAc (31 mg, 58%). LC-MS (ESI): m/z = 268.1 $[M+H]^+$

^{1}H NMR (300 MHz, D_2O) δ 4.62 (s, 2H), 4.16 (s, 4H), 4.14 (p, 1H), 3.97 (d, *J* = 11.5 Hz, 4H), 2.51 (ddt, *J* = 9.2, 5.4, 2.8 Hz, 2H), 2.06 (ddd, *J* = 13.0, 6.7, 2.8 Hz, 2H).

^{13}C NMR (75 MHz, D_2O) δ 162.48, 160.40, 74.67, 62.43, 62.22, 61.09, 60.95, 54.59, 48.80, 42.21, 29.64.

2-(6-((5,5-Difluoro-5*H*-4λ^4,5λ^4-dipyrrolo[1,2-c:2',1'-f][1,3,2]diazaborinin-10-yl)oxy)-2-azaspiro[3.3]heptane-2-carbonyl)-7-oxa-2,5-diazaspiro[3.4]octan-6-one (S7 IIg)

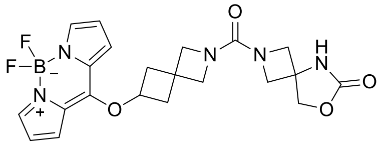

The title compound was synthesized according to GP2 from **Ig** (13.4 mg, 50 µmol) as an amorphous orange solid (3.6 mg, 16%).

LC-MS (ESI): m/z = 438.1 $[M-F]^+$

^{1}H NMR (600 MHz, CD_3CN) δ 7.70 (s, 2H), 7.42 (d, *J* = 4.2 Hz, 2H), 6.58 (dd, *J* = 4.4, 2.0 Hz, 2H), 6.45 (s, 1H), 5.51 – 5.44 (m, 1H), 4.47 (s, 2H), 4.04 – 3.97 (m, 4H), 3.96 (d, *J* = 1.8 Hz, 4H), 2.92 – 2.87 (m, 2H), 2.58 – 2.54 (m, 2H).

^{13}C NMR (151 MHz, CD_3CN) δ 161.16, 158.45, 139.92, 127.12, 126.47, 117.54, 76.50, 75.15, 63.52, 62.59, 62.51, 55.26, 41.50, 32.88.

2-(2-Hydroxy-7-azaspiro[3.5]nonane-7-carbonyl)-7-oxa-2,5-diazaspiro[3.4]octan-6-one (Ih)

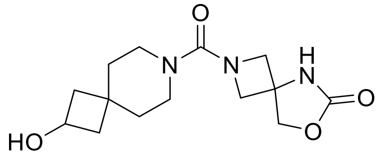

The title compound was synthesized according to GP1 B from tert-butyl 2-hydroxy-7-azaspiro[3.5]nonane-7-carboxylate (48.3 mg, 0.2 mmol) and 7-oxa-2,5-diazaspiro[3.4]octan-6-one tosylate (60.0 mg, 0.2 mmol) as a colorless amorphous solid (8.8 mg, 15%). LC-MS (ESI): m/z = 296.2 $[M+H]^+$

^{1}H NMR (300 MHz, MeOD) δ 4.72 – 4.49 (m, 5H), 4.41 – 4.11 (m, 3H), 4.12 (s, 3H), 3.32 – 3.17 (m, 4H), 2.25 (ddt, *J* = 9.1, 7.5, 2.2 Hz, 2H), 1.75 – 1.61 (m, 2H), 1.53 (dt, *J* = 8.1, 3.1 Hz, 4H).

^{13}C NMR (75 MHz, MeOD) δ 163.73, 160.57, 75.76, 75.04, 65.80, 64.70, 63.32, 62.83, 56.37, 55.55, 43.27, 43.15, 43.05, 40.67, 37.53, 31.24.

2-(2-((5,5-Difluoro-5*H*-4λ^4,5λ^4-dipyrrolo[1,2-c:2',1'-f][1,3,2]diazaborinin-10-yl)oxy)-7-azaspiro[3.5]nonane-7-carbonyl)-7-oxa-2,5-diazaspiro[3.4]octan-6-one (S8 IIh)

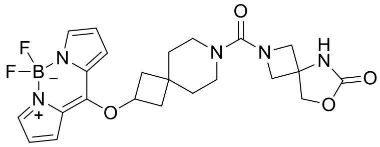

The title compound was synthesized according to GP2 from **Ih** (8.8 mg, 30 µmol) as an amorphous orange solid (3.1 mg, 20%). LC-MS (ESI): m/z = 466.2 $[M-F]^+$ HRMS calc. for $C_{23}H_{26}BF_2N_5O_4$ m/z = 484.2082 found 484.2100

^{1}H NMR (600 MHz, CD_3CN) δ 7.69 (s, 2H), 7.44 (d, *J* = 4.2 Hz, 2H), 6.58 (dd, *J* = 4.2, 2.0 Hz, 2H), 6.41 (s, 1H), 5.57 (tt, *J* = 7.0, 4.7 Hz, 1H), 4.47 (s, 2H), 4.05 (d, *J* = 9.3 Hz, 2H), 4.01 (d, *J* = 9.3 Hz, 2H), 3.26 –

3.23 (m, 2H), 3.22 – 3.19 (m, 2H), 2.62 – 2.57 (m, 2H), 2.22 – 2.17 (m, 2H), 1.66 – 1.63 (m, 2H), 1.62 – 1.59 (m, 2H).

^{13}C NMR (151 MHz, CD_3CN) δ 162.90, 161.29, 158.49, 139.74, 127.17, 126.45, 117.46, 77.41, 75.27, 64.27, 54.93, 42.60, 42.32, 40.58, 38.94, 37.52, 33.21.

8-alkylene-BODIPY MAGL probes

***tert*-Butyl 7-oxo-2-azaspiro[3.5]nonane-2-carboxylate (IIIa)**

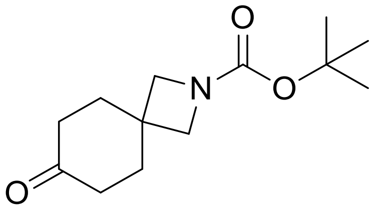

The title compound was synthesized according to GP3 from tert-butyl 7-hydroxy-2-azaspiro[3.5]nonane-2-carboxylate (241 mg, 1 mmol) as a colorless amorphous solid (239 mg, quantitative yield). LC-MS (ESI): m/z = 240.2 $[M+H]^+$

^{1}H NMR (300 MHz, $CDCl_3$) δ 3.75 (s, 4H), 2.35 (t, *J* = 6.6 Hz, 4H), 2.04 (t, *J* = 6.7 Hz, 4H), 1.44 (s, 9H).

^{13}C NMR (75 MHz, $CDCl_3$) δ 209.86, 156.55, 79.76, 58.27, 37.83, 35.65, 34.19, 28.49.

***tert*-Butyl 7-((4,4,5,5-tetramethyl-1,3,2-dioxaborolan-2-yl)methylene)-2-azaspiro[3.5]nonane-2-carboxylate (IVa)**

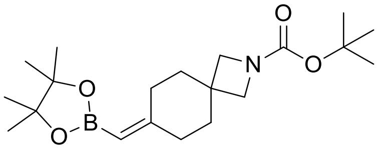

The title compound was synthesized according to GP4 from intermediate **IIIa** (239 mg, 1 mmol) to give the title compound as a colorless viscous oil (360 mg, 99%).

LC-MS (ESI): m/z = 386.2 $[M+Na]^+$

^{1}H NMR (300 MHz, $CDCl_3$) δ 5.07 (s, 1H), 3.62 (s, 4H), 2.55 – 2.45 (m, 2H), 2.21 – 2.13 (m, 2H), 1.80 – 1.70 (m, 4H), 1.44 (s, 9H), 1.25 (s, 12H).

^{13}C NMR (75 MHz, $CDCl_3$) δ 163.79, 156.74, 83.16, 82.86, 79.40, 77.36, 37.47, 37.33, 36.00, 34.95, 29.05, 28.58, 27.05, 24.96, 24.87.

(4a*R*,8a*S*)-6-(7-((4,4,5,5-Tetramethyl-1,3,2-dioxaborolan-2-yl)methylene)-2-azaspiro[3.5]nonane-2-carbonyl)hexahydro-2*H*-pyrido[4,3-b][1,4]oxazin-3(4H)-one (Va)

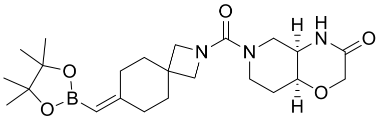

The title compound was synthesized according to GP1 A, from intermediate **IVa** (182 mg, 0.5 mmol) and (4aR,8aS)-3-oxooctahydro-2*H*-pyrido[4,3-b][1,4]oxazin-6-ium 2,3-bis((4-methylbenzoyl)oxy) succinate (372 mg, 0.5 mmol) as a colorless amorphous solid (106 mg, 48%).

LC-MS (ESI): m/z = 446.3 $[M+H]^+$

^{1}H NMR (300 MHz, CD_3CN) δ 6.59 (s, 1H), 5.00 (d, *J* = 1.2 Hz, 1H), 4.19 – 4.00 (m, 2H), 3.94 (d, *J* = 3.1 Hz, 1H), 3.82 – 3.72 (m, 1H), 3.71 – 3.62 (m, 4H), 3.62 – 3.50 (m, 1H), 3.29 (dtd, *J* = 11.6, 4.7, 2.5 Hz, 1H), 3.00 – 2.82 (m, 2H), 2.55 – 2.44 (m, 2H), 2.22 – 2.17 (m, 2H), 1.80 (dd, *J* = 4.8, 3.2 Hz, 1H), 1.77 – 1.67 (m, 5H), 1.22 (s, 12H).

^{13}C NMR (75 MHz, CD_3CN) δ 168.56, 165.27, 163.09, 83.58, 70.31, 68.14, 61.89, 50.18, 46.94, 40.22, 38.40, 38.19, 36.45, 35.66, 30.14, 29.58, 25.11.

((2-((4a*R*,8a*S*)-3-Oxooctahydro-2*H*-pyrido[4,3-b][1,4]oxazine-6-carbonyl)-2-azaspiro[3.5]nonan-7-ylidene)methyl)boronic acid (VIa)

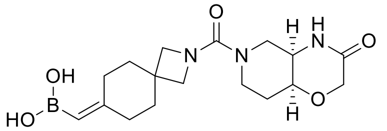

The title compound was synthesized from the boronate **Va** (100 mg, 0.225 mmol) as described in GP5 and obtained as a colorless, hygroscopic solid (58 mg, 71%).

LC-MS (ESI): m/z = 364.2 $[M+H]^+$ HRMS (ESI) m/z $[M+H]^+$ calcd. for $C_{17}H_{26}BN_3O_5$ 362.2002 found 362.2025

^1H NMR (300 MHz, MeOD) δ 5.17 (s, 1H), 4.29 – 4.10 (m, 3H), 4.00 (d, *J* = 3.0 Hz, 2H), 3.87 (dd, *J* = 13.0, 5.1 Hz, 1H), 3.81 – 3.71 (m, 5H), 3.70 – 3.59 (m, 0H), 3.42 – 3.30 (m, 1H), 3.09 – 2.94 (m, 3H), 2.45 – 2.35 (m, 2H), 2.19 (dt, *J* = 22.4, 6.3 Hz, 3H), 1.96 – 1.83 (m, 2H), 1.76 (dt, *J* = 12.7, 6.3 Hz, 4H).

^{13}C NMR (75 MHz, MeOD) δ 171.18, 163.91, 160.23, 70.52, 68.22, 62.54, 50.45, 47.21, 40.62, 38.88, 38.46, 38.42, 36.76, 36.20, 36.14, 30.44, 30.17.

(4a*R*,8a*S*)-6-(7-((5,5-Difluoro-5*H*-4λ⁴,5λ⁴-dipyrrolo[1,2-c:2',1'-f][1,3,2]diazaborinin-10-yl)methylene)-2-azaspiro[3.5]nonane-2-carbonyl)hexahydro-2*H*-pyrido[4,3-b][1,4]oxazin-3(4H)-one (S11 VIIIa)

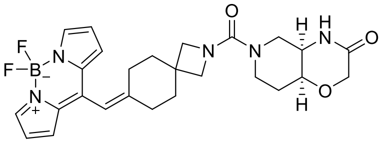

The title compound was synthesized from intermediate **VI** (36.3 mg, 100 µmol) and 8-(methylthio)-4,4,difluoro-4-bora-3a,4a-diaza-s-indacene (23.8 mg, 100 µmol) according to GP6 and obtained as a dark red amorphous solid (20 mg, 39 %).

LC-MS (ESI): m/z = 490.2 $[M-F]^+$

^1H NMR (300 MHz, CD_3CN) δ 7.85 (s, 2H), 7.28 (d, *J* = 4.2 Hz, 2H), 6.65 (s, 1H), 6.61 – 6.53 (m, 2H), 6.47 (s, 1H), 4.20 – 4.01 (m, 2H), 3.95 (d, *J* = 3.1 Hz, 1H), 3.82 – 3.76 (m, 1H), 3.70 (qd, *J* = 8.0, 5.7 Hz, 4H), 3.60 – 3.49 (m, 1H), 3.37 – 3.23 (m, 1H), 3.01 – 2.83 (m, 2H), 2.42 (t, *J* = 6.3 Hz, 2H), 2.26 – 2.15 (m, 2H), 1.88 (d, *J* = 6.2 Hz, 1H), 1.83 – 1.64 (m, 5H).

^{13}C NMR (75 MHz, CD_3CN) δ 168.88, 163.04, 153.61, 144.35, 130.89, 119.16, 115.75, 70.25, 68.08, 61.80, 50.16, 46.89, 40.25, 37.95, 37.68, 35.70, 34.12, 30.10, 28.48.

(4a*R*,8a*S*)-6-(7-((5,5-Difluoro-5*H*-4λ⁴,5λ⁴-dipyrrolo[1,2-c:2',1'-f][1,3,2]diazaborinin-10-yl)methyl)-2-azaspiro[3.5]nonane-2-carbonyl)hexahydro-2*H*-pyrido[4,3-b][1,4]oxazin-3(4H)-one (S12 IXa)

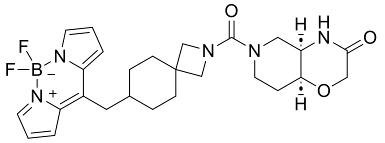

The title compound was synthesized from alkenyl BODIPY **VIIIa** (5.1 mg, 10µmol) according to GP7 and obtained as an amorphous orange solid with green fluorescence in solution (3.2 mg, 63%).

LC-MS (ESI): m/z = 492.2 $[M-F]^+$

HRMS (ESI) m/z $[M+H]^+$ calcd. for $C_{26}H_{32}BF_2N_5O_3$ 510.2603 found 510.2606

^1H NMR (300 MHz, CD_3CN) δ 7.85 (s, 2H), 7.48 (d, *J* = 4.3 Hz, 2H), 6.60 (dd, *J* = 4.0, 1.7 Hz, 2H), 4.18 – 4.02 (m, 2H), 3.98 – 3.88 (m, 1H), 3.82 – 3.63 (m, 3H), 3.60 – 3.48 (m, 4H), 2.98 – 2.84 (m, 3H), 1.88 – 1.74 (m, 4H), 1.69 – 1.57 (m, 2H), 1.46 – 1.09 (m, 6H).

^{13}C NMR (151 MHz, CD_3CN) δ 168.61, 163.12, 151.77, 144.32, 136.70, 130.17, 119.10, 117.31, 108.33, 105.26, 70.36, 68.18, 63.06, 61.59, 50.24, 47.01, 46.98, 42.02, 40.26, 38.29, 36.37, 30.47, 30.15, 29.98.

tert-Butyl 6-((4,4,5,5-tetramethyl-1,3,2-dioxaborolan-2-yl)methylene)-2-azaspiro[3.3]heptane-2-carboxylate (IVb)

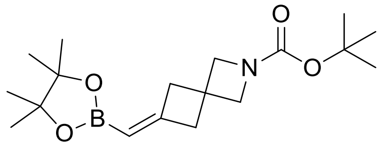

The title compound was synthesized from commercial tert-butyl 6-oxo-2-azaspiro[3.3]heptane-2-carboxylate (105.6 mg, 0.5 mmol) according to GP4 and obtained after flash chromatography as a colorless solid.

LC-MS (ESI): m/z = 358.2 $[M+Na]^+$

^1H NMR (300 MHz, $CDCl_3$) δ 5.18 (p, *J* = 2.3 Hz, 1H), 4.00 – 3.87 (m, 4H), 3.10 – 3.05 (m, 2H), 2.93 (dq, *J* = 3.3, 1.9 Hz, 2H), 1.43 (s, 9H), 1.23 (s, 12H).

^{13}C NMR (75 MHz, $CDCl_3$) δ 161.76, 156.40, 83.15, 82.87, 79.43, 45.50, 45.16, 33.27, 28.54, 24.99, 24.86.

(4a*R*,8a*S*)-6-(6-((4,4,5,5-Tetramethyl-1,3,2-dioxaborolan-2-yl)methylene)-2-azaspiro[3.3]heptane-2-carbonyl)hexahydro-2*H*-pyrido[4,3-b][1,4]oxazin-3(4H)-one (Vb)

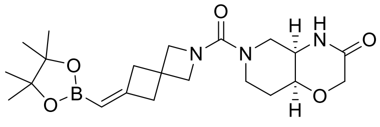

The title compound was synthesized according to GP1 A, from intermediate **IVb** (67 mg, 0.2 mmol) and (4aR,8aS)-3-oxooctahydro-2*H*-pyrido[4,3-b][1,4]oxazin-6-ium 2,3-bis((4-methylbenzoyl)oxy)succinate (149 mg, 0.2 mmol) without column purification. The crude product was subjected to the hydrolytic step without further purification

LC-MS (ESI): m/z = 418.2 $[M+H]^+$

((2-((4a*R*,8a*S*)-3-Oxooctahydro-2*H*-pyrido[4,3-b][1,4]oxazine-6-carbonyl)-2-azaspiro[3.3]heptan-6-ylidene)methyl)boronic acid (VIb)

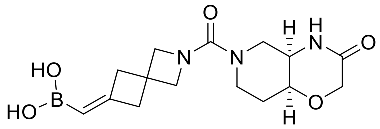

The crude intermediate **Vb** was hydrolyzed according to GP5. The title product was obtained after preparative HPLC as a colorless amorphous solid. LC-MS (ESI): m/z = 336.2 $[M+H]^+$

^1H NMR (300 MHz, MeOD) δ 5.37 – 5.29 (m, 1H), 4.19 (d, *J* = 4.0 Hz, 2H), 4.08 – 3.96 (m, 5H), 3.91 – 3.78 (m, 1H), 3.69 – 3.55 (m, 1H), 3.37 – 3.31 (m, 1H), 3.08 – 2.91 (m, 6H), 1.98 – 1.74 (m, 2H).

^{13}C NMR (75 MHz, MeOD) δ 171.14, 163.67, 160.96, 70.50, 68.21, 64.40, 50.43, 47.15, 46.21, 46.07, 40.57, 34.75, 30.43.

(4a*R*,8a*S*)-6-(6-((5,5-Difluoro-5*H*-4λ⁴,5λ⁴-dipyrrolo[1,2-c:2',1'-f][1,3,2]diazaborinin-10-yl)methylene)-2-azaspiro[3.3]heptane-2-carbonyl)hexahydro-2*H*-pyrido[4,3-b][1,4]oxazin-3(4H)-one (*S*13 XIIIb)

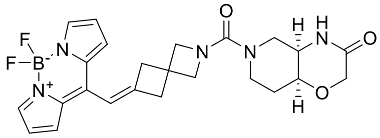

The title compound was synthesized according to GP6 from 8-(methylthio)-4,4,difluoro-4-bora-3a,4a-diaza-s-indacene (21.4 mg, 90 µmol) and boronic acid **VIb** (30.2 mg, 90 µmol) as a dark red amorphous solid (25 mg, 58%).

HRMS (ESI) m/z $[M+H]^+$ calcd. for $C_{24}H_{26}BF_2N_5O_3$ 480.2133 found 480.2112

^{1}H NMR (300 MHz, CD_3CN) δ 7.82 (s, 2H), 7.36 (d, *J* = 4.2 Hz, 2H), 6.70 (p, *J* = 2.2 Hz, 1H), 6.62 (s, 1H), 6.57 (dt, *J* = 3.9, 1.3 Hz, 2H), 4.17 – 4.03 (m, 2H), 4.03 – 3.90 (m, 5H), 3.75 (dd, *J* = 12.9, 4.9 Hz, 1H), 3.51 (ddt, *J* = 13.3, 4.6, 2.3 Hz, 1H), 3.32 – 3.22 (m, 1H), 3.14 (t, *J* = 2.1 Hz, 2H), 3.07 (t, *J* = 2.4 Hz, 2H), 2.98 – 2.84 (m, 2H), 1.86 – 1.76 (m, 2H).

^{13}C NMR (75 MHz, CD_3CN) δ 168.79, 162.77, 155.79, 143.75, 130.52, 116.82, 70.20, 68.06, 63.17, 63.13, 50.12, 46.82, 45.38, 45.32, 40.18, 35.14, 30.07.

(4a*R*,8a*S*)-6-(6-((5,5-Difluoro-5*H*-4λ⁴,5λ⁴-dipyrrolo[1,2-c:2',1'-f][1,3,2]diazaborinin-10-yl)methyl)-2-azaspiro[3.3]heptane-2-carbonyl)hexahydro-2*H*-pyrido[4,3-b][1,4]oxazin-3(4H)-one (4 IXb)

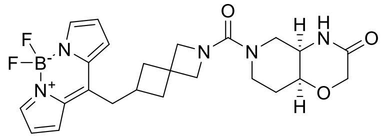

The title compound was obtained from the alkenyl-BODIPY derivate **XIIIb** (24.1 mg, 50 µmol) according to GP7 as an orange amorphous solid with green fluorescence in solution (20 mg, 83%).

HRMS (ESI) m/z $[M+H]^+$ calcd. for $C_{24}H_{28}BF_2N_5O_3$ 482.2290 found 482.2290

^{1}H NMR (600 MHz, CD_3CN) δ 7.85 (s, 2H), 7.49 (d, *J* = 4.3 Hz, 2H), 6.62 – 6.60 (m, 2H), 6.58 (d, *J* = 4.5 Hz, 1H), 4.16 – 4.01 (m, 2H), 3.94 – 3.92 (m, 1H), 3.92 – 3.88 (m, 2H), 3.84 (q, *J* = 8.6 Hz, 2H), 3.73 (ddt, *J* = 13.0, 4.8, 1.3 Hz, 1H), 3.58 (h, *J* = 4.0 Hz, 1H), 3.50 (ddt, *J* = 13.4, 4.6, 2.1 Hz, 1H), 3.26 (dtd, *J* = 11.7, 4.7, 2.4 Hz, 1H), 3.06 (d, *J* = 7.5 Hz, 2H), 2.94 – 2.84 (m, 2H), 2.48 (tt, *J* = 8.8, 7.7 Hz, 1H), 2.24 – 2.18 (m, 2H), 2.01 – 1.96 (m, 2H), 1.73 (dddd, *J* = 14.4, 12.7, 5.1, 3.2 Hz, 1H).

^{13}C NMR (151 MHz, CD_3CN) δ 168.74, 162.87, 144.43, 136.28, 130.13, 119.16, 70.29, 68.12, 64.88, 63.30, 50.20, 46.90, 40.21, 39.88, 37.51, 35.04, 34.56, 30.12.

((2-(6-Oxo-7-oxa-2,5-diazaspiro[3.4]octane-2-carbonyl)-2-azaspiro[3.3]heptan-6-ylidene)methyl)boronic acid (VIc)

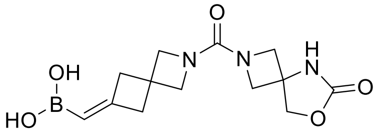

The title compound was synthesized according to GP1 A from intermediate **IVb** (33.5 mg, 100 µmol) and 7-oxa-2,5-diazaspiro[3.4]octan-6-one tosylate (30 mg, 100 µmol), followed by boronate hydrolysis according to GP5. 6.1 mg (20% over 3 steps) of the colorless product was obtained after RP-HPLC purification.

LC-MS (ESI): m/z = 308.1 $[M+H]^+$

^{1}H NMR (300 MHz, MeOD) δ 5.33 (p, *J* = 2.3 Hz, 1H), 4.56 (s, 2H), 4.14 – 4.06 (m, 4H), 3.98 (s, 4H), 3.08 – 3.02 (m, 2H), 2.98 – 2.93 (m, 2H).

^{13}C NMR (75 MHz, MeOD) δ 163.74, 160.75, 160.45, 75.63, 63.82, 63.07, 55.82, 46.12, 45.99, 35.06.

2-(6-((5,5-Difluoro-5*H*-4λ⁴,5λ⁴-dipyrrolo[1,2-c:2',1'-f][1,3,2]diazaborinin-10-yl)methylene)-2-azaspiro[3.3]heptane-2-carbonyl)-7-oxa-2,5-diazaspiro[3.4]octan-6-one (S14 VIIIc)

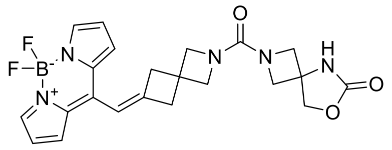

The title compound was synthesized according to GP6 from 8-(methylthio)-4,4,difluoro-4-bora-3a,4a-diaza-s-indacene (4.8 mg, 20 µmol) and boronic acid **VIc** (6.1 mg, 20 µmol) as a dark red amorphous solid (3.5 mg, 39%).

HRMS (ESI) m/z $[M+H]^+$ calcd. for $C_{22}H_{22}BF_2N_5O_3$ 452.1820 found 452.1734

^{1}H NMR (300 MHz, CD_3CN) δ 7.81 (s, 2H), 7.36 (d, *J* = 4.2 Hz, 2H), 6.70 (t, *J* = 2.2 Hz, 1H), 6.61 – 6.53 (m, 2H), 6.48 (s, 1H), 4.45 (s, 2H), 4.05 – 3.84 (m, 8H), 3.14 (d, *J* = 2.0 Hz, 2H), 3.07 (t, *J* = 2.4 Hz, 2H).

^{13}C NMR (75 MHz, CD_3CN) δ 158.42, 155.62, 144.46, 143.75, 134.48, 130.56, 121.25, 116.84, 75.10, 63.47, 62.01, 55.18, 45.30, 45.28, 35.45.

2-(6-((5,5-Difluoro-5*H*-4λ⁴,5λ⁴-dipyrrolo[1,2-c:2',1'-f][1,3,2]diazaborinin-10-yl)methyl)-2-azaspiro[3.3]heptane-2-carbonyl)-7-oxa-2,5-diazaspiro[3.4]octan-6-one (S15 IXc)

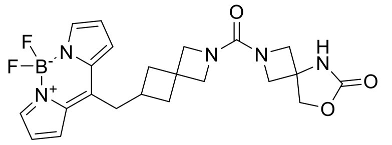

The title compound was obtained from the alkenyl-BODIPY derivate **XIIIc** (3.5 mg, 6.6 µmol) according to GP7 as an orange amorphous solid with green fluorescence in solution (2.7 mg, ca. 77%).

HRMS (ESI) m/z $[M+H]^+$ calcd. for $C_{22}H_{24}BF_2N_5O_3$ 454.1977 found 454.1974

^{1}H NMR (600 MHz, CD_3CN) δ 7.85 (s, 2H), 7.48 (d, *J* = 4.2 Hz, 2H), 6.62 – 6.59 (m, 2H), 6.41 (s, 1H), 4.45 (s, 2H), 4.00 – 3.93 (m, 4H), 3.84 (s, 2H), 3.77 (s, 2H), 3.05 (d, *J* = 7.5 Hz, 2H), 2.47 (hept, *J* = 8.0 Hz, 1H), 1.98 (td, *J* = 9.2, 3.2 Hz, 4H).

^{13}C NMR (151 MHz, CD_3CN) δ 158.43, 150.85, 144.43, 136.27, 130.12, 119.16, 75.16, 63.71, 63.45, 62.10, 55.21, 39.80, 37.49, 35.38, 34.51.

2-(7-((4,4,5,5-Tetramethyl-1,3,2-dioxaborolan-2-yl)methylene)-2-azaspiro[3.5]nonane-2-carbonyl)-7-oxa-2,5-diazaspiro[3.4]octan-6-one (Vd)

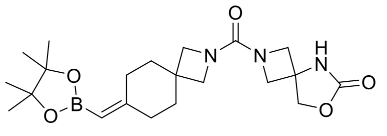

The title compound was obtained from intermediate **IVa** (181.7 mg, 500 µmol) and 7-oxa-2,5-diazaspiro[3.4]octan-6-one tosylate (150.2 mg, 500 µmol) according to GP1 A as a colorless amorphous solid (74 mg, 35%).

m/z = 418.2 $[M+H]^+$ HRMS (ESI) m/z $[M+H]^+$ calcd. for $C_{21}H_{32}BN_3O_5$ 418.2540 found 418.2513

^{1}H NMR (300 MHz, $CDCl_3$) δ 7.15 (s, 1H), 5.07 (s, 1H), 4.51 (s, 2H), 4.16 (d, *J* = 9.3 Hz, 2H), 3.99 (d, *J* = 9.2 Hz, 2H), 3.64 (s, 4H), 2.48 (t, *J* = 6.3 Hz, 2H), 2.16 (t, *J* = 6.2 Hz, 2H), 1.74 (dt, *J* = 9.1, 4.7 Hz, 4H), 1.24 (s, 12H).

^{13}C NMR (75 MHz, $CDCl_3$) δ 163.02, 162.73, 158.07, 82.89, 74.84, 62.89, 60.25, 54.53, 37.49, 37.36, 35.80, 35.76, 28.88, 24.93.

((2-(6-Oxo-7-oxa-2,5-diazaspiro[3.4]octane-2-carbonyl)-2-azaspiro[3.5]nonan-7-ylidene)-methyl)boronic acid (VId)

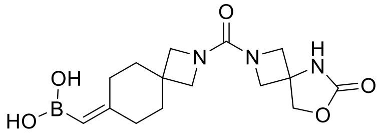

The title compound was obtained from the boronate **Vd** (73 mg, 175 mmol) according to GP5 and RP-HPLC purification as a colorless amorphous solid (42.5 mg, 72%).

HRMS (ESI) m/z $[M+H]^+$ calcd. for $C_{15}H_{22}BN_3O_5$ 334.1689 found 334.1711

^{1}H NMR (300 MHz, MeOD) δ 5.17 (s, 1H), 4.57 (s, 2H), 4.12 (d, *J* = 1.4 Hz, 4H), 3.70 (s, 4H), 2.39 (t, *J* = 6.4 Hz, 2H), 2.22 (t, *J* = 6.4 Hz, 2H), 1.76 (dt, *J* = 12.7, 6.5 Hz, 4H).

^{13}C NMR (75 MHz, MeOD) δ 164.00, 160.54, 160.07, 75.70, 69.41, 63.86, 61.19, 55.87, 38.78, 38.36, 36.68, 30.09.

2-(7-((5,5-Difluoro-5*H*-4λ⁴,5λ⁴-dipyrrolo[1,2-c:2',1'-f][1,3,2]diazaborinin-10-yl)methylene)-2-azaspiro[3.5]nonane-2-carbonyl)-7-oxa-2,5-diazaspiro[3.4]octan-6-one (*S*16 VIIId)

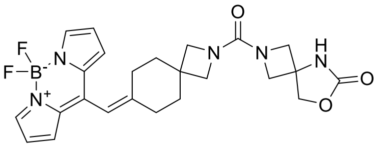

The title compound was synthesized according to GP6 from intermediate **VId** (33.5 mg, 0.1 mmol). It was isolated as an intensely red-colored amorphous solid (14.4 mg, 29%).

HRMS (ESI) m/z $[M+H]^+$ calcd. for $C_{24}H_{26}BF_2N_5O_3$ 480.2133 found 480.2172

^{1}H NMR (600 MHz, CD_3CN) δ 7.87 (s, 2H), 7.29 (d, *J* = 4.2 Hz, 2H), 6.60 – 6.56 (m, 2H), 6.53 (s, 1H), 6.48 (s, 1H), 4.49 (s, 2H), 4.05 (d, *J* = 9.3 Hz, 2H), 4.00 (d, *J* = 9.3 Hz, 2H), 3.66 (d, *J* = 8.0 Hz, 2H), 3.62 (d, *J* = 7.9 Hz, 2H), 2.42 (t, *J* = 6.3 Hz, 2H), 2.21 (t, *J* = 6.5 Hz, 2H), 1.91 (t, *J* = 6.2 Hz, 2H), 1.69 (t, *J* = 6.3 Hz, 2H).

^{13}C NMR (151 MHz, CD_3CN) δ 163.40, 158.45, 153.51, 145.70, 144.39, 135.76, 130.91, 119.16, 115.79, 75.21, 63.50, 60.59, 55.23, 37.91, 37.64, 36.26, 34.09, 28.47, 25.15.

2-(7-((5,5-Difluoro-5*H*-4λ4,5λ4-dipyrrolo[1,2-c:2',1'-f][1,3,2]diazaborinin-10-yl)methyl)-2-azaspiro[3.5]nonane-2-carbonyl)-7-oxa-2,5-diazaspiro[3.4]octan-6-one (3 IXd)

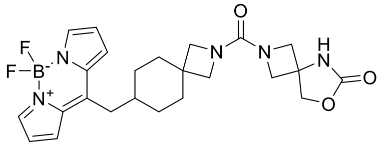

The title compound was obtained from alkenyl BODIPY compound **VIIId** (4.8 mg, 10 µmol) according to GP7 as an orange amorphous solid with green fluorescence in solution (1.34 mg, 28%).

HRMS (ESI) m/z $[M+H]^+$ calcd. for $C_{24}H_{28}BF_2N_5O_3$ 482.2290 found 482.2292

^{1}H NMR (600 MHz, CD_3CN) δ 7.85 (s, 2H), 7.47 (d, *J* = 4.3 Hz, 2H), 6.60 (dd, *J* = 4.6, 1.9 Hz, 2H), 6.52 (s, 1H), 4.46 (s, 2H), 4.02 (d, *J* = 9.1 Hz, 2H), 3.97 (d, *J* = 9.3 Hz, 2H), 3.63 – 3.55 (m, 4H), 3.47 (s, 2H), 2.88 (d, *J* = 7.2 Hz, 2H), 1.76 – 1.65 (m, 1H), 1.62 (dd, *J* = 13.2, 3.9 Hz, 2H), 1.38 (td, *J* = 13.3, 3.6 Hz, 2H), 1.12 (qd, *J* = 13.6, 3.3 Hz, 2H).

^{13}C NMR (151 MHz, CD_3CN) δ 158.55, 151.74, 144.33, 136.70, 130.18, 119.11, 75.22, 63.51, 61.77, 60.46, 55.24, 41.95, 38.24, 36.24, 36.15, 30.41.

(1-((4a*R*,8a*S*)-3-Oxooctahydro-2*H*-pyrido[4,3-b][1,4]oxazine-6-carbonyl)-1,2,3,6-tetrahydropyridin-4-yl)boronic acid (VIe)

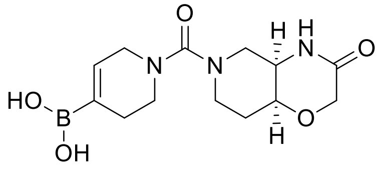

The corresponding boronate was synthesized according to GP1 B from tert-butyl 4-(4,4,5,5-tetramethyl-1,3,2-dioxaborolan-2-yl)-3,6-dihydropyridine-1(2H)-carboxylate (61.8 mg, 200 µmol) and (4aR,8aS)-3-oxooctahydro-2*H*-pyrido[4,3-b][1,4]oxazin-6-ium 2,3-bis((4-methylbenzoyl)oxy)succinate (148.8 mg, 200µmol). The obtained crude (49 mg) was subjected to hydrolytic conditions according to GP5 without further purification. The title compound was obtained as a colorless solid (10 mg, 16% over 3 steps).

LC-MS (ESI) m/z = 310.1 $[M+H]^+$

^{1}H NMR (300 MHz, MeOD) δ 6.35 – 6.10 (m, 1H), 4.29 – 4.10 (m, 2H), 4.01 (d, *J* = 3.0 Hz, 1H), 3.85 (s, 2H), 3.64 (dd, *J* = 12.8, 4.9 Hz, 1H), 3.50 – 3.34 (m, 2H), 3.14 – 2.96 (m, 2H), 2.26 (s, 2H), 1.99 – 1.86 (m, 2H).

^{13}C NMR (75 MHz, MeOD) δ 171.22, 165.23, 70.61, 68.20, 50.41, 44.82, 42.65, 30.32.

5,5-Difluoro-10-(1-((4a*R*,8a*S*)-3-oxooctahydro-2*H*-pyrido[4,3-b][1,4]oxazine-6-carbonyl)-1,2,3,6-tetrahydropyridin-4-yl)-5*H*-4l4-dipyrrolo[1,2-c:2',1'-f][1,3,2]diazaborinin-5-uide (S9 VIIIe)

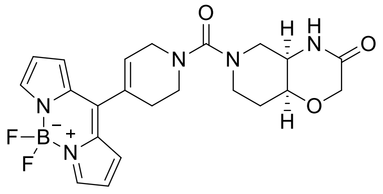

The title compound was synthesized from boronic acid intermediate **VIe** (8.3 mg, 27 µmol) according to GP6 as a deep red amorphous solid (3.1 mg, 25%).

LC-MS (ESI): m/z = 436.2 $[M-F]^+$

^{1}H NMR (600 MHz, CD3CN) δ 7.89 (s, 2H), 7.30 (d, *J* = 4.2 Hz, 2H), 6.64 (d, *J* = 4.4 Hz, 1H), 6.60 (d, *J* = 3.0 Hz, 2H), 6.09 (tt, *J* = 3.3, 1.6 Hz, 1H), 4.14 (q, 2H), 3.99 (p, *J* = 2.9 Hz, 3H), 3.67 – 3.62 (m, 1H), 3.51 (ddd, *J* = 13.2, 6.1, 4.6 Hz, 1H), 3.44 (ddd, *J* = 12.9, 6.8, 4.4 Hz, 3H), 3.02 (ddd, *J* = 13.3, 9.4, 6.6 Hz, 2H), 2.68 – 2.57 (m, 2H), 1.88 (dq, *J* = 6.6, 3.4 Hz, 2H).

^{13}C NMR (151 MHz, CD_3CN) δ 168.83, 164.15, 150.16, 145.16, 135.11, 131.99, 131.60, 119.47, 70.38, 68.13, 50.19, 48.81, 47.13, 44.53, 42.36, 32.07, 30.06.

***tert*-Butyl 4-((4,4,5,5-tetramethyl-1,3,2-dioxaborolan-2-yl)methylene)piperidine-1-carboxylate (IVf)**

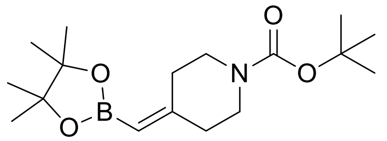

The title compound was synthesized from tert-butyl 4-oxopiperidine-1-carboxylate (99.6 mg, 0.5 mmol) according to GP4 as a colorless amorphous solid (115 mg, 71%).

LC-MS (ESI): m/z = 324.2 $[M+H]^+$

^{1}H NMR (300 MHz, $CDCl_3$) δ 5.15 (t, *J* = 1.0 Hz, 1H), 3.50 – 3.39 (m, 4H), 2.65 – 2.54 (m, 2H), 2.25 (t, *J* = 5.9 Hz, 2H), 1.46 (s, 9H), 1.26 (s, 12H).

^{13}C NMR (75 MHz, $CDCl_3$) δ 161.55, 154.89, 82.98, 79.63, 77.37, 38.86, 32.75, 28.60, 24.98.

((1-((4a*R*,8a*S*)-3-Oxooctahydro-2*H*-pyrido[4,3-b][1,4]oxazine-6-carbonyl)piperidin-4-ylidene)methyl)boronic acid (VIf)

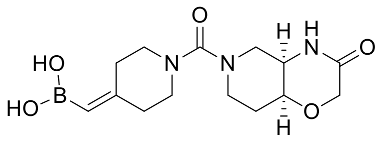

The intermediate Vf was synthesized from intermediate **IVf** (48.5 mg, 150μmol) and (4aR,8aS)-3-oxooctahydro-2*H*-pyrido[4,3-b][1,4]oxazin-6-ium 2,3-bis((4-methylbenzoyl)oxy)succinate (111.6 mg, 150μmol) according to GP1 B. The crude colorless amorphous solid (26 mg) was subjected to hydrolytic conditions according to GP5 to obtain the title compound after HPLC purification (10mg, 21% over 3 steps).

LC-MS (ESI): m/z = 324.2 $[M+H]^+$

^{1}H NMR (300 MHz, MeOD) δ 5.28 (s, 1H), 4.29 – 4.10 (m, 2H), 4.01 (d, *J* = 3.1 Hz, 1H), 3.73 – 3.61 (m, 1H), 3.44 (ddd, *J* = 10.9, 5.0, 2.5 Hz, 2H), 3.34 (d, *J* = 5.0 Hz, 1H), 3.29 – 3.25 (m, 2H), 3.16 – 2.97 (m, 2H), 2.59 – 2.49 (m, 2H), 2.38 – 2.20 (m, 2H), 1.91 (dq, *J* = 6.2, 3.4 Hz, 2H).

(4a*R*,8a*S*)-6-(4-((5,5-Difluoro-5*H*-4λ⁴,5λ⁴-dipyrrolo[1,2-c:2',1'-f][1,3,2]diazaborinin-10-yl)methylene)piperidine-1-carbonyl)hexahydro-2*H*-pyrido[4,3-b][1,4]oxazin-3(4H)-one (VIIIf)

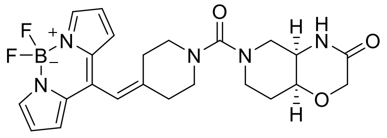

The title compound was synthesized from the boronic acid intermediate **VIf** (9.7 mg, 30 μmol) and 8-(methylthio)-4,4,difluoro-4-bora-3a,4a-diaza-s-indacene (7.1 mg, 30 μmol) according to GP6 and obtained after HPLC purification as a dark red amorphous solid (6.17 mg, 44%).

HRMS (ESI) m/z $[M+H]^+$ calcd. for $C_{23}H_{26}BF_2N_5O_3$ 468.2133 found 468.2179

^{1}H NMR (300 MHz, CD_3CN) δ 7.86 (s, 2H), 7.31 (d, *J* = 4.2 Hz, 2H), 6.69 (s, 1H), 6.60 – 6.56 (m, 3H), 4.19 – 4.03 (m, 2H), 3.95 (q, *J* = 3.0 Hz, 1H), 3.65 – 3.53 (m, 1H), 3.46 – 3.33 (m, 4H), 3.15 (td, *J* = 5.4, 2.2 Hz, 2H), 3.03 – 2.90 (m, 2H), 2.51 (t, *J* = 5.7 Hz, 2H), 2.35 – 2.28 (m, 2H), 1.83 (dt, *J* = 5.9, 3.4 Hz, 2H).

^{13}C NMR (75 MHz, CD_3CN) δ 169.01, 164.06, 151.61, 145.04, 144.48, 135.60, 131.00, 119.24, 116.76, 70.27, 68.01, 50.05, 48.96, 48.85, 48.76, 42.54, 37.06, 32.22, 29.95.

(6-(3-Cyclopropyl-1*H*-1,2,4-triazol-1-yl)-2-azaspiro[3.3]heptan-2-yl)(6-((4,4,5,5-tetramethyl-1,3,2-dioxaborolan-2-yl)methylene)-2-azaspiro[3.3]heptan-2-yl)methanone (VIg)

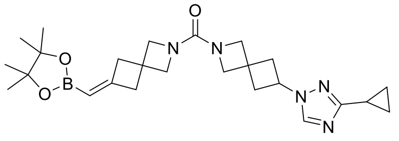

The intermediate **VIg** was synthesized according to GP1 A from **IVb** (67.1 mg, 0.2 mmol) and 6-(3-cyclopropyl-1*H*-1,2,4-triazol-1-yl)-2-azaspiro[3.3]heptan-2-ium triflate (63.7 mg, 0.2 mmol). The title compound was obtained after RP-HPLC purification as a colorless amorphous solid (59 mg, 63 %).

LC-MS (ESI): m/z = 488.2 $[M+Na]^+$

^{1}H NMR (300 MHz, CD_3CN) δ 8.70 (s, 1H), 5.15 – 5.04 (m, 1H), 4.81 (p, *J* = 7.8 Hz, 1H), 4.01 (s, 2H), 3.98 – 3.91 (m, 6H), 3.03 (p, *J* = 2.4 Hz, 2H), 2.96 – 2.89 (m, 2H), 2.79 – 2.60 (m, 4H), 2.12 (tt, *J* = 8.3, 5.0 Hz, 1H), 1.20 (s, 12H), 1.14 – 1.04 (m, 2H), 1.03 – 0.96 (m, 2H).

^{13}C NMR (75 MHz, CD_3CN) δ 163.15, 162.96, 162.16, 141.69, 83.61, 62.76, 62.68, 62.10, 51.09, 45.89, 45.49, 40.29, 34.54, 33.42, 25.15, 8.99, 8.07.

((2-(6-(3-Cyclopropyl-1*H*-1,2,4-triazol-1-yl)-2-azaspiro[3.3]heptane-2-carbonyl)-2-azaspiro[3.3]heptan-6-ylidene)methyl)boronic acid (VIIg)

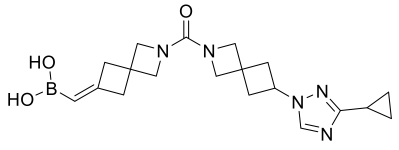

The title compound was obtained from intermediate **VIg** (58.2 mg, 125 µmol) according to GP5 and purified via RP-HPLC to give a colorless amorphous solid (39 mg, 81%).

LC-MS (ESI): m/z = 384.2 $[M+H]^+$

^{1}H NMR (300 MHz, MeOD) δ 8.68 (s, 1H), 5.37 – 5.29 (m, 1H), 4.84 (q, *J* = 7.9 Hz, 1H), 4.07 (s, 2H), 4.01 (s, 2H), 3.98 (s, 4H), 3.05 (d, *J* = 2.7 Hz, 2H), 2.95 (d, *J* = 2.1 Hz, 2H), 2.74 (dt, *J* = 7.9, 2.3 Hz, 4H), 2.06 (tt, *J* = 8.4, 5.0 Hz, 1H), 1.10 – 1.00 (m, 2H), 0.95 (ddd, *J* = 7.7, 5.5, 2.9 Hz, 2H).

^{13}C NMR (75 MHz, MeOD) δ 165.12, 164.12, 160.86, 143.25, 63.26, 63.14, 62.42, 50.51, 46.17, 46.04, 40.90, 35.13, 33.73, 8.94, 8.58.

(6-(3-Cyclopropyl-1*H*-1,2,4-triazol-1-yl)-2-azaspiro[3.3]heptan-2-yl)(6-((5,5-difluoro-5*H*-4λ⁴,5λ⁴-dipyrrolo[1,2-c:2',1'-f][1,3,2]diazaborinin-10-yl)methylene)-2-azaspiro[3.3]heptan-2-yl)methanone (VIIIg)

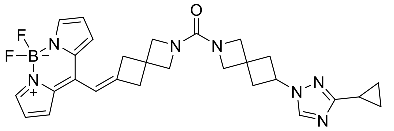

The title compound was synthesized from intermediate **VIIg** (38.3 mg, 100 µmol) according to GP6 and purified via RP-HPLC to give a dark red-colored amorphous compound (17 mg, 32%).

HRMS (ESI) m/z $[M+H]^+$ calcd. for $C_{28}H_{30}BF_2N_7O$ 528.2609 found 528.2613

^{1}H NMR (300 MHz, CD_3CN) δ 8.54 (s, 1H), 7.82 (s, 2H), 7.36 (d, *J* = 4.2 Hz, 2H), 6.70 (q, *J* = 2.2 Hz, 1H), 6.57 (dd, *J* = 4.5, 1.9 Hz, 2H), 4.77 (p, *J* = 7.8 Hz, 1H), 3.95 (d, *J* = 7.7 Hz, 4H), 3.90 (t, *J* = 4.3 Hz, 4H), 3.16 – 3.12 (m, 2H), 3.09 – 3.05 (m, 2H), 2.77 – 2.56 (m, 4H), 2.09 (tt, *J* = 8.3, 5.0 Hz, 1H), 1.10 – 1.00 (m, 2H), 0.99 – 0.92 (m, 2H).

^{13}C NMR (75 MHz, CD_3CN) δ 163.31, 162.97, 155.69, 144.47, 143.77, 142.03, 134.49, 130.53, 116.86, 62.71, 62.05, 61.99, 50.74, 45.33, 45.28, 40.42, 35.49, 33.37, 8.78, 8.34.

(6-(3-Cyclopropyl-1*H*-1,2,4-triazol-1-yl)-2-azaspiro[3.3]heptan-2-yl)(6-((5,5-difluoro-5*H*-4λ⁴,5λ⁴-dipyrrolo[1,2-c:2',1'-f][1,3,2]diazaborinin-10-yl)methyl)-2-azaspiro[3.3]heptan-2-yl)methanone (5 IXg)

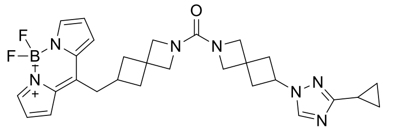

The title compound was synthesized from intermediate **VIIIg** (7.04 mg, 13.3 µmol) according to GP7 (30 equiv. triethylsilane used) and obtained after RP-HPLC as an amorphous orange compound with green fluorescence in solution (5.14 mg, 73%).

HRMS (ESI) m/z $[M+H]^+$ calcd. for $C_{28}H_{32}BF_2N_7O$ 530.2766 found 530.2775

^{1}H NMR (300 MHz, CD_3CN) δ 8.53 (d, *J* = 1.2 Hz, 1H), 7.85 (s, 2H), 7.50 – 7.47 (m, 2H), 6.61 (dd, *J* = 3.0, 1.3 Hz, 1H), 4.77 (tt, *J* = 8.6, 7.3 Hz, 1H), 3.96 – 3.79 (m, 8H), 3.05 (dd, *J* = 7.6, 1.2 Hz, 2H), 2.78 – 2.58

(m, 4H), 2.55 – 2.41 (m, 1H), 2.27 – 2.16 (m, 2H), 2.14 – 2.05 (m, 2H), 2.04 (s, 2H), 1.12 – 1.01 (m, 2H), 1.00 – 0.92 (m, 2H).

^{13}C NMR (75 MHz, CD_3CN) δ 150.84, 144.42, 142.05, 136.23, 130.11, 119.14, 63.73, 62.72, 62.06, 62.00, 50.74, 40.43, 39.75, 37.44, 35.36, 34.50, 33.34, 8.77, 8.34.

(*R*)-4-(3-Oxo-3-(6-((4,4,5,5-tetramethyl-1,3,2-dioxaborolan-2-yl)methylene)-2-azaspiro[3.3]heptan-2-yl)propyl)oxazolidin-2-one (VIh)

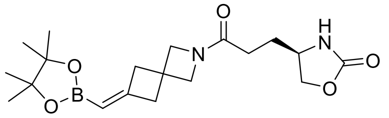

Intermediate **IVb** (33.5 mg, 100 µmol) was boc deprotected with TFA/DCM (1:4) as described before. (*R*)-3-(2-oxooxazolidin-4-yl)propanoic acid (31.8 mg, 200 µmol) and HATU (76 mg, 200 µmol) were dissolved in 1 mL anhydrous DCM and 69 µL TEA (500 µmol) was added. After stirring for 45 min at rt, the deprotected amine was added in 1 mL anhydrous DCM solution. The mixture was concentrated after 2 h, and the crude product was purified via RP-HPLC chromatography. The title compound was obtained as a colorless amorphous solid (30 mg, 80%).

LC-MS (ESI): m/z = 377.2 $[M+H]^+$

^{1}H NMR (300 MHz, $CDCl_3$) δ 8.32 (s, 1H), 6.82 (s, 1H), 5.21 (p, *J* = 2.3 Hz, 1H), 4.56 – 4.45 (m, 1H), 4.17 (s, 2H), 4.09 – 3.90 (m, 4H), 3.12 (d, *J* = 2.7 Hz, 2H), 2.97 (d, *J* = 2.2 Hz, 2H), 2.20 (t, *J* = 7.1 Hz, 2H), 1.96 (dtd, *J* = 14.6, 7.3, 4.3 Hz, 1H), 1.80 (dq, *J* = 13.8, 6.9 Hz, 1H), 1.23 (s, 12H).

^{13}C NMR (75 MHz, $CDCl_3$) δ 172.16, 160.54, 160.19, 82.98, 70.30, 52.14, 45.31, 45.07, 33.14, 29.92, 26.77, 24.94.

(*R*)-((2-(3-(2-Oxooxazolidin-4-yl)propanoyl)-2-azaspiro[3.3]heptan-6-ylidene)methyl)boronic acid (VIIh)

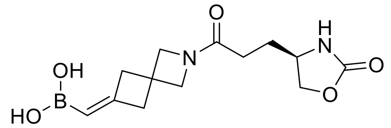

The title compound was synthesized according to GP5 from intermediate **VIh** (30 mg, 80 µmol) and obtained after RP-HPLC on PolarTec column as a colorless, hygroscopic solid (11 mg, 47%).

LC-MS (ESI): m/z = 295.1 $[M+H]^+$

^{1}H NMR (300 MHz, MeOD) δ 5.39 – 5.31 (m, 1H), 4.49 (t, *J* = 8.6 Hz, 1H), 4.22 (s, 2H), 4.07 (dd, *J* = 8.7, 5.9 Hz, 1H), 3.98 (s, 2H), 3.97 – 3.85 (m, 1H), 3.13 – 2.95 (m, 3H), 2.91 (t, *J* = 2.4 Hz, 1H), 2.31 – 2.09 (m, 2H), 1.94 – 1.69 (m, 2H).

^{13}C NMR (75 MHz, MeOD) δ 174.12, 162.17, 160.54, 143.80, 107.58, 71.25, 62.99, 60.66, 53.33, 46.01, 45.89, 43.50, 34.29, 31.01, 27.53.

(*R*)-4-(3-(6-((5,5-Difluoro-5*H*-4λ^4,5λ^4-dipyrrolo[1,2-c:2',1'-f][1,3,2]diazaborinin-10-yl)methylene)-2-azaspiro[3.3]heptan-2-yl)-3-oxopropyl)oxazolidin-2-one (VIIIh)

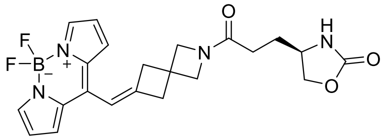

The title compound was synthesized from intermediate **VIIh** (10.6 mg, 36 µmol) according to GP6 and obtained after RP-HPLC purification as an intensely red-colored amorphous solid (9.7 mg, 61%).

LC-MS (ESI): m/z = 421.1 $[M-F]^+$

^{1}H NMR (300 MHz, CD_3CN) δ 7.82 (s, 2H), 7.36 (d, *J* = 4.2 Hz, 2H), 6.71 (p, *J* = 2.1 Hz, 1H), 6.61 – 6.53 (m, 2H), 6.00 (s, 1H), 4.39 (t, *J* = 8.5 Hz, 1H), 4.16 (d, *J* = 5.6 Hz, 2H), 3.95 (dd, *J* = 8.6, 6.0 Hz, 3H), 3.82 (dq, *J* = 8.2, 6.1 Hz, 1H), 3.17 (d, *J* = 2.0 Hz, 2H), 3.10 (t, *J* = 2.4 Hz, 2H), 2.09 (t, *J* = 7.9 Hz, 2H), 1.85 – 1.61 (m, 2H).

^{13}C NMR (75 MHz, CD_3CN) δ 173.11, 160.10, 155.35, 144.42, 143.80, 134.50, 130.55, 118.85, 116.97, 70.59, 62.00, 59.75, 52.72, 45.20, 45.11, 34.68, 30.53, 27.58.

(*R*)-4-(3-(6-((5,5-Difluoro-5*H*-4λ⁴,5λ⁴-dipyrrolo[1,2-c:2',1'-f][1,3,2]diazaborinin-10-yl)methyl)-2-azaspiro[3.3]heptan-2-yl)-3-oxopropyl)oxazolidin-2-one (S17 IXh)

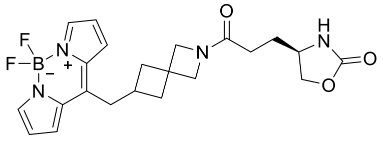

The title compound was synthesized from intermediate **VIIIh** (9.7 mg, 22 μmol) according to GP7. After RP-HPLC purification, it was obtained as an amorphous orange solid with green fluorescence in solution (9.1 mg, 93%).

HRMS (ESI) m/z [M+H]⁺ calcd. for $C_{22}H_{25}BF_2N_4O_3$ 441.2024 found 441.2043

^{1}H NMR (300 MHz, CD_3CN) δ 7.85 (tt, *J* = 1.6, 0.8 Hz, 2H), 7.49 (dd, *J* = 3.6, 1.1 Hz, 2H), 6.64 – 6.59 (m, 2H), 6.08 – 5.82 (m, 1H), 4.39 (t, *J* = 8.4 Hz, 1H), 4.05 (dtd, *J* = 22.4, 1.4, 0.7 Hz, 2H), 3.98 – 3.91 (m, 1H), 3.89 – 3.77 (m, 3H), 3.06 (d, *J* = 7.5 Hz, 2H), 2.61 – 2.41 (m, 1H), 2.28 – 2.19 (m, 2H), 2.11 – 1.98 (m, 4H), 1.79 – 1.66 (m, 2H).

^{13}C NMR (75 MHz, CD_3CN) δ 173.03, 160.01, 150.77, 144.44, 136.24, 130.11, 119.19, 70.56, 59.68, 52.72, 39.56, 37.42, 34.41, 30.56, 27.54.

(*S*)-((2-(3-Carbamoylpyrrolidine-1-carbonyl)-2-azaspiro[3.3]heptan-6-ylidene)methyl)boronic acid (VIIi)

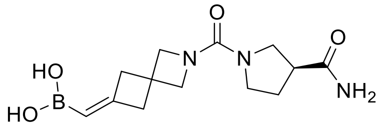

(*S*)-1-(6-((4,4,5,5-tetramethyl-1,3,2-dioxaborolan-2-yl)methylene)-2-azaspiro[3.3]heptane-2-carbonyl)pyrrolidine-3-carboxamide was synthesized according to GP1 B from intermediate **IVb** (67 mg, 200 μmol) and (S)-pyrrolidine-3-carboxamide hydrochloride (60.2 mg, 400 μmol). A crude product was obtained as a colorless amorphous solid (~ 57 mg, ~ 76 %) after liquid extraction and subjected to hydrolytic conditions according to GP5 to give the title compound as a colorless amorphous solid (15 mg, 26% over 3 steps).

LC-MS (ESI): m/z = 294.1 [M+H]⁺

^{1}H NMR (300 MHz, MeOD) δ 5.36 – 5.28 (m, 1H), 4.02 (q, *J* = 8.4 Hz, 4H), 3.63 – 3.31 (m, 4H), 3.05 (d, *J* = 2.6 Hz, 2H), 3.03 – 2.97 (m, 1H), 2.95 (d, *J* = 2.1 Hz, 2H), 2.23 – 1.95 (m, 2H).

(*S*)-1-(6-((5,5-Difluoro-5*H*-4λ⁴,5λ⁴-dipyrrolo[1,2-c:2',1'-f][1,3,2]diazaborinin-10-yl)methylene)-2-azaspiro[3.3]heptane-2-carbonyl)pyrrolidine-3-carboxamide (VIIIi)

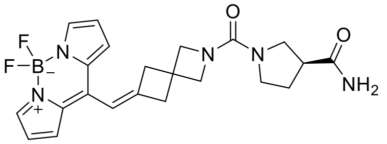

The title compound was synthesized according to GP6 from the intermediate **VIIi** (14.7 mg, 50 μmol). It was obtained as a dark red amorphous solid (5.72 mg, 26%).

LC-MS (ESI): m/z = 440.2 [M+H]⁺

^{1}H NMR (300 MHz, CD_3CN) δ 7.81 (s, 2H), 7.36 (d, *J* = 4.3 Hz, 2H), 6.70 (p, *J* = 2.2 Hz, 1H), 6.61 – 6.52 (m, 2H), 6.27 (s, 1H), 5.77 (s, 1H), 4.06 – 3.92 (m, 4H), 3.53 – 3.39 (m, 2H), 3.37 (dd, *J* = 9.0, 4.6 Hz, 2H), 3.27 (dt, *J* = 10.1, 7.6 Hz, 1H), 3.14 (d, *J* = 2.0 Hz, 2H), 3.06 (d, *J* = 2.5 Hz, 2H), 2.99 – 2.82 (m, 1H), 2.11 – 1.99 (m, 1H).

^{13}C NMR (75 MHz, CD_3CN) δ 175.69, 155.85, 144.51, 143.74, 134.49, 130.54, 118.80, 116.82, 62.60, 50.09, 47.22, 45.28, 45.27, 44.08, 35.00, 29.96.

(*S*)-1-(6-((5,5-Difluoro-5*H*-4λ^4,5λ^4-dipyrrolo[1,2-c:2',1'-f][1,3,2]diazaborinin-10-yl)methyl)-2-azaspiro[3.3]heptane-2-carbonyl)pyrrolidine-3-carboxamide (IXi)

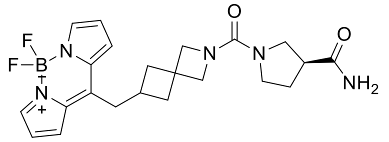

The title compound was synthesized according to GP7 from intermediate **VIIIi** (5.71 mg, 13 µmol). It was obtained as an amorphous orange solid (4 mg, 70%) with green fluorescence in solution.

HRMS (ESI) m/z [M+H]$^+$ calcd. for $C_{22}H_{26}BF_2N_5O_2$ 440.2184 found 440.2202

^{1}H NMR (300 MHz, CD_3CN) δ 7.85 (s, 2H), 7.49 (d, *J* = 4.3 Hz, 2H), 6.61 (dd, *J* = 4.7, 1.9 Hz, 2H), 6.23 (s, 1H), 5.73 (s, 1H), 3.95 – 3.88 (m, 2H), 3.87 – 3.80 (m, 2H), 3.45 (dd, *J* = 10.3, 7.7 Hz, 1H), 3.35 (dd, *J* = 10.1, 6.7 Hz, 2H), 3.25 (dt, *J* = 10.1, 7.6 Hz, 2H), 3.05 (d, *J* = 7.5 Hz, 2H), 2.89 (p, *J* = 7.6 Hz, 1H), 2.48 (hept, *J* = 7.9 Hz, 1H), 2.25 – 2.14 (m, 2H), 1.98 (s, 3H).

^{13}C NMR (75 MHz, CD_3CN) δ 175.61, 150.91, 144.39, 136.23, 130.12, 119.15, 64.25, 62.69, 50.09, 47.20, 44.11, 39.78, 37.52, 34.84, 34.54, 29.96.

6-(6-((4,4,5,5-Tetramethyl-1,3,2-dioxaborolan-2-yl)methylene)-2-azaspiro[3.3]heptane-2-carbonyl)-2*H*-benzo[b][1,4]oxazin-3(4*H*)-one (VIg)

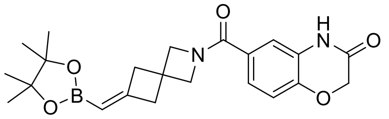

The title compound was synthesized in the same fashion as **VIh** from 3-oxo-3,4-dihydro-2*H*-benzo[b][1,4]oxazine-6-carboxylic acid (29 mg, 150 µmol) and **IVb** (33.5 mg, 100 µmol). It was obtained as a colorless solid (34 mg, 83%).

LC-MS (ESI): m/z = 433.2 [M+Na]$^+$

^{1}H NMR (300 MHz, CD_3CN) δ 8.75 (s, 1H), 7.28 – 7.17 (m, 2H), 6.96 (d, *J* = 8.2 Hz, 1H), 5.12 (p, *J* = 2.3 Hz, 1H), 4.59 (s, 2H), 4.33 (s, 2H), 4.11 (s, 2H), 3.11 – 3.03 (m, 2H), 3.02 – 2.93 (m, 2H), 1.20 (s, 12H).

^{13}C NMR (75 MHz, CD_3CN) δ 169.49, 165.28, 162.86, 146.60, 128.55, 127.92, 124.46, 116.94, 116.53, 83.63, 68.02, 45.88, 45.53, 34.51, 25.14.

((2-(3-Oxo-3,4-dihydro-2*H*-benzo[b][1,4]oxazine-6-carbonyl)-2-azaspiro[3.3]heptan-6-ylidene)methyl)boronic acid (VIIg)

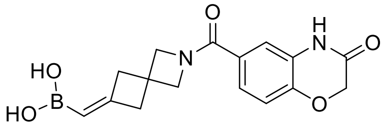

The title compound was synthesized from the intermediate **VIg** (30.8 mg, 75 µmol) according to GP5. It was obtained as a colorless amorphous solid (21 mg, 85%).

LC-MS (ESI): m/z = 329.1 [M+H]$^+$

^{1}H NMR (300 MHz, MeOD) δ 7.29 (dd, *J* = 8.4, 2.1 Hz, 1H), 7.23 (d, *J* = 2.0 Hz, 1H), 7.02 (d, *J* = 8.4 Hz, 1H), 5.17 – 5.13 (m, 1H), 4.65 (s, 2H), 4.41 (s, 2H), 4.19 – 4.17 (m, 2H), 3.13 – 3.09 (m, 2H), 3.02 – 2.96 (m, 2H).

6-(6-((5,5-Difluoro-5*H*-4λ⁴,5λ⁴-dipyrrolo[1,2-c:2',1'-f][1,3,2]diazaborinin-10-yl)methylene)-2-azaspiro[3.3]heptane-2-carbonyl)-2*H*-benzo[b][1,4]oxazin-3(4*H*)-one (VIIIg)

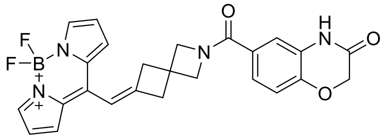

The title compound was synthesized according to GP6 from intermediate **VIIg** (11.5 mg, 35 µmol). It was obtained as an intensely red-colored amorphous solid (4.32 mg, 26%).

LC-MS (ESI): m/z = 455.1 $[M-F]^{+}$

1H NMR (600 MHz, CD_3CN) δ 8.66 – 8.61 (m, 1H), 7.82 (s, 2H), 7.37 (d, *J* = 4.2 Hz, 2H), 7.22 (dd, *J* = 8.4, 2.0 Hz, 1H), 7.17 (d, *J* = 2.0 Hz, 1H), 6.95 (d, *J* = 8.3 Hz, 1H), 6.71 (p, *J* = 2.2 Hz, 1H), 6.57 (dd, *J* = 4.4, 1.8 Hz, 2H), 4.58 (s, 2H), 4.39 – 4.28 (m, 2H), 4.19 – 4.06 (m, 2H), 3.19 (t, *J* = 2.3 Hz, 2H), 3.14 – 3.09 (m, 2H).

13C NMR (151 MHz, CD_3CN) δ 169.44, 165.28, 155.40, 146.60, 144.50, 143.83, 134.56, 130.58, 128.82, 127.94, 124.45, 118.90, 118.87, 116.97, 116.49, 68.07, 45.33, 45.29, 35.51.

6-(6-((5,5-Difluoro-5*H*-4λ⁴,5λ⁴-dipyrrolo[1,2-c:2',1'-f][1,3,2]diazaborinin-10-yl)methyl)-2-azaspiro[3.3]heptane-2-carbonyl)-2*H*-benzo[b][1,4]oxazin-3(4*H*)-one (IXg)

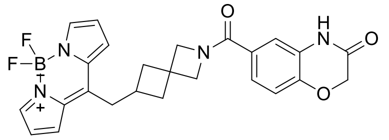

The title compound was synthesized according to GP7 from intermediate **VIIIg** (4.27 mg, 9 µmol). It was obtained as an amorphous orange solid (4.01 mg, 94%) with green fluorescence in solution.

HRMS (ESI) m/z $[M+H]^{+}$ calcd. for $C_{25}H_{23}BF_2N_4O_3$ 475.1868 found 475.1868

^{1}H NMR (600 MHz, CD_3CN) δ 8.63 (s, 1H), 7.85 (s, 2H), 7.49 (d, *J* = 4.2 Hz, 2H), 7.20 (d, *J* = 8.8 Hz, 1H), 7.16 (d, *J* = 1.9 Hz, 1H), 6.97 – 6.91 (m, 1H), 6.61 (dd, *J* = 4.8, 1.8 Hz, 2H), 4.58 (s, 2H), 4.30 – 4.16 (m, 2H), 4.08 – 3.95 (m, 2H), 3.64 – 3.55 (m, 1H), 3.07 (d, *J* = 7.5 Hz, 2H), 2.26 (ddd, *J* = 10.2, 7.8, 2.9 Hz, 2H), 1.95 (d, *J* = 2.6 Hz, 2H).

^{13}C NMR (151 MHz, CD_3CN) δ 169.35, 165.29, 150.84, 146.53, 144.46, 136.29, 130.14, 128.94, 127.91, 124.42, 119.17, 118.30, 116.91, 116.51, 68.07, 39.76, 37.48.

(4-(5-Methyloxazolo[4,5-b]pyridin-2-yl)piperazin-1-yl)(6-((4,4,5,5-tetramethyl-1,3,2-dioxaborolan-2-yl)methylene)-2-azaspiro[3.3]heptan-2-yl)methanone (VIk)

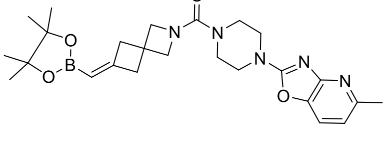

The title compound was synthesized according to GP1 A from **IVb** (67 mg, 200 µmol) and 5-methyl-2-(piperazin-1-yl)oxazolo[4,5-b]pyridine (43.7 mg, 200 µmol). Clean product was obtained as a colorless amorphous solid (67 mg, 70%) after liquid extraction.

LC-MS (ESI): m/z = 502.2 $[M+Na]^{+}$

^{1}H NMR (300 MHz, CD_3CN) δ 7.89 (d, *J* = 8.0 Hz, 1H), 7.11 – 7.02 (m, 1H), 5.09 (dt, *J* = 13.7, 2.3 Hz, 1H), 4.04 (t, *J* = 2.1 Hz, 4H), 3.82 – 3.72 (m, 4H), 3.54 – 3.44 (m, 4H), 3.04 (q, *J* = 2.5 Hz, 2H), 2.97 – 2.85 (m, 2H), 2.64 (s, 3H) 1.20 (s, 12H).

^{13}C NMR (75 MHz, CD_3CN) δ 166.58, 162.98, 162.87, 159.23, 155.22, 145.85, 144.11, 122.23, 119.10, 116.37, 115.25, 83.62, 63.99, 63.88, 46.46, 45.92, 45.86, 45.60, 45.46, 44.40, 34.41, 34.28, 25.16, 19.30.

((2-(4-(5-Methyloxazolo[4,5-b]pyridin-2-yl)piperazine-1-carbonyl)-2-azaspiro[3.3]heptan-6-ylidene)methyl)boronic acid (VIIk)

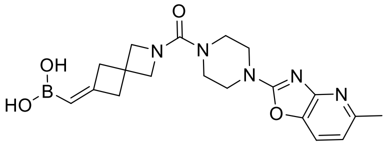

The title compound was synthesized according to GP5 from intermediate **VIk** (65 mg, 135 µmol) and obtained after RP-HPLC as a colorless amorphous solid (18 mg, 34%).

LC-MS (ESI): m/z = 398.2 [M+H]$^+$

^{1}H NMR (300 MHz, MeOD) δ 8.01 (d, *J* = 8.0 Hz, 1H), 7.15 (dd, *J* = 8.0, 0.8 Hz, 1H), 5.13 (t, *J* = 2.4 Hz, 1H), 4.09 (d, *J* = 2.1 Hz, 4H), 3.92 – 3.83 (m, 4H), 3.60 – 3.48 (m, 4H), 3.09 (dd, *J* = 8.4, 2.6 Hz, 2H), 3.02 – 2.95 (m, 2H), 2.64 (d, *J* = 0.8 Hz, 3H).

^{13}C NMR (75 MHz, CD_3CN) δ 166.33, 162.94, 159.68, 155.90, 147.30, 143.49, 121.07, 116.18, 63.79, 46.41, 45.92, 45.40, 44.42, 34.41, 20.08.

(6-((5,5-Difluoro-5*H*-4λ⁴,5λ⁴-dipyrrolo[1,2-c:2',1'-f][1,3,2]diazaborinin-10-yl)methylene)-2-azaspiro[3.3]heptan-2-yl)(4-(5-methyloxazolo[4,5-b]pyridin-2-yl)piperazin-1-yl)methanone (VIIIk)

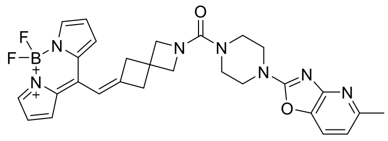

The title compound was synthesized according to GP6 from intermediate **VIIk** (18 mg, 45 µmol). The product was obtained as an intensely red-colored amorphous solid (5 mg, 20%).

HRMS (ESI) m/z [M+H]$^+$ calcd. for $C_{28}H_{28}BF_2N_7O_2$ 542.2402 found 542.2417

^{1}H NMR (300 MHz, CD_3CN) δ 7.86 – 7.78 (m, 2H), 7.77 (d, *J* = 8.0 Hz, 1H), 7.37 (d, *J* = 4.3 Hz, 1H), 7.00 (dd, *J* = 8.0, 0.8 Hz, 1H), 6.71 (p, *J* = 2.2 Hz, 1H), 6.61 – 6.53 (m, 2H), 4.08 – 3.94 (m, 4H), 3.76 – 3.69 (m, 4H), 3.47 – 3.40 (m, 4H), 3.16 (d, *J* = 2.0 Hz, 2H), 3.09 (d, *J* = 2.5 Hz, 2H), 2.60 (s, 3H).

^{13}C NMR (75 MHz, CD_3CN) δ 166.26, 162.83, 155.95, 155.70, 147.60, 144.49, 143.78, 143.39, 134.50, 134.46, 130.57, 130.55, 130.52, 120.90, 116.86, 116.19, 63.11, 46.36, 45.38, 45.34, 44.40, 35.27, 20.17.

(6-((5,5-Difluoro-5*H*-4λ⁴,5λ⁴-dipyrrolo[1,2-c:2',1'-f][1,3,2]diazaborinin-10-yl)methyl)-2-azaspiro[3.3]heptan-2-yl)(4-(5-methyloxazolo[4,5-b]pyridin-2-yl)piperazin-1-yl)methanone (S20 IXk)

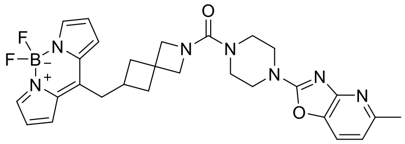

The title compound was synthesized according to GP7 from intermediate **VIIIk** (5 mg, 9.2 µmol). It was obtained as an amorphous orange solid with green fluorescence in solution (3 mg, 60%).

HRMS (ESI) m/z [M+H]$^+$ calcd. for $C_{28}H_{30}BF_2N_7O_2$ 544.2558 found 544.2580

^{1}H NMR (600 MHz, CD_3CN) δ 7.86 (d, *J* = 2.4 Hz, 2H), 7.74 – 7.69 (m, 1H), 7.49 (d, *J* = 4.3 Hz, 2H), 6.97 (d, *J* = 8.1 Hz, 1H), 6.61 (dd, *J* = 4.4, 2.1 Hz, 2H), 3.94 (s, 2H), 3.88 (s, 2H), 3.73 – 3.68 (m, 4H), 3.43 –

3.39 (m, 4H), 3.07 (d, *J* = 7.5 Hz, 2H), 2.58 (s, 3H), 2.49 (h, *J* = 8.2 Hz, 1H), 2.27 – 2.20 (m, 2H), 2.01 (td, *J* = 8.9, 2.9 Hz, 2H).

1,1,1,3,3,3-Hexafluoropropan-2-yl 6-((4,4,5,5-tetramethyl-1,3,2-dioxaborolan-2-yl)methylene)-2-azaspiro[3.3]heptane-2-carboxylate (VIl)

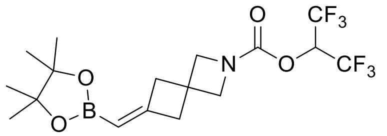

This intermediate was synthesized by first deprotecting intermediate **IVb** (67 mg, 200 µmol) as described in GP1. The deprotected amine was taken up in 10 mL anhydrous DCM, and DIPEA (45 µl, 260 µmol) was added. The mixture is cooled to 0 °C before bis(1,1,1,3,3,3-hexafluoropropan-2-yl) carbonate ~50 wt% in Et_2O (1 equiv., freshly prepared according to literature[18]) solution was added. The mixture was allowed to warm to r.t. and continued to stir for 3 h. The mixture was diluted with 10 mL DCM and washed with saturated NaCl solution (5mL). The organic layer was dried over $MgSO_4$, filtrated, and adsorbed onto silica before being purified by silica gel chromatography with 0-20% EtOAc in cyclohexane (ELSD recommended). The product was obtained as a colorless solid (67 mg, 78%).

LC-MS (ESI): m/z = 452.1 $[M+Na]^+$

^{1}H NMR (300 MHz, $CDCl_3$) δ 5.64 (hept, *J* = 6.2 Hz, 1H), 5.22 (p, *J* = 2.3 Hz, 1H), 4.20 – 4.06 (m, 4H), 3.14 (t, *J* = 2.5 Hz, 2H), 3.00 – 2.97 (m, 2H), 1.23 (s, 12H).

^{13}C NMR (75 MHz, $CDCl_3$) δ 160.18, 151.65, 126.76 – 114.89 (m), 82.97, 67.68 (p, *J* = 34.5 Hz), 62.04, 61.30, 45.29, 45.16, 34.11, 24.98, 24.85.

((2-(((1,1,1,3,3,3-Hexafluoropropan-2-yl)oxy)carbonyl)-2-azaspiro[3.3]heptan-6-ylidene)methyl)boronic acid (VIIl)

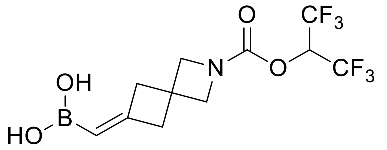

The title compound was synthesized according to GP5 from intermediate **VIl** (66.5 mg, 155 µmol). The product was obtained after liquid extraction as an amorphous colorless solid (51 mg, 95%).

LC-MS (ESI): m/z = 348.1 $[M+H]^+$

^{1}H NMR (300 MHz, MeOD) δ 6.02 (hept, *J* = 6.4 Hz, 1H), 5.34 (p, *J* = 2.3 Hz, 1H), 4.15 (s, 2H), 4.09 (s, 2H), 3.11 (p, *J* = 2.5 Hz, 2H), 3.01 (d, *J* = 2.2 Hz, 2H).

^{13}C NMR (75 MHz, MeOD) δ 160.32, 153.03, 68.68 (p, *J* = 34.2 Hz), 62.80, 62.13, 45.93, 45.80, 35.29.

^{19}F NMR (282 MHz, MeOD) δ 2.22.

1,1,1,3,3,3-Hexafluoropropan-2-yl 6-((5,5-difluoro-5*H*-4λ⁴,5λ⁴-dipyrrolo[1,2-c:2',1'-f][1,3,2]diazaborinin-10-yl)methylene)-2-azaspiro[3.3]heptane-2-carboxylate (VIIIl)

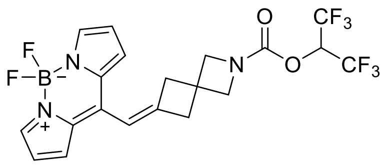

The title compound was synthesized according to GP6 from intermediate **VIIl** (50.3 mg, 145 µmol). The title compound was obtained as an intensely red-colored amorphous solid (26 mg, 36%).

LC-MS (ESI): m/z = 474.1 $[M-F]^+$

^{1}H NMR (300 MHz, CD_3CN) δ 7.82 (s, 2H), 7.36 (d, *J* = 4.3 Hz, 2H), 6.71 (p, *J* = 2.2 Hz, 1H), 6.60 – 6.54 (m, 2H), 5.90 (hept, *J* = 6.3 Hz, 1H), 4.09 (dt, *J* = 23.1, 6.8 Hz, 4H), 3.19 (t, *J* = 2.1 Hz, 2H), 3.12 (t, *J* = 2.4 Hz, 2H).

^{13}C NMR (75 MHz, CD_3CN) δ 154.91, 152.31, 144.37, 143.81, 134.48, 130.55, 118.83, 117.05, 61.88, 61.21, 44.99, 35.53.

^{19}F NMR (282 MHz, CD_3CN) δ 3.04, -68.12 (dd, *J* = 56.9, 28.5 Hz).

1,1,1,3,3,3-Hexafluoropropan-2-yl 6-((5,5-difluoro-5*H*-4λ⁴,5λ⁴-dipyrrolo[1,2-c:2',1'-f][1,3,2]diazaborinin-10-yl)methyl)-2-azaspiro[3.3]heptane-2-carboxylate (2 IXl)

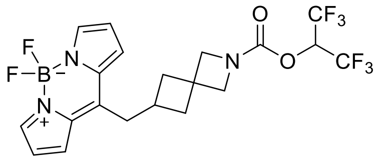

The title compound was synthesized according to GP7 from intermediate **VIIIl** (26 mg, 53 μmol). It was obtained as an amorphous orange solid with green fluorescence in solution (23 mg, 88%).

HRMS (ESI) m/z $[M+H]^+$ calcd. for $C_{20}H_{18}BF_8N_3O_2$ 494.1401 found 494.1412

^{1}H NMR (600 MHz, CD_3CN) δ 7.85 (s, 2H), 7.48 (d, *J* = 4.3 Hz, 2H), 6.63 – 6.59 (m, 2H), 5.88 (hept, *J* = 6.4 Hz, 1H), 4.09 – 3.92 (m, 4H), 3.06 (d, *J* = 7.5 Hz, 2H), 2.50 (hept, *J* = 8.0 Hz, 1H), 2.28 – 2.23 (m, 2H), 2.05 – 2.00 (m, 2H).

^{13}C NMR (151 MHz, CD_3CN) δ ^{13}C NMR (151 MHz, CD_3CN) δ 150.74, 144.49, 136.28, 130.14, 119.19, 68.27 (d, *J* = 33.7 Hz), 62.33 (dd, *J* = 264.8, 102.0 Hz), 39.49, 37.43, 35.50, 34.31, 30.38.

^{19}F NMR (282 MHz, CD_3CN) δ 3.01 (d, *J* = 3.8 Hz), -67.77 (dd, *J* = 57.2, 29.0 Hz).

(*R*)-1,1,1-Trifluoro-3-((4-methoxybenzyl)oxy)propan-2-ol

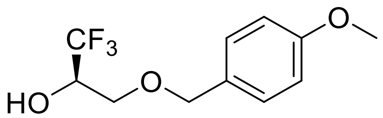

(4-Methoxyphenyl)methanol (777 mg, 5.625 mmol, 1.5 equiv.) was slowly added to a 0 °C solution of NaH (60 wt% in mineral oil, 450 mg, 11.25 mmol, 3 equiv.) in tetrahydrofuran (10 mL). After the reaction mixture had stirred at 0 °C for 60 minutes, then (2R)-2-(trifluoromethyl)oxirane (prepared by hydrolytic kinetic resolution from racemate [19], 420 mg, 3.75 mmol) in 2 mL THF was added and allowed to warm to ambient temperature and stirred for 40 h. The reaction mixture was then cooled to 0 °C and quenched via the addition of water. The solution was separated between water and EtOAc. The aqueous phase was extracted with EtOAc two more times, the combined organic phase was dried over sodium sulfate anhydrous, filtrated and concentrated under reduced pressure. The title compound was purified via flash chromatography with gradient elution of 0-20% EtOAc in cyclohexane. The product was obtained as a pale-yellow oil (635 mg, 68%). Enantiomeric purity was confirmed via chiral HPLC.

LC-MS (ESI): m/z = 251.1 $[M+H]^+$

^{1}H NMR (300 MHz, $CDCl_3$) δ 7.30 – 7.21 (m, 2H), 6.95 – 6.85 (m, 2H), 4.53 (d, *J* = 1.2 Hz, 2H), 4.12 (d, *J* = 5.7 Hz, 1H), 3.82 (s, 3H), 3.76 – 3.57 (m, 2H), 2.88 (s, 1H).

^{13}C NMR (75 MHz, $CDCl_3$) δ 159.58, 129.55, 129.05, 124.29 (q, *J* = 281.9 Hz) 114.00, 73.46, 69.56 (q, *J* = 31.0 Hz), 67.07, 67.04, 55.32.

(*R*)-1,1,1-Trifluoro-3-((4-methoxybenzyl)oxy)propan-2-yl 6-((4,4,5,5-tetramethyl-1,3,2-dioxaborolan-2-yl)methylene)-2-azaspiro[3.3]heptane-2-carboxylate (VIm)

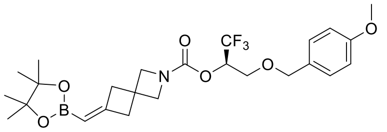

Intermediate **IVb** (80.4 mg, 240 μmol) was *N*-Boc deprotected as described in GP1.

To a solution of (R)-1,1,1-trifluoro-3-((4-methoxybenzyl)oxy)propan-2-ol (72 mg, 288 μmol) in anhydrous acetonitrile (2 mL) were added triethylamine (200 μl, 1.44 mmol) and bis(pentafluorophenyl)carbonate (136 mg, 346 μmol). The reaction mixture was stirred for 1 h at 0 °C and then allowed to warm to rt. It was then treated with a solution of previously boc deprotected **IVb** and triethylamine (167 μL, 1.2 mmol) in anhydrous acetonitrile (2 mL). The reaction mixture was stirred at rt for 16 h, whereupon it was concentrated in vacuo. Purification of the crude via silica gel chromatography with gradient elution of 0-20 % EtOAc in cyclohexane. The title compound was obtained as a colorless amorphous solid (102 mg, 82%).

HRMS (ESI) m/z $[M+H]^+$ calcd. for $C_{25}H_{33}BF_3NO_6$ 510.2389 found 510.2405

^{1}H NMR (300 MHz, $CDCl_3$) δ 7.25 (d, *J* = 8.9 Hz, 2H), 6.93 – 6.84 (m, 2H), 5.38 (qd, *J* = 7.1, 4.0 Hz, 1H), 5.21 (t, *J* = 2.3 Hz, 1H), 4.50 (q, *J* = 11.7 Hz, 2H), 4.19 – 3.99 (m, 4H), 3.81 (s, 3H), 3.69 (qd, *J* = 11.2, 5.5 Hz, 2H), 3.11 (d, *J* = 2.5 Hz, 2H), 2.96 (s, 2H), 1.24 (s, 12H).

^{13}C NMR (75 MHz, $CDCl_3$) δ 160.88, 159.53, 154.09, 129.53, 129.43, 114.00, 82.99, 73.04, 69.83, 69.40, 66.19, 55.43, 45.40, 45.16, 33.88, 27.06, 24.99.

(*R*)-((2-(((1,1,1-Trifluoro-3-((4-methoxybenzyl)oxy)propan-2-yl)oxy)carbonyl)-2-azaspiro[3.3]heptan-6-ylidene)methyl)boronic acid (VIIm)

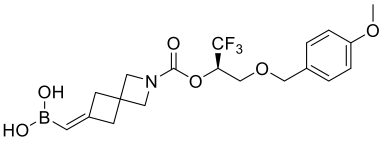

The title compound was obtained from intermediate **VIm** (102 mg, 200 μmol) according to GP5. The crude product which was obtained after liquid extraction (H_2O/EtOAc) was used without further purification.

LC-MS (ESI): m/z = 430.2 $[M+H]^+$

(*R*)-1,1,1-Trifluoro-3-((4-methoxybenzyl)oxy)propan-2-yl 6-((5,5-difluoro-5*H*-4λ⁴,5λ⁴-dipyrrolo[1,2-c:2',1'-f][1,3,2]diazaborinin-10-yl)methylene)-2-azaspiro[3.3]heptane-2-carboxylate (VIIIm)

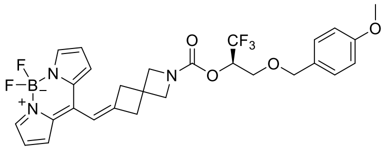

The title compound was synthesized from crude **VIIm** (32 mg, ~80% purity, ~ 67.5 μmol, 1.5 equiv.) and 8-(methylthio)-4,4,difluoro-4-bora-3a,4a-diaza-s-indacene (10.7 mg, 45 μmol) according to GP6. The title compound was obtained after RP-HPLC as an intensely red-colored amorphous solid (23 mg, 89%).

HRMS (ESI) m/z $[M+H]^+$ calcd. for $C_{28}H_{27}BF_5N_3O_4$ 574.2051 found 574.2038

^{1}H NMR (300 MHz, $CDCl_3$) δ 7.84 (s, 2H), 7.25 – 7.19 (m, 2H), 7.16 (d, *J* = 4.2 Hz, 2H), 6.88 (s, 2H), 6.56 (tt, *J* = 2.2, 1.3 Hz, 1H), 6.51 (dd, *J* = 4.2, 1.7 Hz, 2H), 5.39 (pd, *J* = 7.0, 3.8 Hz, 1H), 4.49 (q, *J* = 11.7 Hz, 2H), 4.16 – 4.01 (m, 4H), 3.79 (s, 3H), 3.70 (td, *J* = 12.8, 5.8 Hz, 2H), 3.16 (s, 2H), 3.07 (s, 2H).

^{13}C NMR (75 MHz, CD_3CN) δ 159.44, 154.22, 153.84, 143.44, 142.83, 133.67, 133.52, 133.10, 129.77, 129.54, 128.15, 117.89, 116.02, 113.70, 72.59, 69.36 (q, *J* = 30.9 Hz), 65.99, 60.01, 54.89, 44.09, 34.42, 26.62.

(*R*)-1,1,1-Trifluoro-3-hydroxypropan-2-yl 6-((5,5-difluoro-5*H*-4λ⁴,5λ⁴-dipyrrolo[1,2-c:2',1'-f][1,3,2]diazaborinin-10-yl)methylene)-2-azaspiro[3.3]heptane-2-carboxylate (VIIIm deprotected)

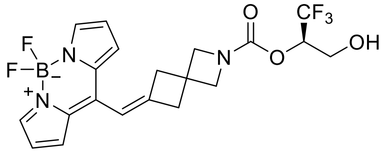

Intermediate **VIIIm** (11.5 mg, 20 µmol) was dissolved in DCM (2 mL), H_2O (0.1 mL), and DDQ (6.8 mg, 30 µmol) were added. The mixture was vigorously stirred at ambient temperature for 18 h. The mixture is separated between saturated sodium hydrogen carbonate solution and DCM. The aqueous layer was extracted twice with DCM, and the combined organic phase was dried over anhydrous magnesium sulfate, filtrated, and concentrated under reduced pressure. The crude product was purified via RP-HPLC to give the title compound as an intensely red-colored amorphous solid (5.42 mg, 60%).

LC-MS (ESI): m/z = 436.1 $[M-F]^+$

^1H NMR (300 MHz, CD_3CN) δ 7.82 (s, 2H), 7.36 (d, *J* = 4.2 Hz, 2H), 6.71 (p, *J* = 2.2 Hz, 1H), 6.57 (dd, *J* = 4.3, 1.7 Hz, 2H), 5.17 (pd, *J* = 7.2, 3.9 Hz, 1H), 4.15 – 3.94 (m, 4H), 3.80 (dd, *J* = 12.4, 4.0 Hz, 1H), 3.69 (dd, *J* = 12.4, 6.8 Hz, 1H), 3.18 (q, *J* = 2.7 Hz, 2H), 3.10 (q, *J* = 2.7 Hz, 2H).

^{13}C NMR (75 MHz, CD_3CN) δ 155.25, 155.02, 144.42, 143.80, 134.48, 130.57, 118.88, 118.84, 116.98, 72.74 – 70.93 (m), 59.54, 45.09, 35.39.

^{19}F NMR (282 MHz, CD_3CN) δ 1.58, -68.09 (dd, *J* = 56.8, 28.6 Hz).

(*R*)-1,1,1-Trifluoro-3-hydroxypropan-2-yl 6-((5,5-difluoro-5*H*-4λ⁴,5λ⁴-dipyrrolo[1,2-c:2',1'-f][1,3,2]diazaborinin-10-yl)methyl)-2-azaspiro[3.3]heptane-2-carboxylate (1 IXm)

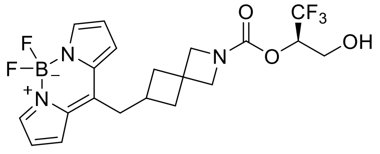

The title compound was synthesized from intermediate **VIIIm** (5.4 mg, 12 µmol) according to GP 7. The product was obtained after RP-HPLC as an amorphous orange solid with green fluorescence in solution (5.4 mg, quantitative yield). Compound IXm could also be obtained with a similar total yield, if the order of the last two steps, PMB deprotection with DDQ and hydrogenation of the double bond, was switched. However, achieving both in one step under harsher hydrogenation conditions was not successful.[1]

HRMS (ESI) m/z $[M+H]^+$ calcd. for $C_{20}H_{21}BF_5N_3O_3$ 456.1632 found 456.1637

^1H NMR (300 MHz, CD_3CN) δ 7.85 (s, 2H), 7.49 (d, *J* = 4.3 Hz, 2H), 6.65 – 6.57 (m, 3H), 5.16 (pd, *J* = 7.2, 3.9 Hz, 1H), 4.07 – 3.85 (m, 4H), 3.85 – 3.62 (m, 2H), 3.05 (d, *J* = 7.5 Hz, 2H), 2.49 (tt, *J* = 8.9, 7.3 Hz, 1H), 2.30 – 2.16 (m, 1H), 2.12 – 1.95 (m, 2H).

^{13}C NMR (75 MHz, CD_3CN) δ 154.97, 144.45, 136.24, 130.11, 119.15, 118.70, 72.92 – 71.37 (m), 63.36, 59.55, 39.55, 37.43, 35.26, 34.37.

[1] Compound **1** could also be obtained with a similar total yield, if the order of the last two steps, PMB deprotection with DDQ and hydrogenation of the double bond, was switched. However, achieving both in one step under harsher hydrogenation conditions was not successful.

5,5-Difluoro-10-(methylthio)-3-phenyl-5*H*-5λ[4],6λ[4]-dipyrrolo[1,2-c:2',1'-f][1,3,2]diazaborinine

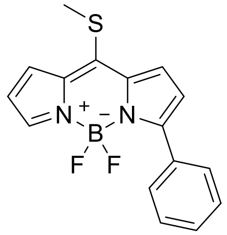

An oven-dried flask equipped with a stir bar was charged with 8-(methylthio)-4,4,difluoro-4-bora-3a,4a-diaza-s-indacene (71.4 mg, 300 μmol), aniline (55 μL, 600 μmol) and anhydrous acetonitrile (20 mL). The mixture was stirred until the solids dissolved, then ^{t}BuONO (107 μL, 900 μmol) was added via a syringe. A fine bubbling was observed. The reaction was warmed to 40 °C for 16 h. The solvent was removed under reduced pressure, and the crude material was purified via RP-HPLC to give the title compound as an intensely red-colored amorphous solid (18.6 mg, 20%). A small amount of 3,5-bis-phenyl substituted analog could additionally be isolated.

HRMS (ESI) m/z $[M+H]^+$ calcd. for $C_{16}H_{13}BF_2N_2S$ 313.0897 found 313.0908

^{1}H NMR (300 MHz, $CDCl_3$) δ 7.91 (dd, *J* = 3.3, 1.3 Hz, 1H), 7.88 (d, *J* = 1.8 Hz, 1H), 7.76 – 7.68 (m, 1H), 7.56 (d, *J* = 4.4 Hz, 1H), 7.52 – 7.44 (m, 3H), 7.37 (d, *J* = 3.8 Hz, 1H), 6.67 (d, *J* = 4.1 Hz, 1H), 6.54 – 6.50 (m, 1H), 2.87 (s, 3H).

(4a*R*,8a*S*)-6-(6-((5,5-Difluoro-3-phenyl-5*H*-4λ[4],5λ[4]-dipyrrolo[1,2-c:2',1'-f][1,3,2]diazaborinin-10-yl)methylene)-2-azaspiro[3.3]heptane-2-carbonyl)hexahydro-2*H*-pyrido[4,3-b][1,4]oxazin-3(4H)-one (VIIIn)

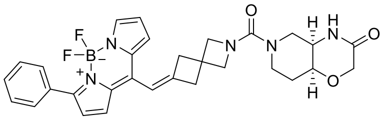

The title compound was synthesized according to GP6 from intermediate **VIb** (10.1 mg, 30 μmol) and 5,5-difluoro-10-(methylthio)-3-phenyl-5*H*-5λ[4],6λ[4]-dipyrrolo[1,2-c:2',1'-f][1,3,2]-diazaborinine (9.4 mg, 30 μmol). It was obtained after RP-HPLC purification as an intensely purple-red amorphous solid (16.5 mg, 99%).

LC-MS (ESI): m/z = 538.2 $[M-F]^+$

^{1}H NMR (300 MHz, CD_3CN) δ 7.95 – 7.86 (m, 2H), 7.73 (s, 1H), 7.51 – 7.47 (m, 3H), 7.45 (d, *J* = 4.4 Hz, 1H), 7.29 (d, *J* = 4.2 Hz, 1H), 6.81 – 6.73 (m, 2H), 6.70 (t, *J* = 2.2 Hz, 1H), 6.59 – 6.51 (m, 1H), 4.21 – 3.90 (m, 7H), 3.77 (dd, *J* = 13.0, 4.8 Hz, 1H), 3.57 – 3.48 (m, 1H), 3.34 – 3.24 (m, 1H), 3.18 – 3.13 (m, 2H), 3.08 – 3.04 (m, 2H), 2.92 (dt, *J* = 12.7, 9.9 Hz, 2H), 1.88 – 1.70 (m, 2H).

^{13}C NMR (75 MHz, CD_3CN) δ 169.23, 162.76, 159.63, 154.51, 142.96, 142.22, 136.69, 133.69, 133.31, 131.89, 130.81, 130.30, 130.25, 130.20, 129.29, 128.92, 121.23, 116.89, 70.14, 67.99, 63.32, 63.29, 50.11, 46.78, 45.16, 40.23, 35.13, 30.05.

(4a*R*,8a*S*)-6-(6-((5,5-Difluoro-3-phenyl-5*H*-4λ[4],5λ[4]-dipyrrolo[1,2-c:2',1'-f][1,3,2]diazaborinin-10-yl)methyl)-2-azaspiro[3.3]heptane-2-carbonyl)hexahydro-2*H*-pyrido[4,3-b][1,4]oxazin-3(4H)-one (S22 IXn)

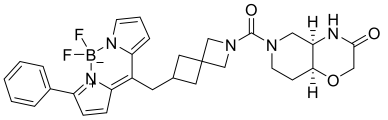

The title compound was synthesized according to GP7 from **VIIIn** (16.5 mg, 30 μmol). It was obtained after RP-HPLC purification as an amorphous red solid with orange fluorescence in solution (8 mg, 49%).

HRMS (ESI) m/z $[M+H]^+$ calcd. for $C_{30}H_{32}BF_2N_5O_3$ 558.2603 found 558.2602

^{1}H NMR (300 MHz, CD_3CN) δ 7.96 – 7.84 (m, 2H), 7.77 (s, 1H), 7.59 (d, *J* = 4.5 Hz, 1H), 7.54 – 7.45 (m, 3H), 7.42 (d, *J* = 4.3 Hz, 1H), 6.79 (d, *J* = 4.4 Hz, 1H), 6.80 – 6.67 (m, 1H), 6.59 (dd, *J* = 4.3, 1.9 Hz, 1H), 4.19 – 3.80 (m, 7H), 3.75 (dd, *J* = 13.1, 4.5 Hz, 1H), 3.58 – 3.45 (m, 1H), 3.35 – 3.22 (m, 1H), 3.07 (d, *J* = 7.4 Hz, 2H), 3.00 – 2.81 (m, 2H), 2.50 (hept, *J* = 7.3 Hz, 1H), 2.31 – 2.17 (m, 2H), 2.01 (td, *J* = 9.1, 2.9 Hz, 2H), 1.88 – 1.67 (m, 2H).

^{13}C NMR (75 MHz, CD_3CN) δ 169.25, 162.78, 160.11, 148.91, 142.79, 138.23, 135.44, 133.22, 131.22, 130.87, 130.36, 130.31, 130.25, 129.29, 128.56, 121.55, 70.14, 67.98, 65.00, 63.36, 50.11, 46.79, 40.23, 39.81, 37.33, 35.00, 34.52, 30.05.

^{19}F NMR (282 MHz, CD_3CN) δ 0.71, -61.64 (dd, *J* = 60.6, 30.2 Hz).

5,5-Difluoro-10-(methylthio)-3-(1*H*-pyrrol-2-yl)-5*H*-5λ⁴,6λ⁴-dipyrrolo[1,2-c:2',1'-f][1,3,2]diazaborinine

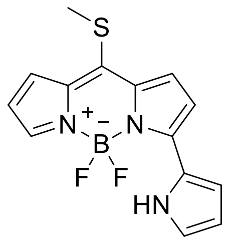

8-(methylthio)-4,4,difluoro-4-bora-3a,4a-diaza-s-indacene (71.4 mg, 300 µmol) was dissolved in 2.5 mL freshly distilled pyrrole in a 10 mL closed microwave vial under air and heated by MW radiation for 60 minutes to 150 °C, then the vial was opened to air and the reaction status was checked via LC-MS. The process was repeated until LC-MS indicated the formation of 3,8-Bis(pyrro-2-yl)BODIPY (m/z = 323 $[M+H]^+$) after 2 to 3 hours. The mixture was concentrated under reduced pressure, and the resulting residue was purified via RP-HPLC 50 to 95% ACN:$H_2$0 (+0.1% TFA). The title compound was isolated as an intensely violet-colored amorphous solid (8mg, 9%).

HRMS (ESI) m/z $[M+H]^+$ calcd. for $C_{14}H_{12}BF_2N_3S$ 302.0849 found 302.0843

^{1}H NMR (300 MHz, $CDCl_3$) δ 10.51 (s, 1H), 7.62 – 7.54 (m, 2H), 7.23 – 7.19 (m, 2H), 7.18 (d, *J* = 4.1 Hz, 1H), 7.10 – 7.02 (m, 1H), 6.93 (d, *J* = 4.8 Hz, 1H), 6.47 (t, *J* = 3.2 Hz, 1H), 6.43 – 6.39 (m, 1H), 2.70 (s, 3H).

^{13}C NMR (75 MHz, $CDCl_3$) δ 151.81, 140.13, 139.33, 135.87, 133.86, 131.64, 127.08, 123.59, 123.27, 121.41, 119.41, 115.90, 112.08, 21.73.

^{19}F NMR (282 MHz, $CDCl_3$) δ -63.86 (dd, *J* = 68.3, 34.0 Hz).

(4a*R*,8a*S*)-6-(6-((5,5-Difluoro-3-(1*H*-pyrrol-2-yl)-5*H*-4λ⁴,5λ⁴-dipyrrolo[1,2-c:2',1'-f][1,3,2]diazaborinin-10-yl)methylene)-2-azaspiro[3.3]heptane-2-carbonyl)hexahydro-2*H*-pyrido[4,3-b][1,4]oxazin-3(4H)-one (VIIIo)

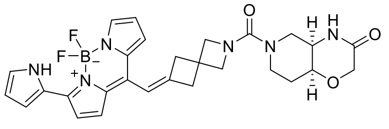

The title compound was synthesized according to GP6 from intermediate **VIb** (10.1 mg, 30 µmol), and 5,5-difluoro-10-(methylthio)-3-(1*H*-pyrrol-2-yl)-5*H*-5λ⁴,6λ⁴-dipyrrolo[1,2-c:2',1'-f][1,3,2]diazaborinine (9.1 mg, 30 µmol). It was obtained after RP-HPLC purification as an intensely purple-colored amorphous solid (6.93 mg, 42%).

LC-MS (ESI): m/z = 547.2 $[M+H]^+$

^{1}H NMR (300 MHz, CD_3CN) δ 10.44 (s, 1H), 7.55 (dd, *J* = 2.2, 1.2 Hz, 1H), 7.40 (d, *J* = 4.8 Hz, 1H), 7.27 (td, *J* = 2.8, 1.4 Hz, 1H), 7.18 (ddd, *J* = 4.0, 2.5, 1.4 Hz, 1H), 7.05 (d, *J* = 4.8 Hz, 1H), 7.02 (d, *J* = 1.1 Hz, 1H), 6.73 – 6.68 (m, 1H), 6.56 (p, *J* = 2.4 Hz, 1H), 6.47 (dd, *J* = 3.9, 2.2 Hz, 1H), 6.40 (dt, *J* = 4.4, 2.4 Hz,

1H), 4.18 – 3.91 (m, 7H), 3.76 (dd, *J* = 12.7, 5.3 Hz, 1H), 3.56 – 3.48 (m, 1H), 3.33 – 3.24 (m, 1H), 3.14 – 3.08 (m, 2H), 3.00 – 2.84 (m, 4H), 1.86 – 1.71 (m, 2H).

^{13}C NMR (75 MHz, CD_3CN) δ 169.14, 162.78, 152.09, 151.03, 137.43, 136.49, 136.38, 133.20, 132.79, 128.05, 124.24, 123.83, 121.68, 119.78, 116.70, 116.37, 112.52, 70.16, 68.00, 63.39, 63.35, 50.11, 46.79, 44.81, 44.77, 40.22, 35.04, 30.06.

^{19}F NMR (282 MHz, CD_3CN) δ 0.72, -64.55 (dd, *J* = 66.3, 32.8 Hz).

(4a*R*,8a*S*)-6-(6-((5,5-Difluoro-3-(1*H*-pyrrol-2-yl)-5*H*-4λ⁴,5λ⁴-dipyrrolo[1,2-c:2',1'-f][1,3,2]diazaborinin-10-yl)methyl)-2-azaspiro[3.3]heptane-2-carbonyl)hexahydro-2*H*-pyrido[4,3-b][1,4]oxazin-3(4H)-one (4a IXo)

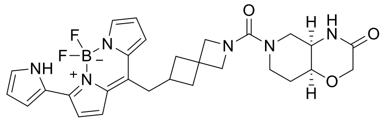

The title compound was synthesized according to GP7 from **VIIIo** (6.8 mg, 12.5 µmol). It was obtained after RP-HPLC purification as an amorphous, intensely purple-red solid with red fluorescence in solution (4.86 mg, 71%).

HRMS (ESI) m/z $[M+H]^+$ calcd. for $C_{28}H_{31}BF_2N_6O_3$ 547.2555 found 547.2554

^{1}H NMR (300 MHz, CD_3CN) δ 10.42 (s, 1H), 7.57 (s, 1H), 7.55 (d, *J* = 4.8 Hz, 1H), 7.27 (td, *J* = 2.8, 1.4 Hz, 1H), 7.18 (ddd, *J* = 4.0, 2.5, 1.4 Hz, 1H), 7.14 (d, *J* = 4.0 Hz, 1H), 7.07 (d, *J* = 4.8 Hz, 1H), 6.65 (s, 1H), 6.50 (dd, *J* = 3.9, 2.2 Hz, 1H), 6.39 (dt, *J* = 4.2, 2.4 Hz, 1H), 4.19 – 4.01 (m, 2H), 3.97 – 3.81 (m, 5H), 3.74 (dd, *J* = 13.0, 5.0 Hz, 1H), 3.57 – 3.45 (m, 1H), 3.28 (dt, *J* = 10.8, 3.7 Hz, 1H), 3.01 – 2.82 (m, 4H), 2.46 (dq, *J* = 15.9, 7.9 Hz, 1H), 2.27 – 2.15 (m, 2H), 2.05 – 1.97 (m, 2H), 1.87 – 1.66 (m, 2H).

^{13}C NMR (75 MHz, CD_3CN) δ 152.37, 141.75, 136.84, 134.31, 132.35, 127.94, 124.01, 123.48, 121.73, 119.72, 117.63, 116.58, 112.43, 70.19, 68.06, 64.97, 63.37, 50.13, 46.83, 40.19, 39.75, 36.89, 34.98, 33.99, 30.07.

^{19}F NMR (282 MHz, CD_3CN) δ 0.73, -64.58 (dd, *J* = 66.9, 32.9 Hz).

(6-(3-Cyclopropyl-1*H*-1,2,4-triazol-1-yl)-2-azaspiro[3.3]heptan-2-yl)(6-((5,5-difluoro-3-(1*H*-pyrrol-2-yl)-5*H*-4λ⁴,5λ⁴-dipyrrolo[1,2-c:2',1'-f][1,3,2]diazaborinin-10-yl)methylene)-2-azaspiro[3.3]heptan-2-yl)methanone (VIIIp)

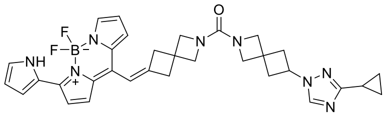

The title compound was synthesized according to GP6 from intermediate **VIIg** (10.3 mg, 27 µmol, 1.5 equiv.) and 5,5-difluoro-10-(methylthio)-3-(1*H*-pyrrol-2-yl)-5*H*-5λ⁴,6λ⁴-dipyrrolo[1,2-c:2',1'-f][1,3,2]diazaborinine (5.5 mg, 18 µmol, 1.0 equiv.). It was obtained after RP-HPLC as an intensely purple-colored amorphous solid (5.35 mg, 50%).

LC-MS (ESI): m/z = 595.2 $[M+H]^+$

^{1}H NMR (300 MHz, $CDCl_3$) δ 10.50 (s, 1H), 8.55 (s, 1H), 7.59 (s, 1H), 7.23 – 7.14 (m, 2H), 7.02 (tt, *J* = 2.4, 1.3 Hz, 1H), 6.90 (d, *J* = 4.7 Hz, 1H), 6.88 (dd, *J* = 3.8, 0.8 Hz, 1H), 6.48 – 6.43 (m, 2H), 6.39 (dt, *J* = 4.4, 2.4 Hz, 1H), 4.70 (p, *J* = 7.8 Hz, 1H), 4.09 – 3.96 (m, 8H), 3.11 (d, *J* = 2.1 Hz, 2H), 2.98 (d, *J* = 2.5 Hz, 2H), 2.77 (s, 2H), 2.75 (s, 2H), 2.11 (ddd, *J* = 8.3, 5.8, 3.3 Hz, 1H), 1.14 – 0.99 (m, 4H).

^{13}C NMR (75 MHz, $CDCl_3$) δ 162.34, 160.42, 146.91, 141.14, 136.69, 136.13, 131.38, 126.59, 123.81, 120.80, 118.67, 116.82, 115.57, 111.84, 77.36, 62.09, 61.79, 61.51, 49.88, 44.30, 44.17, 40.26, 34.80, 32.77, 8.92, 8.18.

^{19}F NMR (282 MHz, $CDCl_3$) δ 1.52, -64.03 (dd, *J* = 65.7, 30.8 Hz).

(6-(3-Cyclopropyl-1*H*-1,2,4-triazol-1-yl)-2-azaspiro[3.3]heptan-2-yl)(6-((5,5-difluoro-3-(1*H*-pyrrol-2-yl)-5*H*-4λ⁴,5λ⁴-dipyrrolo[1,2-c:2',1'-f][1,3,2]diazaborinin-10-yl)methyl)-2-azaspiro[3.3]heptan-2-yl)methanone (5a IXp)

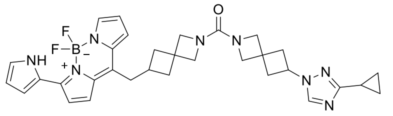

The title compound was synthesized according to GP7 from **VIIIp** (5.35 mg, 9 µmol) while using an increased amount of Pd/C (0.5 equiv.) and Et_3SiH (50 equiv.). It was obtained after RP-HPLC purification as an amorphous, intensely purple-red solid with red fluorescence in solution (4.43 mg, 83%).

HRMS (ESI) m/z $[M+H]^+$ calcd. for $C_{32}H_{35}BF_2N_8O$ 595.3031 found 595.3033

^1H NMR (600 MHz, $CDCl_3$) δ 10.49 (s, 1H), 8.32 (s, 1H), 7.61 (s, 1H), 7.27 (d, *J* = 5.1 Hz, 1H), 7.18 (td, *J* = 2.8, 1.4 Hz, 1H), 7.05 – 7.00 (m, 1H), 6.95 (d, *J* = 4.0 Hz, 1H), 6.93 (d, *J* = 4.7 Hz, 1H), 6.47 (dd, *J* = 3.9, 2.2 Hz, 1H), 6.39 (dt, *J* = 4.3, 2.3 Hz, 1H), 4.65 (p, *J* = 7.9 Hz, 1H), 4.02 (s, 2H), 3.99 (s, 2H), 3.93 (s, 2H), 3.86 (s, 2H), 2.90 (d, *J* = 7.3 Hz, 2H), 2.74 (d, *J* = 7.9 Hz, 4H), 2.50 (hept, *J* = 7.9 Hz, 1H), 2.29 (ddd, *J* = 10.4, 7.8, 2.9 Hz, 2H), 2.13 – 2.05 (m, 1H), 1.95 (td, *J* = 9.0, 2.9 Hz, 2H), 1.06 (dtd, *J* = 9.6, 6.3, 3.3 Hz, 2H), 1.01 (tt, *J* = 5.5, 3.0 Hz, 2H).

^{13}C NMR (151 MHz, $CDCl_3$) δ 162.45, 141.36, 138.02, 136.55, 130.10, 126.43, 123.61, 122.00, 120.88, 118.60, 115.78, 111.77, 63.18, 62.13, 61.56, 61.50, 49.57, 40.38, 39.53, 36.63, 34.87, 32.96, 32.78, 29.85, 8.62, 8.46.

1,1,1,3,3,3-Hexafluoropropan-2-yl 6-((5,5-difluoro-3-(1*H*-pyrrol-2-yl)-5*H*-4λ⁴,5λ⁴-dipyrrolo[1,2-c:2',1'-f][1,3,2]diazaborinin-10-yl)methylene)-2-azaspiro[3.3]heptane-2-carboxylate (VIIIq)

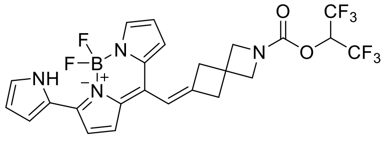

The title compound was synthesized according to GP6 from 5,5-difluoro-10-(methylthio)-3-(1*H*-pyrrol-2-yl)-5*H*-5λ⁴,6λ⁴-dipyrrolo[1,2-c:2',1'-f][1,3,2]diazaborinine (5.5 mg, 18 µmol) and intermediate **VIII** (7.9 mg, 19.8 µmol, 1.10 equiv.). It was obtained after silica gel flash chromatography with gradient elution of 0% to 15% EtOAc in cyclohexane as an intensely violet-colored amorphous solid (5.9 mg, 59%).

LC-MS (ESI): m/z = 581.2 $[M+Na]^+$

^1H NMR (300 MHz, $CDCl_3$) δ 10.50 (s, 1H), 7.59 (s, 1H), 7.21 – 7.09 (m, 2H), 7.02 (ddd, *J* = 3.9, 2.5, 1.4 Hz, 1H), 6.90 (d, *J* = 4.7 Hz, 1H), 6.87 (d, *J* = 4.0 Hz, 1H), 6.48 (t, *J* = 2.3 Hz, 1H), 6.44 (dd, *J* = 3.9, 2.2 Hz, 1H), 6.40 (dq, *J* = 4.2, 2.2 Hz, 1H), 5.63 (hept, *J* = 6.2 Hz, 1H), 4.26 – 4.06 (m, 4H), 3.17 (d, *J* = 2.3 Hz, 2H), 3.03 (d, *J* = 1.9 Hz, 2H).

^{13}C NMR (75 MHz, $CDCl_3$) δ 151.63, 146.05, 136.22, 134.86, 134.20, 133.79, 131.29, 128.16, 126.62, 123.80, 122.71, 120.86, 118.72, 117.08, 115.60, 111.85, 77.36, 61.59, 60.77, 44.09, 43.86, 34.82, 27.05.

1,1,1,3,3-Hexafluoropropan-2-yl 6-((5,5-difluoro-3-(1*H*-pyrrol-2-yl)-5*H*-4λ⁴,5λ⁴-dipyrrolo[1,2-c:2',1'-f][1,3,2]diazaborinin-10-yl)methyl)-2-azaspiro[3.3]heptane-2-carboxylate (2a IXq)

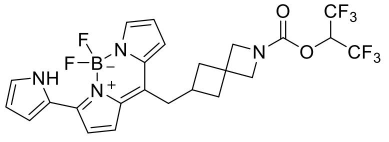

The title compound was synthesized according to GP7 from **VIIIq** (5.6 mg, 10 µmol). It was obtained after RP-HPLC purification as a purple-red amorphous solid with red fluorescence in solution (3 mg, 54%).

HRMS (ESI) m/z $[M+H]^+$ calcd. for $C_{24}H_{21}BF_8N_4O2$ 559.1666 found 559.1651

^{1}H NMR (300 MHz, CD_3CN) δ 10.42 (s, 1H), 7.58 (s, 1H), 7.55 (d, *J* = 4.8 Hz, 1H), 7.27 (q, *J* = 2.3 Hz, 1H), 7.18 (dt, *J* = 3.8, 1.2 Hz, 1H), 7.14 (d, *J* = 3.6 Hz, 1H), 7.08 (d, *J* = 4.8 Hz, 1H), 6.50 (dd, *J* = 3.9, 2.2 Hz, 1H), 6.39 (dt, *J* = 4.2, 2.3 Hz, 1H), 5.89 (hept, *J* = 6.2 Hz, 1H), 4.00 (t, *J* = 20.1 Hz, 4H), 2.97 (d, *J* = 7.4 Hz, 2H), 2.50 (hept, *J* = 7.8 Hz, 1H), 2.32 – 2.19 (m, 2H), 2.08 – 1.95 (m, 2H).

^{13}C NMR (75 MHz, CD_3CN) δ 151.32, 140.63, 137.93, 135.89, 133.34, 131.36, 126.99, 123.03, 122.49, 120.77, 118.78, 115.62, 111.47, 62.72 – 59.84 (m), 38.42, 35.86, 34.46, 32.81.

(*R*)-((2-(((1,1,1-Trifluoro-3-hydroxypropan-2-yl)oxy)carbonyl)-2-azaspiro[3.3]heptan-6-ylidene)methyl)boronic acid (VIIr)

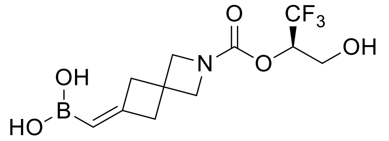

The title compound was synthesized by PMB deprotection of **VIm** (46 mg, 90 µmol) with DDQ as described in **VIIIm**. The crude residue after liquid-liquid extraction was subjected to Bpin hydrolytic conditions according to GP 5 without prior purification of the crude. The title compound was then isolated via RP-HPLC purification as a colorless amorphous solid (16.9 mg, 61% over 2 steps).

LC-MS (ESI): m/z = 310.1 $[M+H]^+$

^{1}H NMR (300 MHz, MeOD) δ 5.34 (p, *J* = 2.3 Hz, 1H), 5.21 (pd, *J* = 7.2, 3.8 Hz, 1H), 4.15 (s, 1H), 4.10 – 4.01 (m, 3H), 3.85 (dd, *J* = 12.4, 3.8 Hz, 1H), 3.73 (dd, *J* = 12.4, 7.2 Hz, 1H), 3.16 – 3.04 (m, 2H), 3.02 – 2.94 (m, 2H).

^{13}C NMR (75 MHz, MeOD) δ 159.22, 154.49, 131.22 – 117.75 (q), 71.43 (q, *J* = 30.4 Hz), 60.85 (d, *J* = 51.4 Hz), 58.34 (d, *J* = 2.1 Hz), 44.61, 44.47, 33.68.

(*R*)-1,1,1-Trifluoro-3-hydroxypropan-2-yl 6-((5,5-difluoro-3-(1*H*-pyrrol-2-yl)-5*H*-4λ⁴,5λ⁴-dipyrrolo[1,2-c:2',1'-f][1,3,2]diazaborinin-10-yl)methylene)-2-azaspiro[3.3]heptane-2-carboxylate (VIIIr)

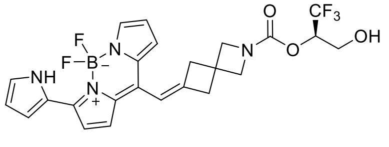

The title compound was synthesized according to GP6 from **VIIr** (10.7 mg, 34.5 µmol, 1.5 equiv.) and 5,5-difluoro-10-(methylthio)-3-(1*H*-pyrrol-2-yl)-5*H*-5λ⁴,6λ⁴-dipyrrolo[1,2-c:2',1'-f][1,3,2]diazaborinine (7 mg, 23 µmol). It was obtained after RP-HPLC purification as an intensely purple-colored amorphous solid (7.26 mg, 61%).

LC-MS (ESI): m/z = 521.1 $[M+H]^+$

^{1}H NMR (300 MHz, CD_3CN) δ 10.44 (s, 1H), 7.56 (d, *J* = 2.1 Hz, 1H), 7.41 (d, *J* = 4.8 Hz, 1H), 7.28 (td, *J* = 2.8, 1.4 Hz, 1H), 7.18 (ddd, *J* = 4.0, 2.6, 1.4 Hz, 1H), 7.05 (d, *J* = 4.8 Hz, 1H), 7.02 (d, *J* = 4.1 Hz, 1H),

6.57 (p, *J* = 2.1 Hz, 1H), 6.47 (dd, *J* = 4.0, 2.3 Hz, 1H), 6.40 (dt, *J* = 4.2, 2.4 Hz, 1H), 5.17 (pd, *J* = 7.2, 4.0 Hz, 1H), 4.10 – 3.99 (m, 4H), 3.80 (dd, *J* = 12.3, 3.9 Hz, 1H), 3.69 (dd, *J* = 12.4, 6.7 Hz, 1H), 3.15 (d, *J* = 2.2 Hz, 2H), 3.02 (d, *J* = 2.6 Hz, 2H).

^{13}C NMR (75 MHz, CD_3CN) δ 154.99, 152.09, 150.57, 149.02, 137.46, 136.40, 133.19, 132.79, 128.07, 124.23, 123.86, 121.70, 119.79, 116.84, 112.52, 112.05, 72.19, 71.79, 59.54, 44.60, 44.49, 35.31.

(*R*)-1,1,1-Trifluoro-3-hydroxypropan-2-yl 6-((5,5-difluoro-3-(1*H*-pyrrol-2-yl)-5*H*-4λ⁴,5λ⁴-dipyrrolo[1,2-c:2',1'-f][1,3,2]diazaborinin-10-yl)methyl)-2-azaspiro[3.3]heptane-2-carboxylate (1a IXr)

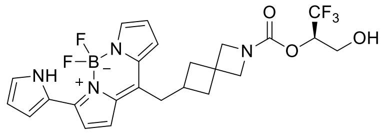

The title compound was synthesized according to GP7 from **VIIIr** (7.28 mg, 14 µmol). it was obtained after RP-HPLC as an amorphous, intensely purple-red solid with red fluorescence in solution (3.64 mg, 50%).

HRMS (ESI) m/z $[M+H]^+$ calcd. for $C_{24}H_{24}BF_5N_4O_3$ 521.1898 found 521.1951

^{1}H NMR (600 MHz, CD_3CN) δ 10.43 (s, 1H), 7.58 (s, 1H), 7.55 (d, *J* = 4.8 Hz, 1H), 7.27 (td, *J* = 2.8, 1.4 Hz, 1H), 7.19 – 7.17 (m, 1H), 7.15 (d, *J* = 3.9 Hz, 1H), 7.07 (d, *J* = 4.8 Hz, 1H), 6.50 (dd, *J* = 4.0, 2.2 Hz, 1H), 6.40 (dt, *J* = 4.4, 2.4 Hz, 1H), 5.16 (pd, *J* = 7.1, 3.9 Hz, 1H), 4.09 – 3.84 (m, 4H), 3.79 (dd, *J* = 12.3, 3.9 Hz, 1H), 3.69 (dd, *J* = 12.6, 6.8 Hz, 1H), 2.98 (d, *J* = 7.5 Hz, 2H), 2.50 (hept, *J* = 8.0 Hz, 1H), 2.28 – 2.21 (m, 2H), 2.02 (dd, *J* = 12.2, 8.8 Hz, 2H).

^{13}C NMR (151 MHz, CD_3CN) δ 155.01, 152.43, 141.72, 138.94, 136.91, 132.36, 127.94, 124.07, 123.53, 121.76, 121.76, 119.74, 116.61, 112.46, 71.90, 59.61, 39.55, 36.92, 35.33, 33.87.

HPLC Chromatograms

Recorded via Agilent 1260 series HPLC system, as described above.

Compound **1** (254 nm)

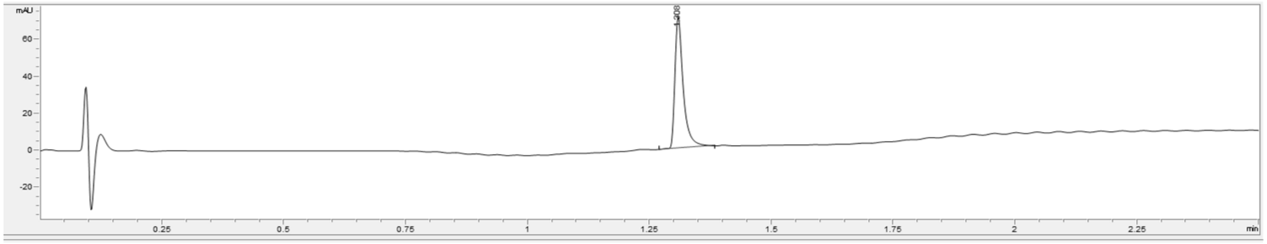

Compound **1a** (254 nm)

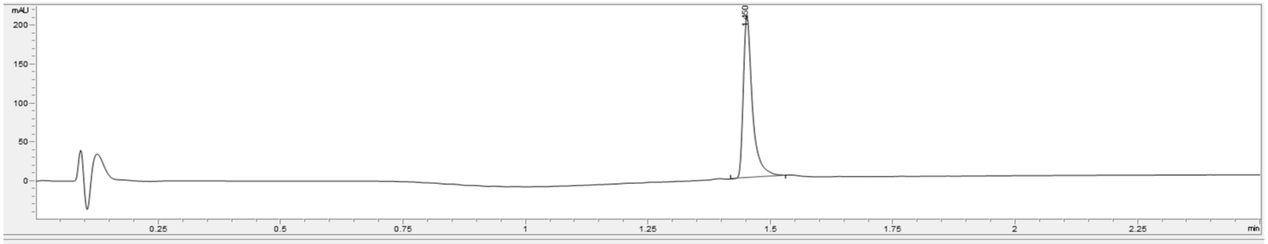

Compound **2** (254 nm)

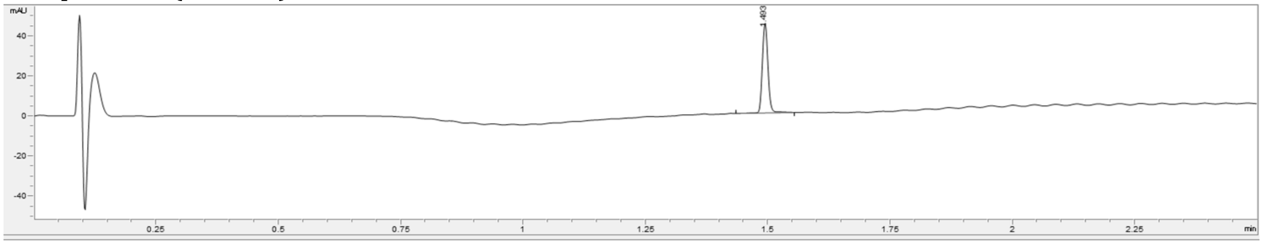

Compound **2a** (254 nm)

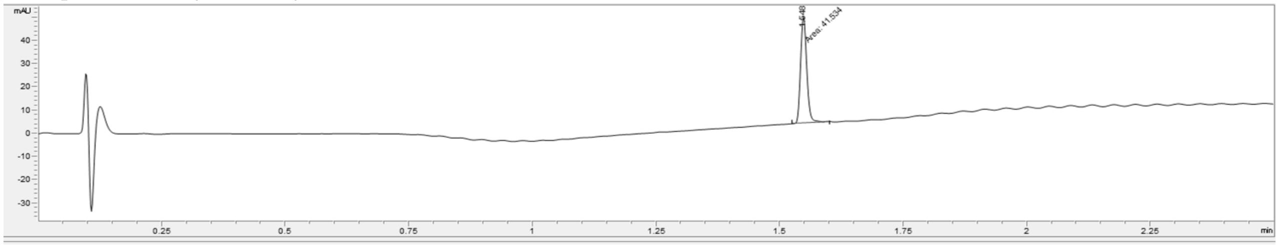

Compound **3** (254 nm)

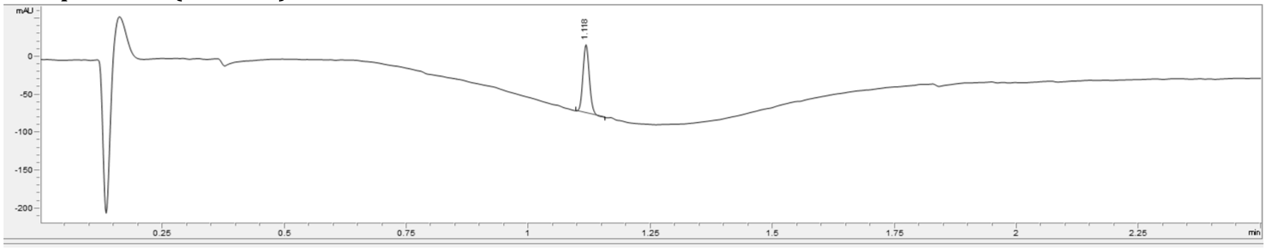

Compound **4** (254 nm)

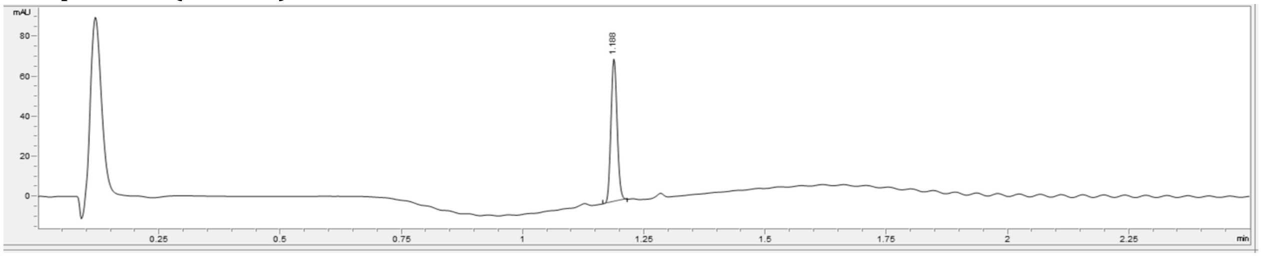

Compound **4a** (254 nm)

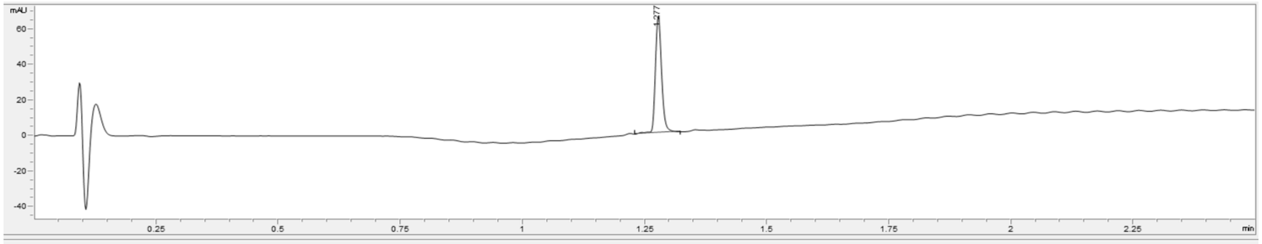

Compound **5** (500 nm)

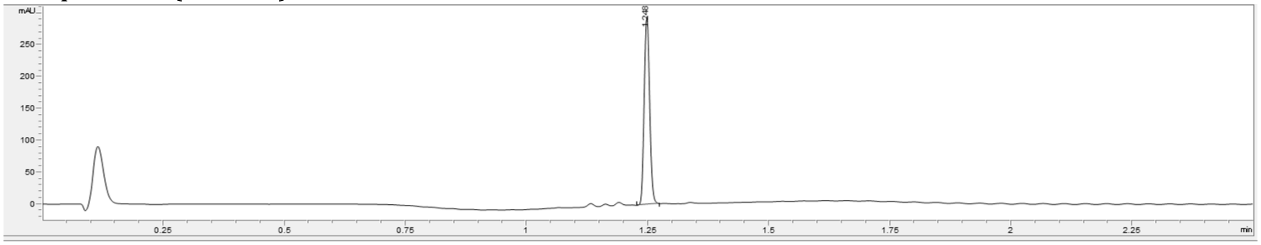

Compound **5a** (600 nm)

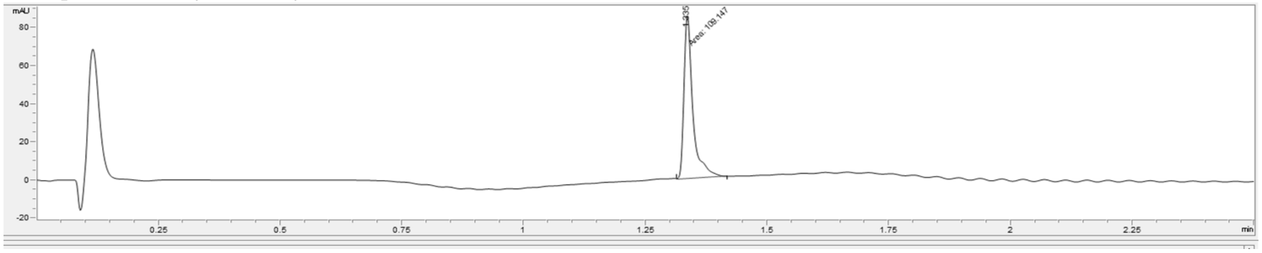

Experimental Details

In vitro MAGL Inhibition Assay

Compounds were profiled for MAGL inhibitory activity by determining the enzymatic activity by following the hydrolysis of the natural substrate 2-arachidonoylglycerol (2-AG), resulting in arachidonic acid, which can be followed by mass spectrometry. The 2-AG assay was carried out in 384 well assay plates (PP, Greiner Cat# 784201) in a total volume of 20 µL. Compound dilutions were made in 100% DMSO (VWR Chemicals 23500.297) in a polypropylene plate in 3-fold dilution steps to give a final concentration range in the assay from 12.5 µM to 0.8 pM. 0.25µL compound dilutions (100% DMSO) were added to 9 µL MAGL in assay buffer (50 mM TRIS (GIBCO, 15567-027), 1 mM EDTA (Fluka, 03690-100 mL), 0.01% (v/v) Tween. After shaking, the plate was incubated for 15 min at RT. To start the reaction, 10 µL 2-arachidonoylglycerol in assay buffer was added. The final concentrations in the assay were 50 pM MAGL and 8 µM 2-arachidonoylglyerol. After shaking and 30 min incubation at RT, the reaction was quenched by the addition of 40µL of ACN containing 4µM of d8-arachidonic acid. The amount of arachidonic acid was traced by an online SPE system (Agilent Rapidfire) coupled to a triple quadrupole mass spectrometer (Agilent 6460). A C18 SPE cartridge (G9205A) was used in an ACN/water liquid setup. The mass spectrometer was operated in negative electrospray mode following the mass transitions 303.1 → 259.1 for arachidonic acid and 311.1 → 267.0 for d8-arachidonic acid. The activity of the compounds was calculated based on the ratio of intensities [arachidonic acid / d8-arachidonic acid]. The lowest IC_{50} value that can be determined with high accuracy is approximately 0.025 nM.

NanoBRET Assay

This assay was performed as first described before.[20]

Compounds were profiled by a novel cellular MAGL NanoBRET™ Target Engagement assay, which measures the apparent affinity of test compounds by competitive displacement of a Roche-developed Tracer (WO2021058443, Example 21[21,] or probe **4a**, respectively), reversibly bound to a NanoLuc® luciferase MAGL fusion protein in cells.

A HEK293A stable cell line expressing NanoLuc_MAGL was established at Roche and assigned as HEK293A_NanoLuc_MAGL clone #43. A fixed concentration of tracer is added to cells expressing the NanoLuc®_MAGL fusion protein to generate a BRET reporter complex. The introduction of competing compounds results in a dose-dependent decrease in NanoBRET™ energy transfer, which allows quantification of the intracellular affinity of the target protein for the test compound.

HEK293A_NanoLuc_MAGL clone #43 cells were cultured in DMEM (Gibco 31966) with 10% FBS and 800 ug/ml G418. The NanoBRET assay was performed by seeding 5000 cells/well in Costar low-volume white 384-well plate in assay buffer (1% FBS in HBSS), followed by adding the tracer (50 nM Example 21 or 100 nM **4a** final concentration) and serial dilutions of test compounds. After 2 hours of incubation, Nanoluciferase substrates and extracellular inhibitors were added following the manufacturer's protocol (Promega, #N2520), and the NanoBRET signaling were quantified with the Paradigm (Molecular Devices).

Solubility measurement

High throughput kinetic solubility measurement was performed as previously described in the literature.[22]

Surface plasmon resonance

Immobilization
EDC/NHS coupling with a final protein concentration of 0.06 mg/mL: 10 mM acetate pH 5.5 + 10µM RO7224999-000-001(reference binder). No quenching was applied after immobilization, but the surface was left with a running buffer for 1 h to hydrolyze and stabilize.

Measurement Protocol
Running buffer: 0 mM Hepes, 150 mM NaCl, 0.05% P20, pH 7.4 + 50 µM EDTA, 1% DMSO Instrument: Biacore T200 Sensorchip: CM5 Temperature: 18 °C concentration series: 5 concentration 3-fold dilution, top conc 0.5 µM, contact time 180s, dissociation time 300s before each compound a blank injection for double referencing was measured. Each compound was followed by 9 injections of Control, RO7283571 (60 s contact time, 200 s dissociation time, 10 µM) to validate surface activity and recovery to determine off rates.

Data Analysis
Dissociation was trimmed for compounds showing faster kinetics to suppress the over-fitting of baseline drift. Data was fitted with 1:1 model with an added drift parameter to improve fit quality and to compensate from insufficient drift correction with the double referencing. Off rates (k_{off}) were determined by recovery of reference binding. The signal was first blank subtracted, and the corrected chaser response was plotted versus time and fitted to an exponential decay model.

Crystal Structure

Crystallization, data collection and structure determination of the human MAGL in complex with compound 5
Human MAGL protein with mutations introduced at positions Lys36Ala, Leu169Ser and Leu176Ser[23] was produced by Cepter Biopartners LLC (Nutley, USA). A DNA fragment spanning the human His6-TEV-MAGL (K36A, L169S, L176S) was synthesized (GenScript) and inserted into pET28-using NcoI/EcoRI restriction sites, then transformed into *Escherichia coli* BL21(DE3) (Novagen). Cultures were inoculated and maintained in logarithmic growth at 37°C in LB medium containing appropriate antibiotics. When the A_{600} of cultures reached 0.6–0.8, the cultures were chilled on ice, then expression was induced with 1 mM IPTG for 16 h at 23°C. Cells were harvested by centrifugation and stored at −80°C. The cell pellet was suspended in Buffer A [50 mM Tris-HCl (pH 7.5), 500 mM NaCl, 10% glycerol] with Benzonase (Sigma, 1:20000 dil.). The cells were disrupted by a microfluidizer, and insoluble material was removed by centrifugation at 40,000 rpm for 45 min (Beckman, 45Ti). The soluble lysate was filtered through a Steritop™ 0.22 µm PES membrane (Millipore), then loaded

onto a HisTrap FF (GE Healthcare, 5 ml equilibrated in Buffer A. After washing with 25 mM imidazole in Buffer A, His6-TEV-MAGL(K36A, L169S, L176S) was eluted with 25 – 500 mM imidazole in Buffer A. Peak fractions containing His6-TEV-MAGL(K36A, L169S, L176S) were pooled and incubated with His6-TEV protease for overnight with dialysis against 2 L of Buffer B [50 mM Tris-HCl (pH 7.5), 200 mM NaCl, 5% glycerol, 2 mM DTT]. In order to remove cleaved His6-tag and His6-TEV protease, the mixture was re-loaded onto HisTrap FF, and the flowthrough fraction containing tag cleaved MAGL was collected. The target protein was concentrated to an appropriate volume using Amicon Ultra-15 (Millipore, 10 kDa MWCO) and loaded onto a HiLoad Superdex 75 26/60 (GE Healthcare), which was equilibrated in Buffer C [50 mM HEPES (pH 7.5), 200 mM NaCl, 2 mM DTT, 2 mM EDTA, 2% glycerol]. MAGL containing fractions were pooled. A typical yield of tag-free hMAGL (K36A, L169S, L176S) was around 2.5-3.0 mg per 1 L LB culture.
For crystallization the protein was concentrated to 10.8 mg/mL. Crystallization trials were performed in sitting drop vapor diffusion setups at 21 °C. Crystals appeared within 2 days out of 0.1M MES pH 6.5, 11% PEG MME5K, 12% isopropanol. The structure of hMAGL (K36A, L169S, L176S) with compound 5 was obtained by soaking crystals for 16 h in crystallization solution supplemented with 10 mM inhibitor dissolved in DMSO.

For data collection crystals were flash cooled at 100 K with 20 % ethylene glycol added as cryo-protectant. X-ray diffraction data were collected at a wavelength of 1.0000 Å using an Eiger2X 16M detector at the beamline X10SA of the Swiss Light Source (Villigen, Switzerland). Data have been processed with XDS[24] and scaled with SADABS (BRUKER). The crystals belong to space group $C222_1$ with cell axes of a= 90.45 Å, b= 127.31 Å, c= 62.84 Å and diffract to a resolution of 1.43Å. The structure was determined by molecular replacement with PHASER[25] using the coordinates of PDB entry 3PE6 as search model. Difference electron density was used to place compound 5. The structure was refined with programs from the CCP4 suite[26] and BUSTER. Manual rebuilding was done with COOT[27]. The coordinates of the structure were deposited in the PDB under the accession code 8RVF. Data collection and refinement statistics are summarized in Table S3.

Table S3. Data collection and refinement statistics. Values in parentheses are for highest-resolution shell.

	hMAGL compound 5
**Data collection**	
**Space group**	**$C222_1$**
**Cell dimensions**	
**a, b, c (Å)**	**90.45, 127.31, 62.84**
**α, β, γ (°)**	**90, 90, 90**
**Resolution (Å)**	**1.43 (1.53-1.43)**
**Total reflections**	**502186**
**Total unique reflections**	**67210**
**R_{sym}**	**0.078 (1.00)**
**I / σI**	**8.34 (0.58)**
**CC(1/2)**	**0.999 (0.661)**
**Completeness**	**100.0 (100.0)**
**Redundancy**	**7.47 (7.32)**
**Refinement**	
**Resolution (Å)**	**63.66 – 1.43**
**No. reflections**	**67100**
**Rwork / Rfree**	**19.48/22.20**
**No. atoms**	
**Protein**	**2320**
**Water**	**310**
**Ligand**	**39**
**B-factors**	
**Protein**	**35.15**
**Water**	**52.25**
**Ligand**	**34.17**
**R.m.s. deviations**	
**Bond lengths (Å)**	**0.011**
**Bond angles (°)**	**1.070**
**PDB code**	**8RVF**

Fluorescence Polarization Assay

Determination of the K_d of the fluorescent probes

Commercially available purified hMAGL protein (Cepter Biopartners, Nutley, NJ, USA) was diluted in PBS-buffer pH 7.4 + 0.1% Tween 80, in a range of concentrations and incubated with compound **5** (20 nM = $[L]_T$) at ambient temperature for 60 min. Fluorescence polarization was determined via a Tecan Spark multimode plate reader (Ex/Em = 482/502 nm, 7.5 nm bandwidth each) in a 384 flat black well plate (Corning); six measurements per data point. The experiment was carried out in triplicate. Data were analyzed via GraphPad Prism 5.0 software. K_d of the fluoroprobe was calculated from the observed MAGL concentration at half-maximal FP (FP_{50}) as follows.

$$K_d = \frac{[L][P]}{[LP]} = \frac{0.5\,[L]_T\,(FP_{50} - 0.5\,[L]_T)}{0.5\,[L]_T} = FP_{50} - 0.5\,[L]_T$$

Competitive FP Binding Assay

hMAGL in PBS-buffer pH 7.4 + 0.1% tween 80 at a final concentration of 50 nM was incubated with a range of inhibitor DMSO solutions (5µL) (serial dilution 1:3) for 30 min at ambient temperature in a 384 flat black well plate (Corning). Fluoroprobe **5** in buffer was added at a final concentration of 20 nM. After 30 min incubation at ambient temperature, fluorescence polarization was determined as described above. Data were analyzed via GraphPad Prism 5.0 software, and IC_{50} values were converted to K_i according to Nikolovska-Coleska et al. [28] Under these conditions the assay performed with an average z´= 0.90.

$$z' = \frac{3(\sigma_{pos} + \sigma_{blank})}{\mu_{pos} - \mu_{blank}}$$

ABPP and in gel staining

Mouse brain sample preparation

Mouse tissues were isolated according to guidelines approved by the ethical committee of Leiden University. Male mouse brain (27 weeks old) was homogenized with glass beads (2x 1 min, bullet blender, speed 8) using cold lysis buffer (20 mM Hepes pH 7.2, 1 mM $MgCl_2$, 2 U/mL Benzonase). The membrane and cytosol were separated by centrifugation. The membrane fraction was resuspended in Hepes/DTT buffer. Protein concentration was determined with Bradford Assay, and samples were diluted to a final concentration of 2.0 mg/mL. Samples were snap-frozen in liquid nitrogen and stored at -80 °C until further use.

Competitive ABPP

Lysate (19 µL, 2 µg/µL) was thawed on ice. For comparative ABPP (irreversible probes), 1 µL of probe (20x stock in DMSO) or pure DMSO was added to the sample, vortexed briefly, and incubated for 30 minutes at RT. For competitive ABPP (reversible probes), 0.5 µL of the reversible probe (40x stock in DMSO) or pure DMSO (as vehicle) was added to the sample, vortexed briefly, and incubated for 30

minutes at RT. Subsequently, 0.5 µL MB064 (40x stock in DMSO) was added to the proteome sample, vortexed briefly, and incubated for 10 minutes at RT, after which 0.5 µL FP-Bodipy-FL (40x stock in DMSO) was added, vortexed briefly and incubated for 10 minutes at RT. The final volume was 20 - 20.5 µL (5 - 7.5% DMSO). The reaction was quenched by the addition of 7.5 µL of 4*Laemmli-buffer (final concentrations: 60 mM Tris (pH 6.8), 2% (w/v) SDS, 10% (v/v) glycerol, 1.25% (v/v) β-mercaptoethanol, 0.01% (v/v) bromophenol blue). 10 µL (14 µg protein) of the quenched reaction mixture was resolved on 10% acrylamide SDS-PAGE (180 V, 75 min). Fluorescence was measured using a Biorad ChemiDoc MP system (fluorescence channels Cy2, Cy3, Cy5). Gels were then stained using coomassie staining and imaged for protein loading control. Labeling of the respective proteins via MB064 and FP-BODIPY was visualized in two separate channels.

Final concentrations

- MBP = 14 µg
- MB064 = 250 nM
- FP-Bodipy = 100 nM

PBMC preparations

Peripheral blood mononuclear cells (PBMCs) were prepared from buffy coats obtained from the Blutspendedienst Zentralschweiz SRK after written informed consent from the donors according to the Swiss Guidelines (Blutspende SRK Schweiz Generalkonsent 2019/3_ Version 1.0). Briefly, PBMCs were isolated by Ficoll-Paque (1.077 g/mL density) (17–1440–02; GE Healthcare Bio-sciences) density gradient centrifugation at 400 × g at 18°C for 35 min. Cells were washed several times using 1× PBS and frozen in CryoStor® (C2874; Sigma) until further use.

Activity based protein profiling (ABPP)

PBMCs were thawed, washed with 1 x PBS and equal amounts of cells were exposed to DMSO (D2650; Sigma-Aldrich) or MAGL inhibitor **PF** (PF-06795071) in DMSO for 30 min at room temperature with concentrations ranging from 1 nM to 1000 nM. Cells were washed with 1 x PBS and lysed in ice-cold lysis buffer (ab156035; Abcam), lysates were centrifuged at 14,000 x g for 15 minutes at 4°C and supernatants were collected. For ABPP experiments, cell lysates containing equal amounts of protein were incubated with the appropriate concentration of activity-based probe for 30 min at room temperature. The reaction was quenched for 30 min by adding SDS-PAGE loading buffer (NP0007, Thermo). Samples were then separated by SDS-PAGE and bands were detected ChemiDoc Imager (Bio-Rad Laboratories). Following detection, gels were stained with SimplyBlue™ SafeStain (LC6060, Thermo) for 1 hour with gentle agitation and excess stain was removed by destaining in ddH2O for 1 h with gentle agitation. Gels were imaged and protein bands were quantified using Image Lab software (Bio-Rad Laboratories).

MAGL Protein Mass Spectrometry

Intact MAGL protein was incubated with the indicated probe concentrations at 37 °C in PBS pH 7.4 for 30 min. 30 µL test samples were combined with 10 µL MeCN and put on ice until further analysis. The protein was analyzed using a Waters H-class instrument equipped with a quaternary solvent manager, a Waters sample manager-FTN, a Waters PDA detector and a Waters column manager with an Acquity UPLC protein BEH C4 column (300 Å, 1.7 µm, 2.1 mm x 50 mm). Proteins were eluted with a flow rate of 0.3 mL/min with 80°C column temperature. The following gradient was used: A: 0.01% FA in H_2O; B: 0.01% formic acid in MeCN. 5-95% B 0-6 min. Mass analysis was conducted with a Waters XEVO G2-XS QTof analyzer. Raw data was deconvoluted with MaxEnt 1.

Flow Cytometry and Cellular Imaging with HT-29 and A549 cells

Flow cytometry. HT-29 cells were cultured in DMEM medium (Gibco) supplemented with 10% heat inactivated FBS in a humidified atmosphere at 37°C and 5% CO_2 HT-29 cells ($0.8x10^7$) were incubated with or without inhibitor (PF-06795071, 100µM) using medium without serum and Phenol red (500µl) for 1 hour, washed once, re-suspended with medium and distributed to sample aliquots of 50 µl each. Pre-diluted fluorescent probes were added for indicated final concentrations, incubated for 10 min, 20 min, and 30 min at 37°C and washed once. Live cell measurements were carried out with the MACSQuant® X Flow Cytometer (Miltenyi Biotec, laser excitation 488, emission detector 525/50 nm). Each condition was tested in triplicate, all data were confirmed in two independent experiments.

Fluorescence confocal microscopy. HT-29 and A549 cells were cultured in DMEM medium (Gibco) supplemented with 10% heat inactivated FBS in a humidified atmosphere at 37°C and 5% CO_2. The imaging experiments were conducted in a 384-well microplate format (PhenoPlate, Revvity). Fluorescent probes were stored as 10mM stocks in DMSO and pre-diluted before applying to cells. Experiments were performed in at least two independent experiments. Cells were dispensed at a density of 2500 cells/well (40µl) onto a microplate. After incubation for 48 h, the medium was replaced to serum free conditions without Phenol red (20µl). Fluorescent probes were added (10µl) during imaging process after image capturing of starting conditions and tested at different concentrations (150nM, 250nM, 500nM). In case of blocking experiments, cells were incubated with inhibitor (PF-06795071, 10 µM) for 90 min before probe administration.

Localization experiments. Cells were plated onto a microplate at a density of 2500 cells/well (40 µl) and incubated for 48 h. After replacement of medium to serum free conditions without Phenol red (20 µl), different cell compartments were stained using ER-Tracker™ Red (Invitrogen) or Mito-Tracker™ Deep Red (Invitrogen), for 30 min, followed by a washing step with medium. Cells were incubated for 30 min with fluorescent probes (10 µl, 150nM) and washed three times with medium (50µl) before live cell confocal imaging. Samples without fluorescent probe but with ER-tracker, fluorescent probe plus or without ER-tracker served as controls.

Probe uptake into cells experiment. HT-29 cells were seeded at a density of 2500 cells/well and incubated for 72 h. After nuclei stain with Hoechst 33342 (0.9µM), the medium was changed to 1%

FBS without Phenol red. The cell membrane was stained using CellMask™ plasma membrane stain (CellMask™-A647, Invitrogen) for 30 min, before starting the imaging time series at 63x magnification (stack of 55 planes, distance 0.5µm, 1 image/well) and adding the probe.

Confocal live cell image acquisition. Confocal live cell imaging was performed using the Opera Phenix™ High Content Screening System (Revvity). The probe fluorescence was monitored by kinetic measurements of 10 min with a break for probe administration. The fluorescence of one image per sample was captured at each time point. Probe detection was realized using the appropriate laser for excitation and filter for fluorescence emission. Image acquisition parameters, including laser power, offset, and gain settings, were kept constant. All cell imaging measurements were done using water immersion objectives (63xWater-NA 1.15, 40xWater-NA 1.1).

Confocal Imaging of Hippocampal Neurons

Preparation of primary neuron cultures. For neuron culture, glass coverslips (19 mm, #1.5 Epredia Deckgläser, CB00190RAC20MNZ0) were coated with PLL (Sigma-Aldrich, P4707; 1:12 dilution) in phosphate-buffered saline (PBS) for 2h at 37 °C. Hippocampal neurons were then prepared from P0 wild-type mice as previously described[29] and plated at a density of 200k cells per coverslip in neuronal medium (penicillin [200 U/mL]/streptomycin [200 µg/mL[[Gibco, 15140-130], 2 mM GlutaMAX [Gibco, 35050-038], 2 % B27 [Gibco life technologies 17504-044] in Neurobasal-A), and grown for 2-3 weeks in an incubator at 37 °C and 5% CO_2. The medium was replaced once the day after plating.

Fluorescent labeling and image acquisition. Fluorescent dyes were added to the cell culture media (final concentrations as indicated) and incubated for 15 min at 37 °C and 5% CO_2. As a negative control, coverslips were treated with 10 µM **PF** (PF-06795071) for 2h at 37 °C and 5% CO_2 prior to fluorescent labeling. Neurons were subsequently washed in PBS twice, mounted on a grooved microscope slides in PBS, and imaged live on a Zeiss LSM 710 confocal laser scanning microscope for a maximum of 30 min. Images were then analyzed using FIJI.

Confocal Imaging of Human Brain Organoids

Generation of human brain organoids. Human brain organoids were generated from neural progenitor cells differentiated from the human induced pluripotent stem cell (hiPSC) line BIHi250-A (https://hpscreg.eu/cell-line/BIHi250-A) as previously described[30] with minor modifications: from day 45 on, organoids were cultured in neural differentiation medium (NDM) lacking rhNT3 and rhBDNF until they were harvested on day 83.

Fluorescent labeling and image acquisition. On day 83 in culture, brain organoids were incubated in neural differentiation medium containing 5 µM probe **1** for 2 hours in a humidified incubator at 37°C and 5 % CO_2 on an orbital shaker at 60 rpm. Control organoids were pre-incubated with 10 µm **PF** for 2 hours, before probe **1** was added.

Immediately after labeling, organoids were washed twice with DPBS (Gibco 14200-075 diluted in dH_2O to 1x concentration) for 5 min. and fixed in 4 % PFA at 4°C overnight. Subsequently, fixed organoids were washed twice with DPBS for 5 min., incubated in 10 % sucrose for 24 hours and then in 30 % sucrose for 24 hours at 4°C as cryoprotectant (until the organoids no longer floated in the solution), embedded in TissueTek O.C.T. (Sakura 4583) and frozen at -80°C. Twenty µm thick tissue sections were cut on a cryostat (ThermoScientific CryoStar NX50) and stored at -80°C until used for imaging or further staining.

For immunostaining, tissue sections were washed with PBS (Gibco 70011-036 diluted in dH_2O to 1x concentration) for 5 min., blocked with 5 % normal donkey serum (Sigma-Aldrich S30), 0.3 % Triton X-100 (Sigma T8787), 0.1 % BSA (Sigma A7906) in PBS for 1 h, and stained with a mouse-anti-MAP2 primary antibody (Sigma M9942, diluted 1:200 in blocking buffer) over night at 4°C. After washing three times with PBS containing 0.3 % Triton X-100 (PBS-T), sections were stained with a donkey-anti-mouse IgG-secondary antibody coupled to AlexaFluor 647 (Invitrogen A31571, diluted 1:500 in blocking buffer) for 1 h at room temperature. Sections were washed three times with PBS-T for 5 min., nuclei stained with Hoechst 33342 (Invitrogen H1399) at 10 µM in PBS for 15 min, and washed with PBS once for 5 minutes. Stained sections were embedded in ProLong Glass Antifade Mountant (Invitrogen P36980).

Confocal spinning disc microscopy images were obtained on a PerkinElmer Opera Phenix high-content screening system controlled by Harmony 4.9 software using a 40x water objective (NA = 1.1). Excitation (ex.) laser / band pass emission (em.) filter sets for: Hoechst (375 nm ex., 435 – 480 nm em.), BODIPY (488 nm ex., 500 – 550 nm em.), AlexaFluor 647 (640nm ex., 650 to 760 nm em.). All slides were imaged with identical laser power and exposure settings. Images were exported from Harmony as TIF and stored and processed for figure display on OMERO Plus (Glencoe Software). Figures were prepared using OMERO.figure, channels were scaled to equal intensities across figure panels.

^{18}-F labeling of Compound 2 and ex vivo tissue staining

Radiochemistry

Compound **2** was dissolved in anhydrous MeCN and prepared as a stock solution with a concentration of 3 mg/mL. 6 μL stock solution was mixed with 7.1 μmol $SnCl_4$ in the reaction vial on the experimental day. Fluorine-18 was produced by the $^{18}O(p,n)^{18}F$ reaction using a Cyclone 18/9 cyclotron (18 MeV; IBA, Belgium), and the aqueous solution containing [^{18}F]fluoride ion was trapped on a QMA cartridge (Waters SepPak Accell QMA cartridge carbonate). Milli-Q water (~ 5 mL) was first passed through the cartridge for washing, and 75 mM tetraethylammonium bicarbonate in MeCN/ H_2O (v/v = 4/5) was applied to elute the radioactivity. After azeotropic drying with MeCN (1.0 mL × 2), the residue was dissolved in 1 mL anhydrous MeCN. An aliquot of [^{18}F]fluoride (~100 μL) was distributed to the reaction vial for radiolabeling. After shaking at room temperature for 10 min, the reaction was quenched with 10 mL 0.1% TFA in MilliQ water. The resulting mixture was passed through a preconditioned MCX cartridge and washed with 3 mL MilliQ water. Afterward, the product was eluted with 1 mL EtOH.

Quality control and *in vitro* stability

The purity of the product was analyzed by Agilent 1100 series HPLC system equipped with a UV detector and a GabiStar radiodetector (Raytest) using an ACE XDB-C18 Zobrax column (75 mm × 4.6 mm, 3.5 μm). The mobile phase A and B were 0.1% H_3PO_4 in Milli-Q water (v/v) and acetonitrile, respectively. Gradient method with 0.0–6.0 min, 5–60% B; 6.0–9.0 min, 60–95% B; 9.0–10.0 min, 95% B; 10.0–11.0 min, 95–5% B with a wavelength of 489 nm at the flow of 1 mL/min was used. The identity of the radioactivity was confirmed by co-injection with the reference compound.

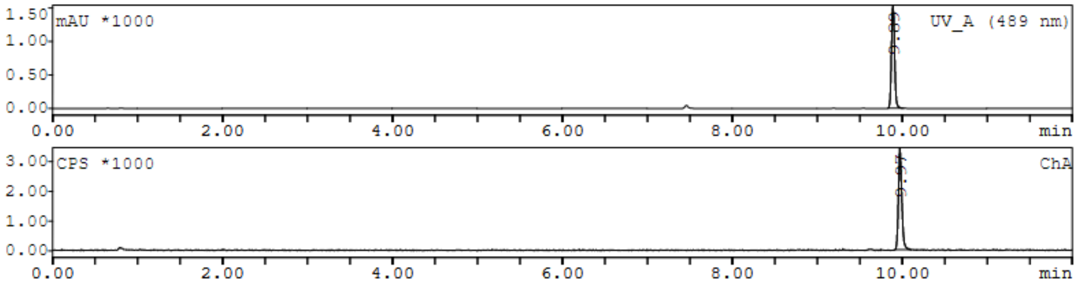

Figure S3: *HPLC co-injection of* ***[^{18}F]2*** *and* ***2***

For *in vitro* stability analysis, the product in the final formulation was stored at the room temperature and injected into Waters Acquity ultra-performance liquid chromatography (UPLC) equipped with BEH C18 column (ACQUITY UPLC BEH C18 Column, 130Å, 1.7 μm, 2.1 mm X 100 mm) and FlowStar LB 513 radioactivity flow detector. A gradient method using 0.1% H_3PO_4 in Milli-Q water as mobile phase A and MeCN as mobile phase B was employed at the flow of 0.6 ml/min for analysis (0.0-4.0 min, 5–95% B; 4.0–6.0 min, 95% B).

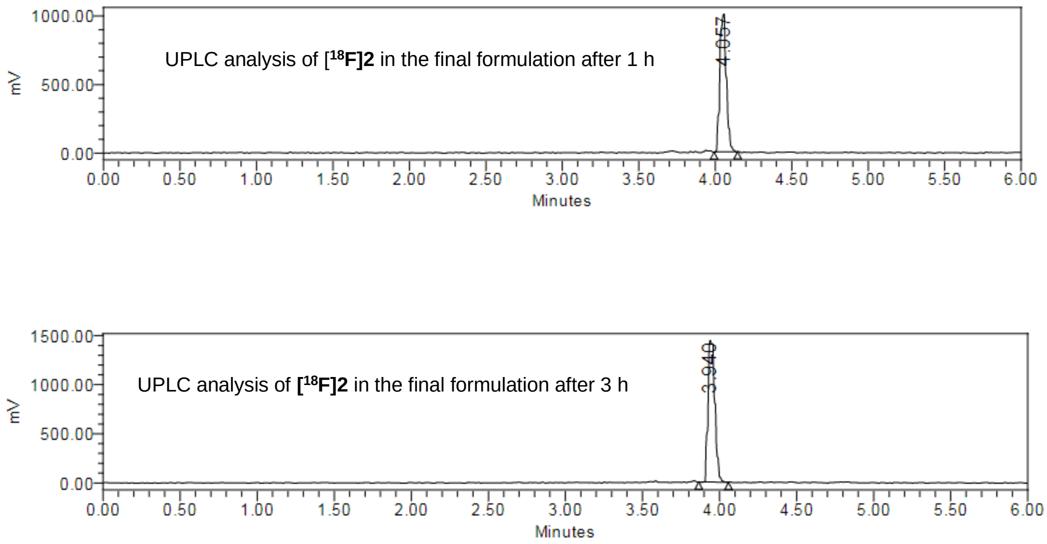

***Figure S4**: In vitro stability of **[^{18}F]2** in the formulation.*

In vitro autoradiography

Frozen brain tissues of MAGL KO and corresponding WT mice were cut with a thickness of 10 µm on a cryostat (Cryo-Star HM 560 MV; Microm, Thermo Scientific, Wilmington, DE) and stored at −20 °C before use. The *in vitro* autoradiography was carried out as reported previously.[31] Briefly, the slices were thawed on ice and subsequently immersed into aqueous 30 mM HEPES buffer containing 3% fatty acid-free bovine serum albumin (BSA) at 0 °C for 10 min for precondition. Upon drying, incubation was carried out in a humidified chamber with ~45 nM **[^{18}F]2** at room temperature for 30 min. After incubation, the slices were decanted and washed in ice-cold 30 mM HEPES buffer containing 3% fatty acid-free BSA (1 × 5 min), 30 mM HEPES buffer (2 × 3 min), and water (2 × 5 s). The slices were then exposed on phosphor imager plates (Fuji, Dielsdorf, Switzerland) for 30 min, and the film was read by a BAS5000 reader (Fuji). The data analysis was performed using AIDA 4.50.010 software (Raytest Isotopenmessgeräte GmbH, Straubenhardt, Germany).

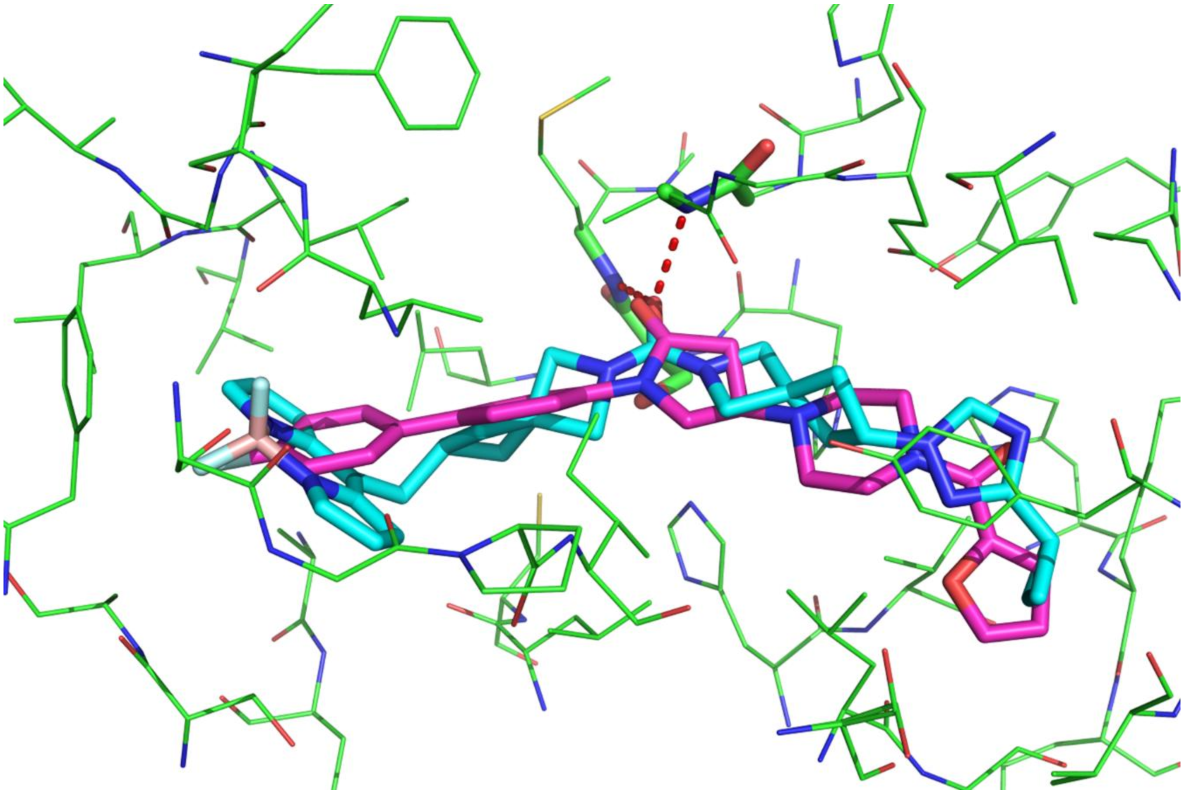

Figure S5. *X-ray co-crystal structure of compound **5** (cyan) in complex with hMAGL (PDB: 8RVF) and superimposition with compound **13** (magenta, PDB: 7PRM). Hydrogen bonds of the central C=O group with the oxyanion hole residues (Ala 51, Met 123) are shown as dashes.*

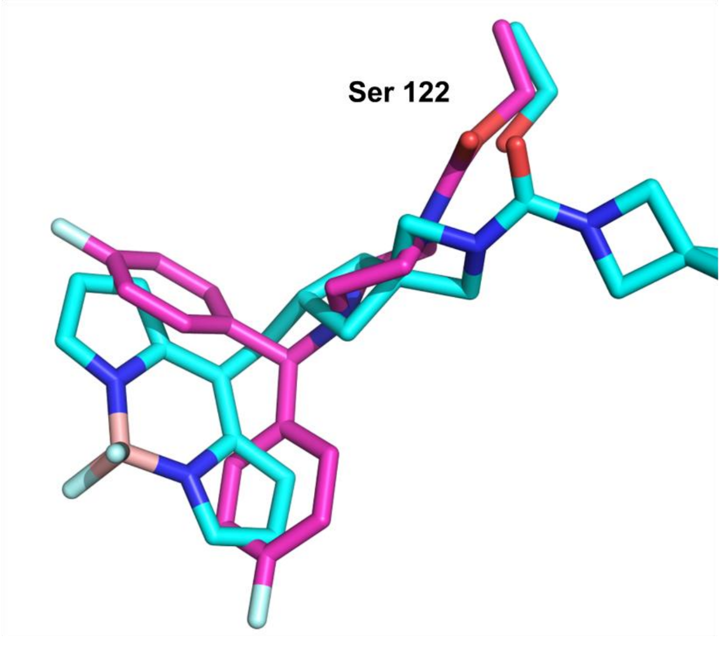

Figure S6. *Overlay of ligand binding modes in hMAGL and interaction with catalytic Ser122 of non-covalent compound **5** (cyan, PDB: 8RVF) and covalent SAR629 (magenta, PDB: 3JWE). Terminal BODIPY fragment from compound **5** can be viewed as a closed form of the phenylmethylbenzene group of SAR629.*

Microscopic Images

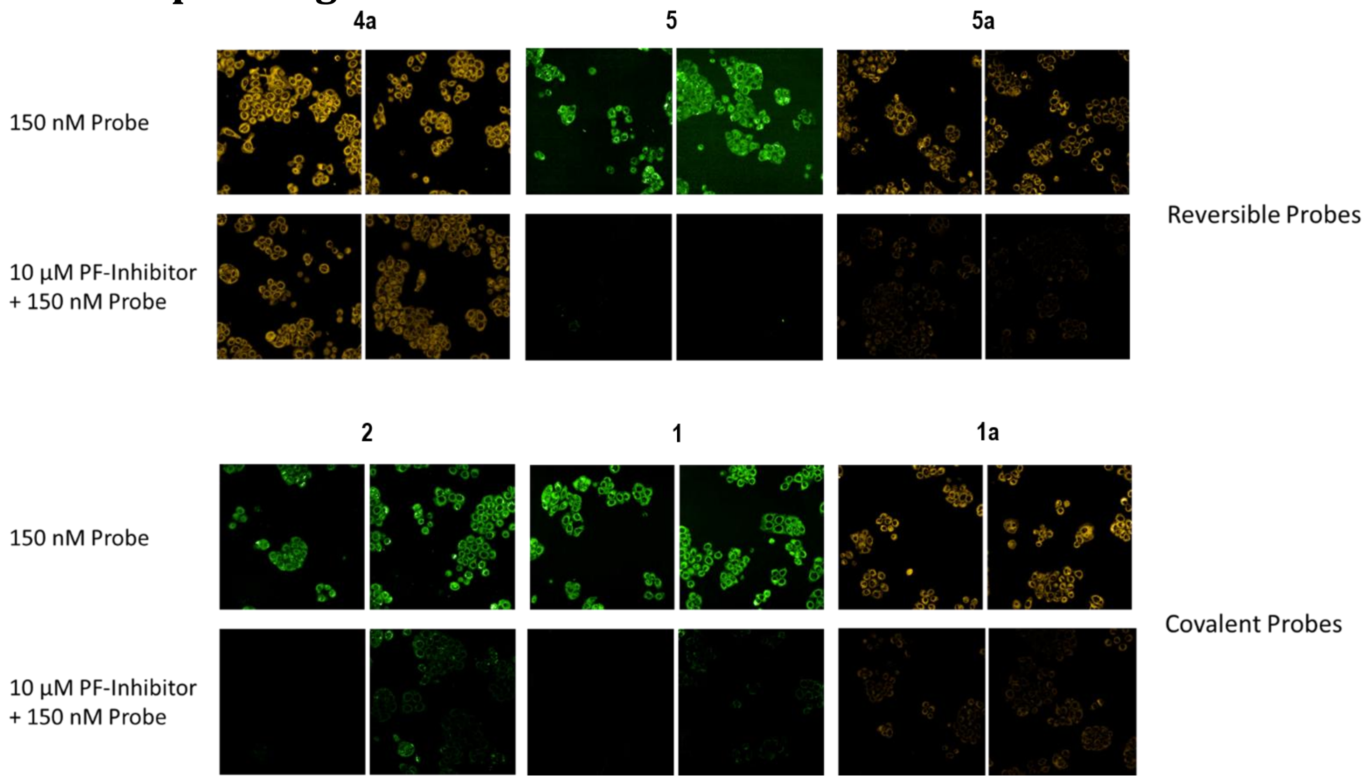

Figure S7. *Labeled HT-29 cells with the respective fluorescent probes at 150 nM concentration after 10 min incubation time without any washing steps. MAGL-specific signal blocking via pre-incubation with 10 µM **PF**-inhibitor. Representative duplicates are shown.*

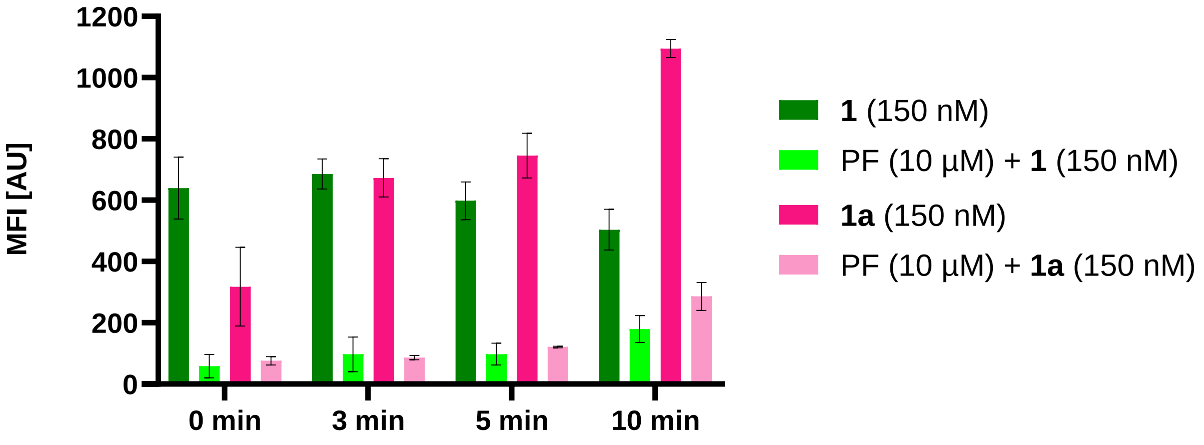

Figure S8. *Fluorescence intensity comparison between **1** and **1a**. Mean fluorescence intensity of HT-29 cells with the respective fluorescent probes at 150 nM concentration at various time points without any washing steps. The mean fluorescence intensity of n=2-3 ± SD is shown. Probe **1** fluorescence is monitored at Ex: 488 nm Em: 500-550 nm 75% laser power and **1a** at Ex: 561 nm Em: 570-630 nm 50% laser power at identical exposure times of 0.1 s.*

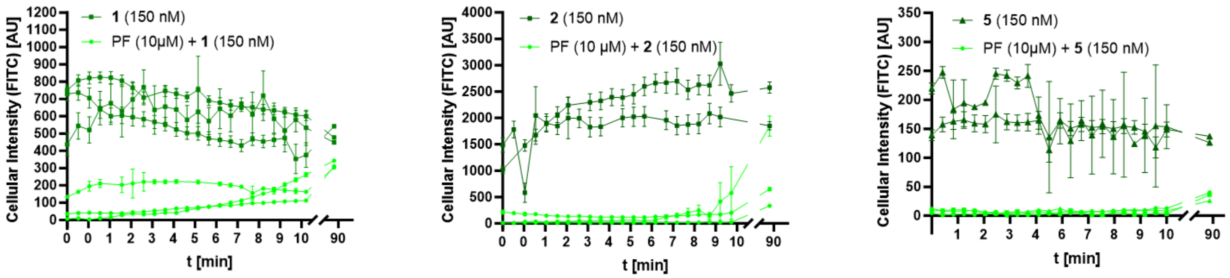

Figure S9. *Kinetic MAGL staining of HT-29 cells. Time-dependent mean fluorescence intensity ± 95% confidence interval, detected inside HT-29 cells (~100 cells per datapoint) of the key fluorescent probes **1**, **2** and **5**; with and without preincubation of PF (10 µM).*

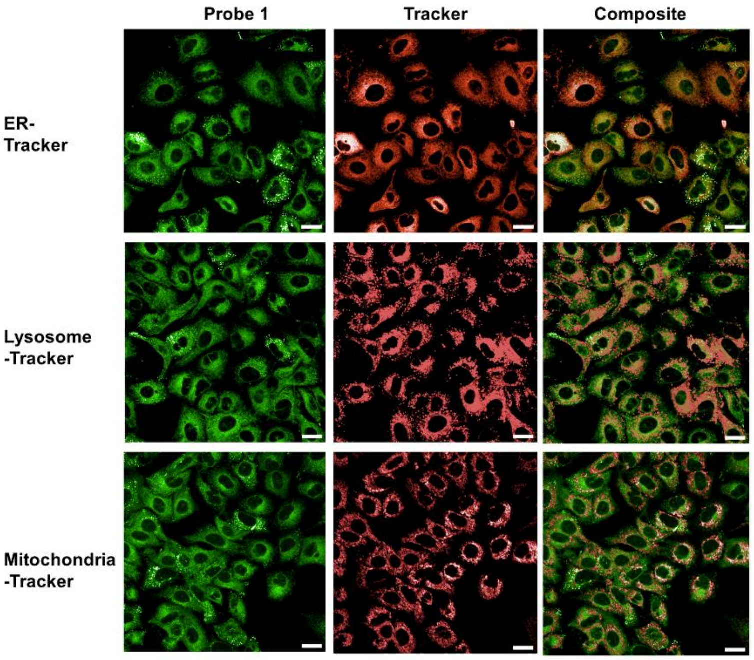

Figure S10. *Confocal microscopic images of A549 cells labeled with probe **1** (150 nM, FITC channel) and with the respective commercial compartmental trackers (Cy3 channel). Only ER-Tracker Red (Invitrogen) shows colocalization. Scalebar = 20 µm.*

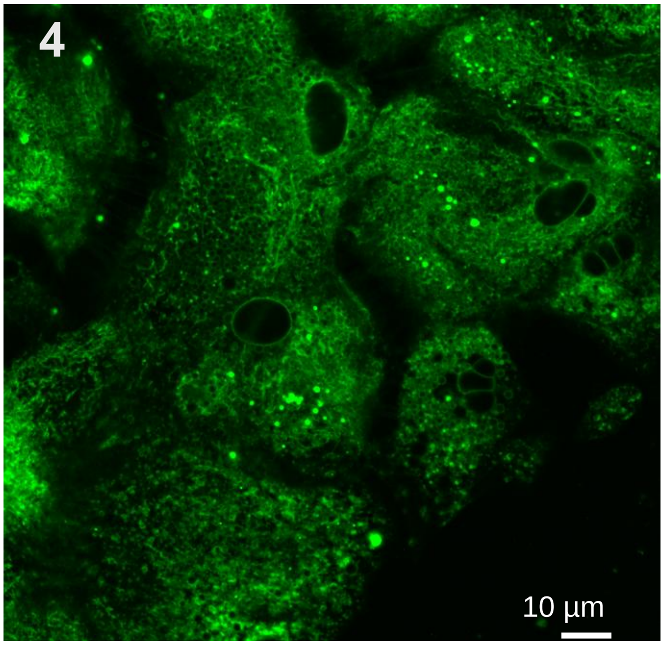

Figure S11. *Primary astrocytes in culture are intensely stained with probe* ***4*** *(1 µM).*

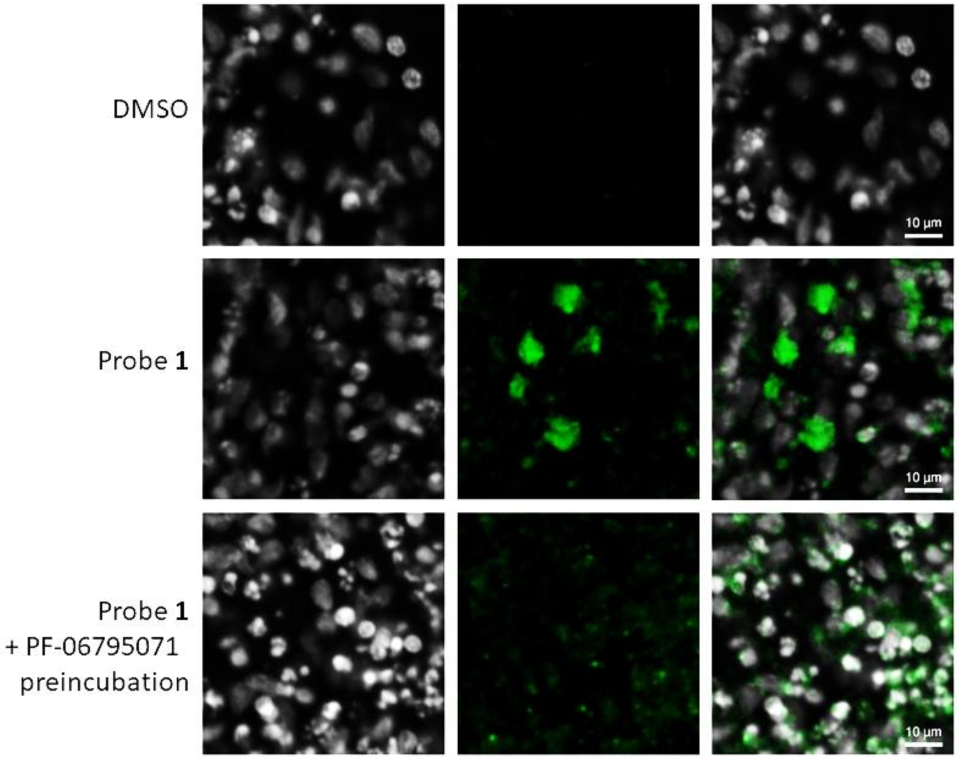

Figure S12. *Human hiPSC derived brain organoids, competition experiment with* ***PF*** *(PF-06795071) preincubation. Confocal micrographs of brain organoids incubated with 10 µM* ***PF*** *(PF-06795071) for 2 hours prior to incubation with 5 µM probe* ***1*** *for 2 hours after 83 days of organoid maturation. Organoids were fixed, cryosectioned and stained for nuclei (Hoechst) prior to confocal imaging. Scale bars: 10 µm.*

Characterization of the reversible / covalent behavior of the fluorescent probes 4, 5 and 1

SPR data of reversible probes **4** and **5**

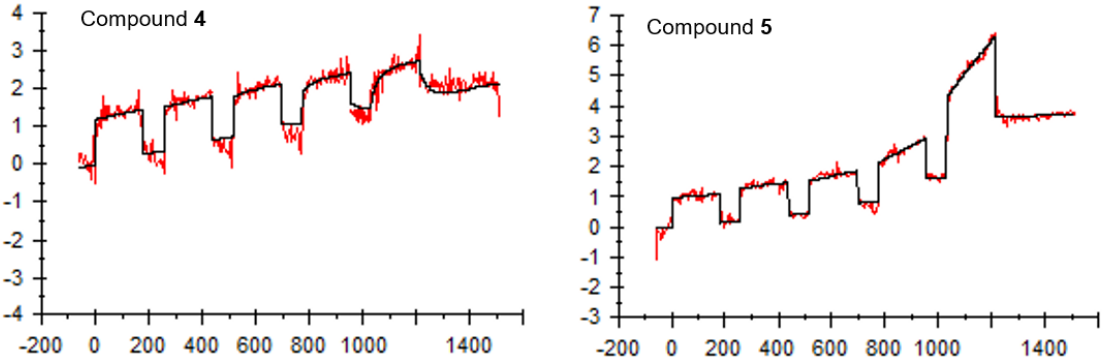

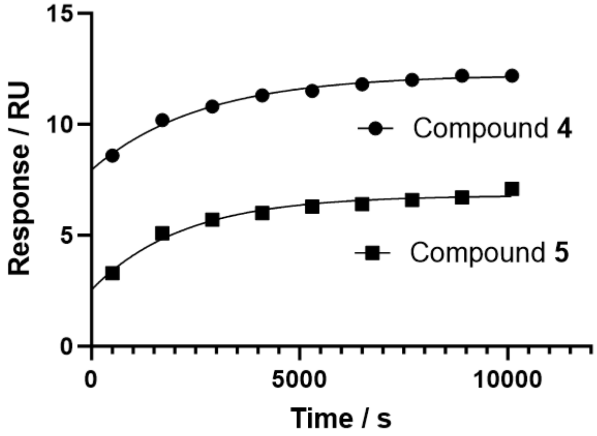

Compd.	K_{on} [s^{-1}]	K_{off} [M^{-1} s^{-1}]	Residence Time [min]	K_D [nM]
4	2.0E+04	4.5E-04	37	23
5	7.2E+03	3.8E-04	44	52

Figure S13. *Surface plasmon resonance experiment of compounds **4** and **5** on human MAGL. Depicted are two representative sensorgrams with the respective fitting models as described above and the recovery of reference binder to determine dissociation rates. Association constants (k_{on}), dissociation constants (k_{off}), plus the dependent residence time and binding constant K_D, are depicted.*

MS-based covalency investigation of 1 and 5

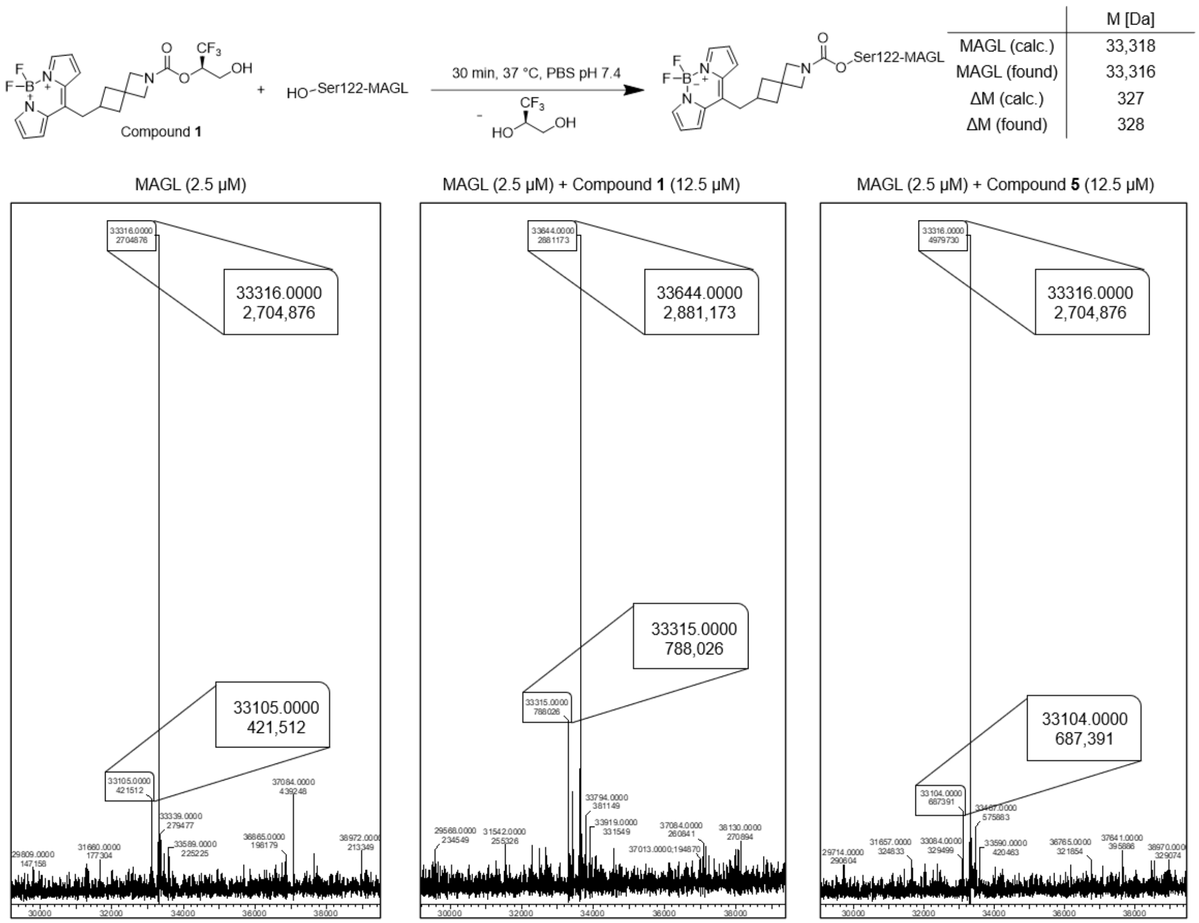

Figure S14. *Reaction of compound **1** with hMAGL (full length, consisting of 304 amino acids with an extra N-terminal glycine generated by TEV proteolytical cleavage), covalently binds Ser122, with TFMG as leaving group. QTOF mass spectra of unlabelled MAGL protein and MAGL incubated with either covalent probe **1** or reversible probe **5**. Detected mass and ion count highlighted.*

Photo-physical properties

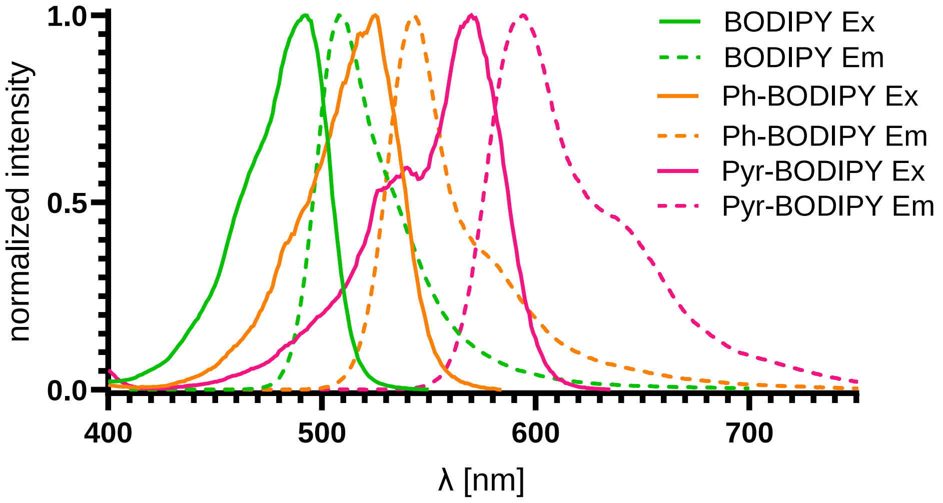

Figure S15. *Excitation and emission spectra in PBS pH 7.4 of unsubstituted BODIPY probes (green), 3-phenyl-substituted BODIPY probe (orange), and 3-pyrrolyl-substituted BODIPY probes (purple).*

Table S4. *Photophysical properties of the key probes. λ_{max} and quantum yield Φ were determined in PBS buffer pH 7.4 at 500 nM. Extinction was measured at 10 µM in EtOH to ensure a complete dissolution of all probes.*

Compound	λ_{max} Ex/Em [nm]	ε [M^{-1} cm^{-1}]	Φ [%]	Brightness [mM^{-1} cm^{-1}]
1	489 / 502	68,000	87.8	~ 60
1a	570 / 594	60,000	3.9	~ 2.4
2	489 / 502	32,000	69.0	~ 22
2a	569 / 594	44,000	2.0	~ 0.8
3	489 / 502	61,000	85.4	~ 52
4	490 / 501	57,000	82.5	~ 47
4a	570 / 594	47,000	4.1	~ 1.9
S22	521 / 537	48,000	88.4	~ 42
5	490 / 501	33,000	56.6	~ 19
5a	570 / 594	51,000	5.7	~ 2.9

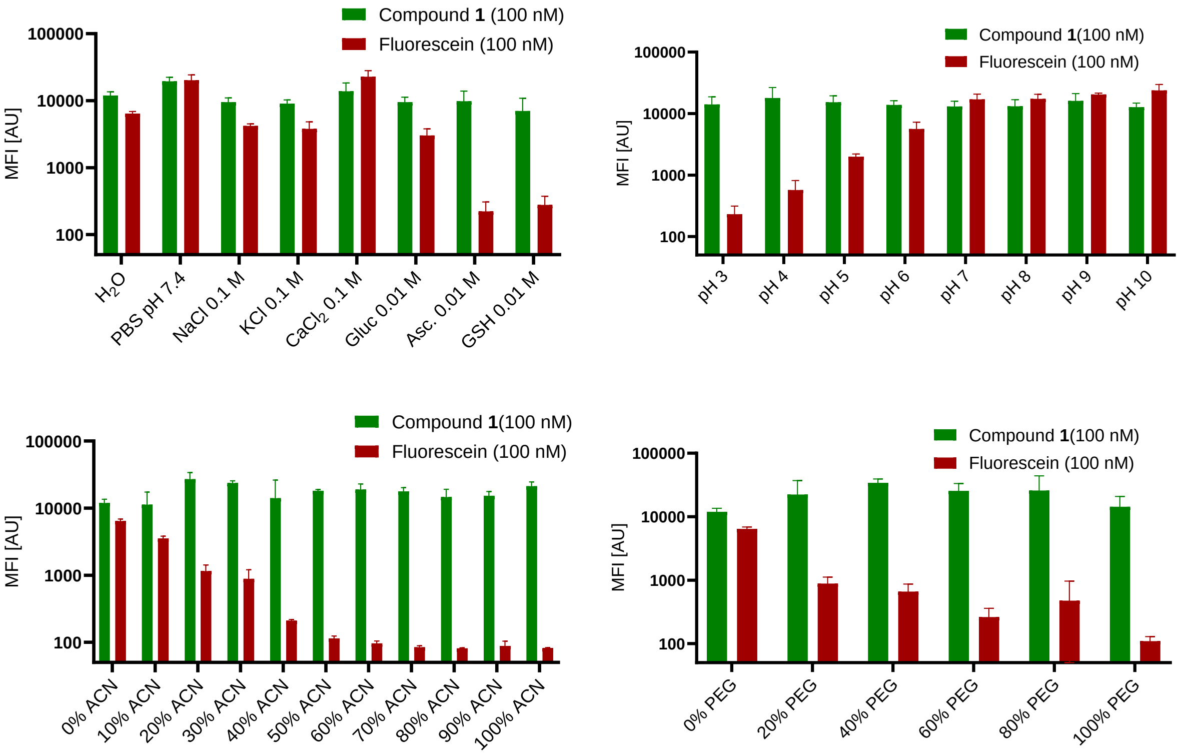

Figure S16. *Influence of chemical solvent and solutes on probe* ***1*** *in comparison to fluoresceine. A) Influence of PBS pH 7.4 buffer and other common ions and co-substrates in physiologically relevant concentrations. B) Influence of pH on fluorescence intensity C) Influence of lipophilicity (% acetonitrile (ACN) in water) on fluorescence intensity D) Influence of viscosity (% PEG-400 (η = 105-130 mPa s) in water) on fluorescence. Fluorescence intensity was measured in triplicate at λ_{Ex}=483 nm / λ_{Em}= 525 nm. Represented as bar chart with mean fluorescence intensity (MFI) ± SD.*

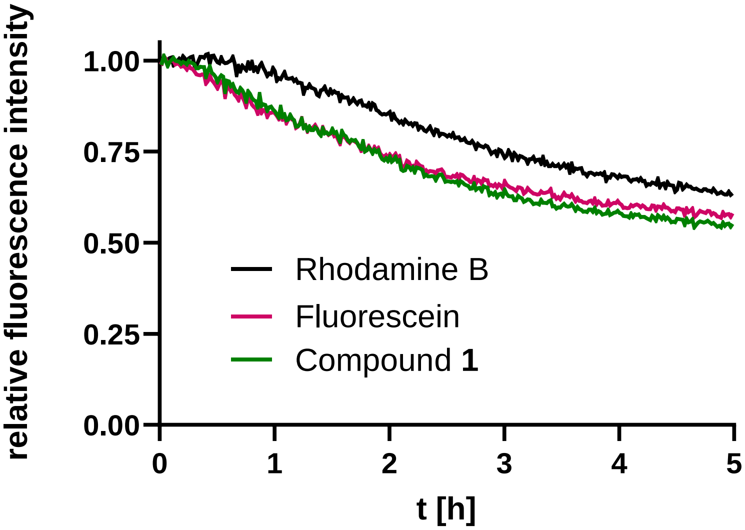

Figure S17. *Photostability of compound* ***1*** *compared to fluorescein and Rhodamine B. 500 nM solution of the respective fluorescent molecule in PBS pH 7.4 was irradiated at both FITC and Cy3 channel (488/525 nm and 561/590 nm, 10 nm bandwidth each) 300 times with 10 flashes each (from a high-energy Xenon arc discharge lamp with double monochromator setup, Tecan Safire 2) over five hours. The mean relative fluorescence of triplicate measurement of the FITC channel (****1*** *and fluorescein) or Cy3 channel (rhodamine B) with identical gain factor is represented over irradiation time.*

CEREP Data

Table S5. *Inhibition [%] of 50 clinically relevant and safety-related off-targets of key fluorescent probes at 10 µM concentration. Inhibition is visualized with a green to orange gradient. The fraction of targets with more than 50% S(50) and 90% S(90) per compound were calculated. Panel screen conducted at CEREPS Eurofin, France.*

Assay Description	4a	5	2	2a	1
A3 (h) (agonist radioligand)		17,70	45,00	34,80	-1,60
alpha2A (h) (antagonist radioligand)	7,40	-8,90	-2,20	-0,90	11,70
beta1 (h) (agonist radioligand)	-1,20	0,60	-1,70	-3,30	-3,30
beta2 (h) (antagonist radioligand)	-6,20	5,40	-0,60	1,80	-3,10
AT1 (h) (antagonist radioligand)	-12,50	11,10	-1,20	2,50	-4,30
BZD (central) (agonist radioligand)	-4,10	-16,30	-27,70	0,90	-11,90
CCK1 (CCKA) (h) (agonist radioligand)	-22,10	-24,70	-8,80	11,90	-3,80
D1 (h) (antagonist radioligand)	7,50	7,30	-1,20	6,10	8,10
glycine (strychnine-insensitive) (antagonist radioligand)	3,10	-20,30	6,50	-2,00	-6,70
M1 (h) (antagonist radioligand)	4,00	14,70	26,00	-0,70	33,50
M2 (h) (antagonist radioligand)	-4,90	33,20	22,20	-13,10	35,10
mu (MOP) (h) (agonist radioligand)	11,00	8,50	-0,40	7,60	4,00
PCP (antagonist radioligand)	3,60	-14,20	-5,50	-18,80	6,90
5-HT1A (h) (agonist radioligand)	-2,40	2,40	-29,40	-5,50	0,00
Ca2+ channel (L, diltiazem site) (antagonist radioligand)	6,20	1,70	-14,00	9,70	20,60
Cl- channel (GABA-gated) (antagonist radioligand)	40,30	20,90	51,90	40,80	41,90
xanthine oxidase/ superoxide O2- scavenging	24,10	-16,60	1,80	7,80	-29,70
HIV-1 protease	23,80	5,20	-1,90	8,30	10,30
norepinephrine transporter (h) (antagonist radioligand)	12,20	8,60	-0,50	6,30	7,40
acetylcholinesterase (h)	1,80	-14,40	-2,90	2,50	-8,20
5-HT3 (h) (antagonist radioligand)	1,10	1,40	-5,60	4,70	7,40
5-HT transporter (h) (antagonist radioligand)	-9,60	56,50	-4,80	-12,90	13,00
A1 (h) (agonist radioligand)	8,70	1,30	4,40	-1,00	-3,90
GR (h) (agonist radioligand)	48,10	17,10	9,10	26,30	13,90
5-HT2A (h) (agonist radioligand)	18,50	-9,20	-36,20	-6,80	-13,20
MMP-9 (h)	47,00	16,80	4,50	9,70	
PPARgamma (h) (agonist radioligand)	20,00	5,60	56,30	20,00	74,30
ZAP70 kinase (h)	5,40	6,90	-2,00	2,30	4,30
H1 (h) (antagonist radioligand)	1,30	51,50	5,50	4,40	30,40
AR(h) (agonist radioligand)	-1,40	-19,80	4,20	6,00	-14,20
N muscle-type (h) (antagonist radioligand)	-1,70	17,00	-0,30	-9,40	-11,30
H2 (h) (antagonist radioligand)	-34,80	-1,90	-18,70	-48,60	-10,70
D2S (h) (agonist radioligand)	3,30	12,60	-4,10	-2,60	5,90
H3 (h) (agonist radioligand)	1,80	-3,70	-7,20	7,30	5,20

5-HT2B (h) (agonist radioligand)	30,30	0,10	4,10	20,80	28,30
FP (h) (agonist radioligand)	16,30	10,40	6,50	4,40	11,60
alpha1A (h) (antagonist radioligand)	45,50	2,10	-4,60	-3,40	7,60
GSK3alpha (h)	-24,80	-1,80	-3,40	-5,80	0,30
GSK3beta (h)	-15,00	-1,20	-1,70	-1,50	3,20
CDK2 (h) (cycA)	-8,30	6,50	6,30	-5,80	7,80
N neuronal alpha4beta2 (h) (agonist radioligand)	-0,60	-16,60	-1,70	4,20	-18,30
Abl kinase (h)	-53,10	25,60	-10,50	-2,90	-1,90
ACE (h)	-21,20	-21,10	-15,80	-4,40	-10,40
PDE3B (h)	13,00	10,70	6,30	12,60	15,90
PDE4D2 (h)	10,80	11,30	-0,60	-10,80	18,00
Estrogen ER alpha (h) (agonist radioligand)	-2,50	35,00	-0,80	-13,70	17,80
COX2(h)	48,90	23,80	31,90	49,50	45,50
kappa (h) (KOP) (agonist radioligand)	22,70	10,30	5,90	4,90	7,80
CB1 (h) (agonist radioligand)	-2,70	9,90	-16,10	6,30	4,40
MAO-A (h) monoamine oxydase A, enzymatic assay	4,30	4,40	-1,30	-3,00	22,60
S(50) [%]	0.0	2.0	4.0	0.0	2.0
S(90) [%]	0.0	0.0	0.0	0.0	0.0

References

(1) Prokop, S.; Ábrányi-Balogh, P.; Barti, B.; Vámosi, M.; Zöldi, M.; Barna, L.; Urbán, G. M.; Tóth, A. D.; Dudok, B.; Egyed, A.; Deng, H.; Leggio, G. M.; Hunyady, L.; van der Stelt, M.; Keserű, G. M.; Katona, I. PharmacoSTORM Nanoscale Pharmacology Reveals Cariprazine Binding on Islands of Calleja Granule Cells. *Nat Commun* **2021**, *12* (1), 1–20. doi.org/10.1038/s41467-021-26757-z.

(2) Chang, J. W.; Cognetta, A. B.; Niphakis, M. J.; Cravatt, B. F. Proteome-Wide Reactivity Profiling Identifies Diverse Carbamate Chemotypes Tuned for Serine Hydrolase Inhibition. *ACS Chem Biol* **2013**, *8* (7), 1590–1599. doi.org/10.1021/cb400261h.

(3) Wang, Y.; Chanda, P.; Jones, P. G.; Kennedy, J. D. A Fluorescence-Based Assay for Monoacylglycerol Lipase Compatible with Inhibitor Screening. *Assay Drug Dev Technol* **2008**, *6* (3), 387–393. doi.org/10.1089/adt.2007.122.

(4) Miceli, M.; Casati, S.; Ottria, R.; Di Leo, S.; Eberini, I.; Palazzolo, L.; Parravicini, C.; Ciuffreda, P. Set-up and Validation of a High Throughput Screening Method for Human Monoacylglycerol Lipase (MAGL) Based on a New Red Fluorescent Probe. *Molecules* **2019**, *24* (12). doi.org/10.3390/molecules24122241.

(5) Deng, H.; Zhang, Q.; Lei, Q.; Yang, N.; Yang, K.; Jiang, J.; Yu, Z. Discovering Monoacylglycerol Lipase Inhibitors by a Combination of Fluorogenic Substrate Assay and Activity-Based Protein Profiling. *Front Pharmacol* **2022**, *13* (August), 1–16. doi.org/10.3389/fphar.2022.941522.

(6) McAllister, L. A.; Butler, C. R.; Mente, S.; O'Neil, S. V.; Fonseca, K. R.; Piro, J. R.; Cianfrogna, J. A.; Foley, T. L.; Gilbert, A. M.; Harris, A. R.; Helal, C. J.; Johnson, D. S.; Montgomery, J. I.; Nason, D. M.; Noell, S.; Pandit, J.; Rogers, B. N.; Samad, T. A.; Shaffer, C. L.; Da Silva, R. G.; Uccello, D. P.; Webb, D.; Brodney, M. A. Discovery of Trifluoromethyl Glycol Carbamates as Potent and Selective Covalent Monoacylglycerol Lipase (MAGL) Inhibitors for Treatment of Neuroinflammation. *J Med Chem* **2018**, *61* (7), 3008–3026. doi.org/10.1021/acs.jmedchem.8b00070.

(7) Cisar, J. S.; Weber, O. D.; Clapper, J. R.; Blankman, J. L.; Henry, C. L.; Simon, G. M.; Alexander, J. P.; Jones, T. K.; Ezekowitz, R. A. B.; O'Neill, G. P.; Grice, C. A. Identification of ABX-1431, a Selective Inhibitor of Monoacylglycerol Lipase and Clinical Candidate for Treatment of Neurological Disorders. *J Med Chem* **2018**, *61* (20), 9062–9084. doi.org/10.1021/acs.jmedchem.8b00951.

(8) Kemble, A. M.; Hornsperger, B.; Ruf, I.; Richter, H.; Benz, J.; Kuhn, B.; Heer, D.; Wittwer, M.; Engelhardt, B.; Grether, U.; Collin, L. A Potent and Selective Inhibitor for the Modulation of MAGL Activity in the Neurovasculature. *PLoS One* **2022**, *17* (9 September), 1–24. doi.org/10.1371/journal.pone.0268590.

(9) Wyatt, R. M.; Fraser, I.; Welty, N.; Lord, B.; Wennerholm, M.; Sutton, S.; Ameriks, M. K.; Dugovic, C.; Yun, S.; White, A.; Nguyen, L.; Koudriakova, T.; Tian, G.; Suarez, J.; Szewczuk, L.; Bonnette, W.; Ahn, K.; Ghosh, B.; Flores, C. M.; Connolly, P. J.; Zhu, B.; Macielag, M. J.; Brandt, M. R.; Chevalier, K.; Zhang, S. P.; Lovenberg, T.; Bonaventure, P. Pharmacologic Characterization of JNJ-42226314, [1-(4-Fluorophenyl)Indol-5-Yl]-[3-[4-(Thiazole-2-Carbonyl) Piperazin-1-Yl]Azetidin-1-Yl]Methanone, a Reversible, Selective, and Potent Monoacylglycerol Lipase Inhibitor. *J Pharmacol Exp Ther* **2020**, *372* (3), 339–353. doi.org/10.1124/JPET.119.262139.

(10) Long, J. Z.; Li, W.; Booker, L.; Burston, J. J.; Kinsey, S. G.; Schlosburg, J. E.; Pavón, F. J.; Serrano, A. M.; Selley, D. E.; Parsons, L. H.; Lichtman, A. H.; Cravatt, B. F. Selective Blockade of 2-Arachidonoylglycerol Hydrolysis Produces Cannabinoid Behavioral Effects. *Nat Chem Biol* **2009**, *5* (1), 37–44. doi.org/10.1038/nchembio.129.

(11) Jiang, M.; Huizenga, M. C. W.; Wirt, J. L.; Paloczi, J.; Amedi, A.; van den Berg, R. J. B. H. N.; Benz, J.; Collin, L.; Deng, H.; Di, X.; Driever, W. F.; Florea, B. I.; Grether, U.; Janssen, A. P. A.; Hankemeier, T.; Heitman, L. H.; Lam, T. W.; Mohr, F.; Pavlovic, A.; Ruf, I.; van den Hurk, H.; Stevens, A. F.; van der Vliet, D.; van der Wel, T.; Wittwer, M. B.; van Boeckel, C. A. A.; Pacher, P.; Hohmann, A. G.; van der Stelt, M. A Monoacylglycerol Lipase Inhibitor Showing

Therapeutic Efficacy in Mice without Central Side Effects or Dependence. *Nat Commun* **2023**, *14* (1). doi.org/10.1038/s41467-023-43606-3.

(12) Ikeda, S.; Sugiyama, H.; Tokuhara, H.; Murakami, M.; Nakamura, M.; Oguro, Y.; Aida, J.; Morishita, N.; Sogabe, S.; Dougan, D. R.; Gay, S. C.; Qin, L.; Arimura, N.; Takahashi, Y.; Sasaki, M.; Kamada, Y.; Aoyama, K.; Kimoto, K.; Kamata, M. Design and Synthesis of Novel Spiro Derivatives as Potent and Reversible Monoacylglycerol Lipase (MAGL) Inhibitors: Bioisosteric Transformation from 3-Oxo-3,4-Dihydro-2 H-Benzo[b][1,4]Oxazin-6-Yl Moiety. *J Med Chem* **2021**, *64* (15), 11014–11044. doi.org/10.1021/acs.jmedchem.1c00432.

(13) Benz, J.; Gobbi, L.; Grether, U.; Groebke Zbinden, K.; Hornsperger, B.; Kroll, C.; Kuhn, B.; Martin, R. E.; O'Hara, F.; Puellmann, B.; Richter, H.; Ritter, M. 4,4A,5,7,8,8A-Hexahydropyrido[4,3-b][1,4]Oxazin-3-One Compounds as MAGL Inhibitors and Their Preparation. WO2021048242, 2021.

(14) Kamata, Makoto; Sugiyama, Hideyuki; Nakamura, Minoru; Murakami, Masataka; Ikeda, Shuhei; Okawa, Tomohiro; Tokuhara, H. Preparation of Heterocyclic Compound as MAGL Inhibitor. WO2019065791, A1, 2019.

(15) Amoussa, Machoud; Benz, Joerg; Brian, Niels Kevin; Friston, Kallie; Giroud, Maude; Grether, Uwe; Groebke Zbinden, Katrin; Hornsperger, Benoit; Kroll, Carsten; Kuhn, Bernd; Leake, Camiel John; Martin, Rainer E.; Nippa, David Friedrich Erhard; O'Hara, Fionn, S. Preparation of Heterocyclic Compounds as Monoacylglycerol Lipase (MAGL) Inhibitors. WO2022223750, A1, 2022.

(16) Benz, Joerg; Grether, Uwe; Hornsperger, Benoit; Kroll, Carsten; Kuhn, Bernd; Martin, Rainer E.; O'Hara, Fionn; Puellmann, Bernd; Richter, Hans; Ritter, M. Preparation of Heterocyclic Compounds as Monoacylglycerol Lipase (MAGL) Inhibitors. WO2022049134, A1, 2022.

(17) Benz, Joerg; Decoret, Guillaume; Grether, Uwe; Groebke Zbinden, Katrin; Hornsperger, Benoit; Kuhn, Bernd; Richter, Hans; Rombach, Didier; O'Hara, Fionn; Kroll, C. Preparation of Heterocyclic Compounds as Inhibitors of Monoacylglycerol Lipase (MAGL). WO2020207941, A1, 2020.

(18) Cisar, J. S.; Weber, O. D.; Clapper, J. R.; Blankman, J. L.; Henry, C. L.; Simon, G. M.; Alexander, J. P.; Jones, T. K.; Ezekowitz, R. A. B.; O'Neill, G. P.; Grice, C. A. Identification of ABX-1431, a Selective Inhibitor of Monoacylglycerol Lipase and Clinical Candidate for Treatment of Neurological Disorders. *J Med Chem* **2018**, *61* (20), 9062–9084. doi.org/10.1021/acs.jmedchem.8b00951.

(19) Schaus, S. E.; Brandes, B. D.; Larrow, J. F.; Tokunaga, M.; Hansen, K. B.; Gould, A. E.; Furrow, M. E.; Jacobsen, E. N. Highly Selective Hydrolytic Kinetic Resolution of Terminal Epoxides Catalyzed by Chiral (Salen)CoIII Complexes. Practical Synthesis of Enantioenriched Terminal Epoxides and 1,2-Diols. *J Am Chem Soc* **2002**, *124* (7), 1307–1315. doi.org/10.1021/ja016737l.

(20) Gazzi, T.; Brennecke, B.; Olikauskas, V.; Hochstrasser, R.; Wang, H.; Keen Chao, S.; Atz, K.; Mostinski, Y.; Topp, A.; Heer, D.; Kaufmann, I.; Ritter, M.; Gobbi, L.; Hornsperger, B.; Wagner, B.; Richter, H.; O'Hara, F.; Wittwer, M. B.; Jul Hansen, D.; Collin, L.; Kuhn, B.; Benz, J.; Grether, U.; Nazaré, M. Development of a Highly Selective NanoBRET Probe to Assess MAGL Inhibition in Live Cells. *ChemBioChem* **2024**, e202400704 doi.org/10.1002/cbic.202400704.

(21) Benz, Joerg; Gazzi, T.; Gobbi, Luca; Grether, Uwe; Hornsperger, Benoit; Kroll, Carsten; Kuhn, Bernd; Mostinski, Yelena; Nazare, Marc; O'Hara, Fionn; Richter, H. Fluorescent Probes for Monoacylglycerol Lipase (Magl). WO2021058443, 2021.

(22) Alsenz, J.; Kansy, M. High Throughput Solubility Measurement in Drug Discovery and Development. *Adv Drug Deliv Rev* **2007**, *59* (7), 546–567. doi.org/10.1016/j.addr.2007.05.007.

(23) Schalk-Hihi, C.; Schubert, C.; Alexander, R.; Bayoumy, S.; Clemente, J. C.; Deckman, I.; DesJarlais, R. L.; Dzordzorme, K. C.; Flores, C. M.; Grasberger, B.; Kranz, J. K.; Lewandowski, F.; Liu, L.; Ma, H.; Maguire, D.; Macielag, M. J.; McDonnell, M. E.; Haarlander, T. M.; Miller, R.; Milligan, C.; Reynolds, C.; Kuo, L. C. Crystal Structure of a

Soluble Form of Human Monoglyceride Lipase in Complex with an Inhibitor at 1.35 A ° Resolution. *Protein Science* **2011**, *20* (4), 670–683. doi.org/10.1002/pro.596.

(24) Kabsch, W. Xds. *Acta Crystallogr D Biol Crystallogr* **2010**, *66* (2), 125–132.

(25) McCoy, A. J.; Grosse-Kunstleve, R. W.; Adams, P. D.; Winn, M. D.; Storoni, L. C.; Read, R. J. Phaser Crystallographic Software. *J Appl Crystallogr* **2007**, *40* (4), 658–674.

(26) Winn, M. D.; Ballard, C. C.; Cowtan, K. D.; Dodson, E. J.; Emsley, P.; Evans, P. R.; Keegan, R. M.; Krissinel, E. B.; Leslie, A. G. W.; McCoy, A. Overview of the CCP4 Suite and Current Developments. *Acta Crystallogr D Biol Crystallogr* **2011**, *67* (4), 235–242.

(27) Emsley, P.; Lohkamp, B.; Scott, W. G.; Cowtan, K. Features and Development of Coot. *Acta Crystallogr D Biol Crystallogr* **2010**, *66* (Pt 4), 486–501. doi.org/10.1107/S0907444910007493.

(28) Nikolovska-Coleska, Z.; Wang, R.; Fang, X.; Pan, H.; Tomita, Y.; Li, P.; Roller, P. P.; Krajewski, K.; Saito, N. G.; Stuckey, J. A.; Wang, S. Development and Optimization of a Binding Assay for the XIAP BIR3 Domain Using Fluorescence Polarization. *Anal Biochem* **2004**, *332* (2), 261–273. doi.org/10.1016/j.ab.2004.05.055.

(29) Burgalossi, A.; Jung, S. Y.; Man, K. nok M.; Nair, R.; Jockusch, W. J.; Wojcik, S. M.; Brose, N.; Rhee, J. S. Analysis of Neurotransmitter Release Mechanisms by Photolysis of Caged Ca2+ in an Autaptic Neuron Culture System. *Nat Protoc* **2012**, *7* (7), 1351–1365. doi.org/10.1038/nprot.2012.074.

(30) Preusser, F.; dos Santos, N.; Contzen, J.; Stachelscheid, H.; Costa, É. T.; Mergenthaler, P.; Preibisch, S. FRC-QE: A Robust and Comparable 3D Microscopy Image Quality Metric for Cleared Organoids. *Bioinformatics* **2021**, *37* (18), 3088–3090. doi.org/10.1093/bioinformatics/btab160.

(31) He, Y.; Schild, M.; Grether, U.; Benz, J.; Leibrock, L.; Heer, D.; Topp, A.; Collin, L.; Kuhn, B.; Wittwer, M.; Keller, C.; Gobbi, L. C.; Schibli, R.; Mu, L. Development of High Brain-Penetrant and Reversible Monoacylglycerol Lipase PET Tracers for Neuroimaging. *J Med Chem* **2022**, *65* (3), 2191–2207. doi.org/10.1021/acs.jmedchem.1c01706.
